# Prevalence of soil-transmitted helminth infections, schistosomiasis, and lymphatic filariasis before and after preventive chemotherapy initiation in the Philippines: A systematic review and meta-analysis

**DOI:** 10.1371/journal.pntd.0010026

**Published:** 2021-12-20

**Authors:** John Paul Caesar Robles delos Trinos, Luh Putu Lila Wulandari, Naomi Clarke, Vicente Belizario, John Kaldor, Susana Vaz Nery

**Affiliations:** 1 The Kirby Institute, UNSW Sydney, Sydney, New South Wales, Australia; 2 College of Public Health, University of the Philippines Manila, Manila, Philippines; 3 Faculty of Medicine, Universitas Udayana, Bali, Indonesia; Washington University School of Medicine, UNITED STATES

## Abstract

**Objective:**

To estimate the impact of preventive chemotherapy on the prevalence and intensity of soil-transmitted helminth (STH) infections, schistosomiasis, and lymphatic filariasis in the Philippines, using systematic review and meta-analysis.

**Methods:**

We included reports reporting prevalence of STH infections, schistosomiasis, or lymphatic filariasis in the Philippines published until 31 March 2021. Peer-reviewed studies were identified in electronic databases. Grey literature reports by the University of the Philippines and the Department of Health were also included. Pooled infection prevalence, before and after the initiation of preventive chemotherapy, stratified by age group, was calculated using the inverse variance heterogeneity model.

**Findings:**

A total of 109 reports were included in the review and meta-analysis. Overall prevalence of moderate-heavy intensity *Ascaris lumbricoides* (6.6%) and *Trichuris trichiura* (2.7%) infection after initiation of preventive chemotherapy were significantly lower than the prevalence prior to initiation (23.6% for *A*. *lumbricoides* and 12.2% for *T*. *trichiura*). Prevalence reductions were also found in school and preschool-age children for *A*. *lumbricoides* and *T*. *trichiura*. Studies conducted after preventive chemotherapy initiation had significantly lower overall prevalence of moderate-heavy intensity schistosomiasis (3.1% vs 0.2%) and of schistosomiasis in school-age children (30.5% vs 1%). Pooled prevalence of lymphatic filariasis prior to preventive chemotherapy initiation was 3.2% across 12 provinces, while currently only two provinces still have prevalence of more than 1%. There were no published studies reporting prevalence of lymphatic filariasis after initiation of preventive chemotherapy. Heterogeneity was high with I^2^ mostly above 90%.

**Conclusion:**

The burden of STH infections and schistosomiasis in children were significantly lower in studies conducted following the initiation of preventive chemotherapy. Eliminating morbidity and interrupting transmission, however, may require expanded control initiatives including community-wide treatment, and improved water, sanitation, and hygiene. Lymphatic filariasis burden has decreased since the implementation of preventive chemotherapy, with all but two provinces having reached the elimination of lymphatic filariasis as a public health problem.

## Author summary

Mass treatment with anti-parasitic medications is a key control and elimination strategy for several helminth infections, namely intestinal worm infections, schistosomiasis, and lymphatic filariasis, which are common in the Philippines and other endemic countries. To gain insight into the impact of such a strategy that has been in place for more than 14 years, we used systematic review and meta-analysis to compare the prevalence of each of these helminth infections in the Philippines before and after the initiation of mass treatment. The review included 109 reports, composed of peer-reviewed studies and grey literature. The pooled overall prevalence of heavy intensity and the prevalence in children of intestinal worm infection and schistosomiasis were significantly lower in studies conducted after initiation of mass treatment. The studies included are highly heterogenous reflecting variability in sampling procedures, diagnostic tests, study sites, and years of data collection. Eliminating morbidity and interrupting transmission may require expanded control initiatives including community-wide treatment, and improved water, sanitation, and hygiene. The same significantly lower prevalence after initiation of mass treatment was observed for lymphatic filariasis where only two out of the initial 12 endemic provinces still require mass treatment due to prevalence above 1%.

## Introduction

Neglected tropical diseases (NTDs) are a diverse group of infectious diseases that prevail in impoverished conditions in tropical and subtropical areas. NTDs affect 1.14 billion people and cost developing countries billions of dollars annually.[1, 2] Three groups of globally important NTDs are soil-transmitted helminth (STH) infections, schistosomiasis, and lymphatic filariasis.[3] All are helminth infections, and have in common the use of preventive chemotherapy as a key control strategy.

STHs include *Ascaris lumbricoides*, *Trichuris trichiura*, and the hookworms *Ancylostoma duodenale*, *Ancylostoma ceylanicum*, and *Necator americanus*.[4, 5] STH infections may lead to anaemia, nutrient malabsorption, malnutrition, and poor cognitive and physical development.[6–8] Groups at risk for STH infection include school-age children and preschool-age children.[3] STH infections were estimated to cause a disease burden of 2.59 million disability-adjusted life years (DALYs) and affected 894 million people in 2017 worldwide.[2, 9] The Philippines is considered endemic for all STH species infections.[10]

Schistosomiasis, caused by several species of *Schistosoma*, may result in morbidity including anaemia and stunting [11–13], while chronic cases may lead to hepatomegaly, portal hypertension, splenomegaly, and hypersplenism.[14] Schistosomiasis affected an estimated 143 million people worldwide in 2017, with a disease burden of 1.83 million DALYs.[2, 9] In the Philippines, schistosomiasis, caused by *Schistosoma japonicum*, has been designated as endemic in 190 municipalities in 28 provinces in 2007 prior to the implementation of nationwide preventive chemotherapy.[15]

Lymphatic filariasis, caused by filarial worms, results in lymphoedema, elephantiasis, and hydrocoele which may limit mobility and result in productivity loss and stigma.[16] Estimated disease burden in 2017 was 1.74 million DALYs, with 65 million people infected worldwide.[2, 9] In the Philippines, lymphatic filariasis, caused by *Wuchereria bancrofti* and *Brugia malayi*, has been designated endemic in 46 provinces in 2000 prior to the implementation of nationwide preventive chemotherapy, of which 31 have been declared as having eliminated lymphatic filariasis as a public health problem in recent years.[17]

Preventive chemotherapy, consisting of regular large-scale distribution of anthelmintics is the main strategy for control and elimination of human helminthiasis.[18] Mass drug administration (MDA), which involves treating the entire population of an area, and targeted chemotherapy, where only specific at-risk groups (such as school-age children) are treated, are among the modes of preventive chemotherapy.[18] Lymphatic filariasis control programs involve MDA, while those for STH infections and schistosomiasis control typically use targeted drug administration, mostly focused on school-age children.[18]

In the Philippines, nationwide biannual targeted preventive chemotherapy using albendazole for preschool- and school-age children, through school-based programs, currently including high schools, has been implemented since 2006 to control STH infection. Annual MDA of praziquantel in individuals aged 5 years and above in endemic barangays (villages) has been implemented since 2007 to control schistosomiasis. Annual MDA of diethylcarbamazine plus albendazole in endemic provinces has been implemented since 2001 to eliminate lymphatic filariasis.[10, 19, 20]

Despite years of implementation, there is limited information on the impact of preventive chemotherapy on the prevalence of these helminthiases in the Philippines. To gain insight into program impact, we used the methods of systematic review and meta-analysis [21, 22] to compare the prevalence of STH infections, schistosomiasis, and lymphatic filariasis in the Philippines before and after the initiation of large-scale preventive chemotherapy.

## Methods

This systematic review and meta-analysis adhered to the Preferred Reporting Items for Systematic Reviews and Meta-Analysis (PRISMA) guidelines [23], shown in S1 PRISMA Checklist. The protocol was registered in PROSPERO [CRD42018091555] where it can also be accessed.

### Inclusion and exclusion criteria and search strategy

We included original research conducted (for grey literature) or published up to 31 March 2021 that reported prevalence of STH infections, schistosomiasis, and/or lymphatic filariasis in the Philippines. Review articles, commentaries, conference proceeding abstracts, and case reports were excluded.

For peer-reviewed studies, we searched in Medline, Embase, Emcare, Ovid Global Health, Cumulated Index to Nursing and Allied Health Literature (CINAHL), Health Research and Development Information Network (HERDIN) Philippines, Directory of Open Access Journals, Scopus, and Institute for Scientific Information Web of Science on 31 March 2021. Unpublished studies by the University of the Philippines Manila provided by one of the authors (VB) and surveys by the Philippine Department of Health provided on request by the national STH control program manager were also included. Table 1 describes the search strategy while S1 Text shows the search strings used.

**Table 1:** Search strategy.

Database	Keyword combination	Filters
Medline	diseases AND location AND epidemiology/intervention	by keyword and MeSH headings
Embase		by keyword and MeSH headings
Ovid Global Health		by keyword and MeSH headings
Scopus		by research articles published in academic journals in the fields of Medicine and (Immunology and Microbiology)
Emcare	diseases AND location	by keyword and MeSH headings
CINAHL		
Web of Science		by topic; research articles in the fields of parasitology, tropical medicine, public environmental and occupational health, and infectious diseases
Directory of Open Access Journals		by research articles
HERDIN	diseases	by research articles

### Study screening and selection

Records were managed in EndNote X9. Removal of duplicates, title and abstract screening, and full-text screening were done by one author (JPCDT). For quality control, 10% of the studies subjected to title and abstract screening and 10% of the studies subjected to full-text screening were randomly selected and reviewed by another author (LPLW) to determine inconsistencies in exclusion or inclusion.

### Data extraction and processing

Extracted data included bibliographic details, methods (study design, study sites, number of clusters, population studied, year of data collection, diagnostic test used), and results (number of participants examined, number of participants positive, odds ratio or relative risk for any risk factors for infection examined in the study). The study sites were defined as the provinces or highly urbanised cities where the study was conducted. The population studied was defined as the population subgroups examined in the study, which included preschool-age children (1–5 years old), school-age children (6–18 years old), children (1–18 years old), adults (more than 18 years old), or general population. Moderate and heavy intensity STH infections were combined into moderate-heavy intensity STH infections, as defined by World Health Organization (WHO).[24] Data extraction was performed by JPCDT in duplicate. Twenty percent of the studies included in data extraction were randomly selected with data extraction cross-checked by LPLW.

Studies that included multiple study sites, collected data in different years, or examined different population groups contributed multiple prevalence estimates to the review. If prevalence estimates utilised multiple diagnostic tests, only the estimate that used the most common tests, namely Kato-Katz technique for STH infections, Kato-Katz technique or circumoval precipitin test (COPT) for schistosomiasis, and nocturnal blood microscopy using Giemsa stain for lymphatic filariasis, were included.[25–27] A prevalence estimate was categorised as “pre-preventive chemotherapy initiation” if data were collected on or before the following cut-off years based on program implementation: 2006 for STH infections, 2007 for schistosomiasis, and 2001 for lymphatic filariasis, or if data were collected before a preventive chemotherapy intervention as part of a study. Otherwise, the prevalence estimate was categorised as “post-preventive chemotherapy initiation”. In 15 studies which did not report the year of data collection, the year of publication was assumed to be the year of data collection.

Study quality was assessed using the Joanna Briggs Institute’s Critical Appraisal Checklist for Prevalence Studies. [28] Studies were assessed against nine criteria encompassing internal and external validity.

### Data analysis

Studies were summarised by calculating the proportions of variables of interest, including: population group studied, diagnostic tests used, species reported, study site, and year of data collection.

Meta-analyses were performed in MetaXL v5.3 (EpiGear International, Noosa, Australia).[29] An inverse variance heterogeneity model was used considering the high heterogeneity expected across the prevalence estimates. This model, which was also used in previous NTD meta-analyses [30, 31], addresses the limitations of the random effects model, which underestimates statistical error and generates overconfident estimates when dealing with heterogeneous studies. The inverse variance heterogeneity model uses the inverse of the variance of each study as weights, thereby giving studies with high variance less weight than less heterogenous studies.[29]

The pooled “overall pre-preventive chemotherapy initiation prevalence” and pooled “overall post-preventive chemotherapy initiation prevalence” for each species and infection intensity were calculated using all prevalence estimates, regardless of population group examined. Separate analyses were then conducted to calculate the pooled prevalence for each population group. The range, 95% confidence interval, and Higgins’ I^2^ were also obtained. The “overall post-preventive chemotherapy initiation prevalence” was considered significantly different from the “overall pre-preventive chemotherapy initiation prevalence” if the 95% confidence intervals of the two estimates did not overlap. One-way sensitivity analyses were conducted and included restricting the analysis to: 1) studies that used Kato-Katz technique to determine prevalence of STH infections and schistosomiasis, 2) only include provinces with both pre- and post-preventive chemotherapy initiation prevalence data, and 3) only include prevalence estimates obtained within five years before or after the initiation of preventive chemotherapy. Aside from Kato-Katz technique, it was not possible to restrict the analysis to other diagnostic tests due to the few studies which used these tests. It was not possible to perform a meta-analysis on the prevalence of lymphatic filariasis after preventive chemotherapy initiation due to the lack of prevalence estimates. Likewise, it was not possible to perform a meta-analysis on the relative risk and/or odds ratio of risk factors associated with increased risk for STH infection, schistosomiasis, and lymphatic filariasis given the few studies reporting these parameters. Therefore, the relative risks and/or odds ratio of risk factors are reported descriptive only.

## Results

**Fig 1:**
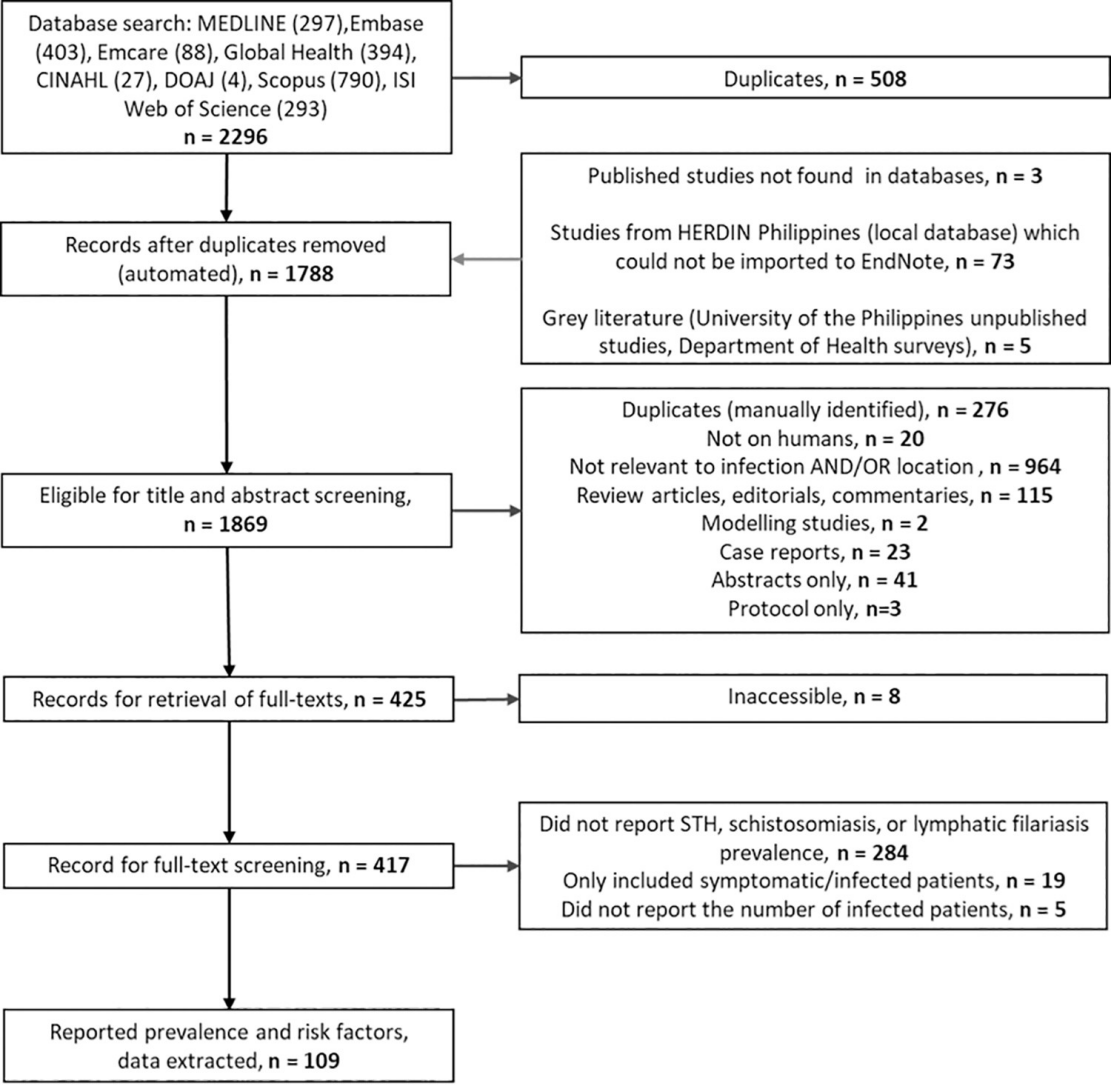
Flow diagram describing the database search, screening, and data extraction.

A total of 109 studies, containing 453 prevalence estimates, were included in the review (Fig 1). Most studies were cross-sectional (87 studies, 79.8%). A further 18 (16.5%) were repeated cross-sectional studies before and after an intervention (preventive chemotherapy for three [32–34], and the remainder reporting on the use of molluscicides, environmental modifications, or selective treatment). One study (0.9%) was a longitudinal cohort, and three (2.8%) were controlled trials.

In terms of study quality, the most common deficiency is whether appropriate statistical analysis was employed (62 out of 109 studies) with most studies particularly the older studies not reporting the confidence intervals. On the other hand, the most common items not reported were the sampling procedure (36 out of 109) and the sample size (46 out of 109). All other deficiencies or failure to report were less common (see S1 Table).

### STH infections

#### Descriptive analysis

Of the 67 studies (274 prevalence estimates) reporting prevalence of STH alone or with conjunction with schistosomiasis (see S2 and S3 Tables), 62 (217 prevalence estimates) reported prevalence of *A*. *lumbricoides*, with four (103 prevalence estimates) being grey literature. There were 58 studies (212 prevalence estimates) which reported prevalence of *T*. *trichiura*, of which four (103 prevalence estimates) were from grey literature. Overall, 52 studies (199 prevalence estimates) reported prevalence of hookworm, of which three studies (102 prevalence estimates) were from grey literature. There were 17 studies (154 prevalence estimates) which reported prevalence of moderate-heavy STH infections.

The most common diagnostic technique was the Kato-Katz and/or Kato thick smear technique (240 prevalence estimates, 87.6%), followed by formalin-ether concentration technique (FECT) (9 prevalence estimates, 3.3%). Other techniques used include direct fecal smear (DFS) (6 prevalence estimates, 1.8%), DFS plus FECT (3 prevalence estimates, 1.1%), merthiolate-iodine-formaldehyde (MIFC) concentration technique (2 prevalence estimates, 0.7%), and combined DFS, FECT, and Harada-Mori technique (2 prevalence estimates, 0.7%). The following diagnostic techniques were only used for 1 prevalence estimate (0.4%) each: Harada-Mori technique plus real-time quantitative PCR (qPCR), Kato-Katz plus qPCR, sodium acetate-acetic acid-formalin (SAF) plus Kato Katz technique, FECT plus Harada-Mori technique, MIFC plus Stoll technique, and combination of Kato thick, DFS, and FECT. For six prevalence estimates (2.2%), the stool examination technique used was not reported.

School-age children were the most studied group (178 prevalence estimates, 65.0%) followed by general population (48 prevalence estimates, 17.5%). There were 16 prevalence estimates (5.8%) each on children (i.e., preschool-age children and school-age children), preschool-age children, and adults. Most (224 prevalence estimates, 81.8%) were conducted after preventive chemotherapy initiation. The most recent prevalence estimate was in 2018, while the earliest was in 1946.

**Fig 2:**
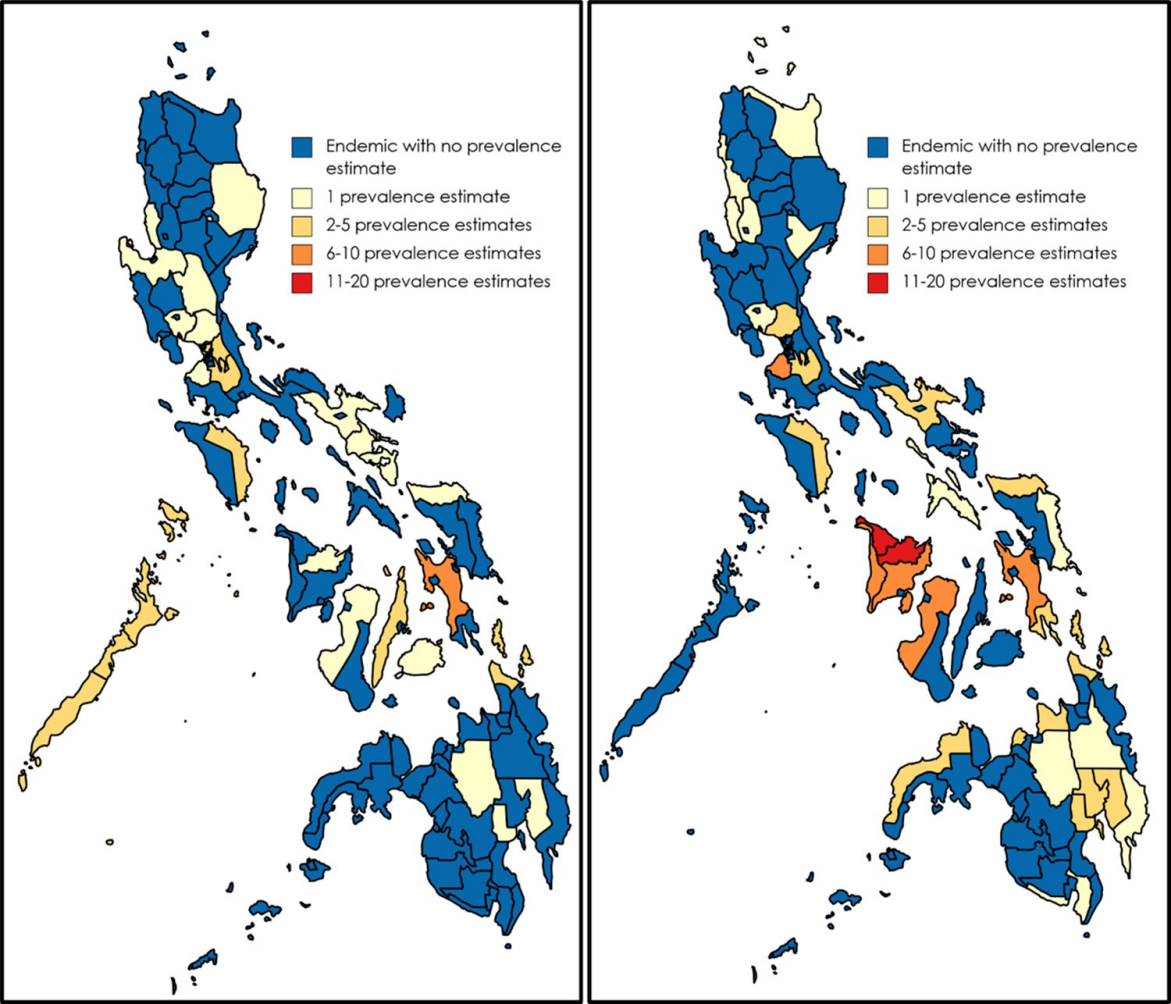
Sites of STH prevalence estimates pre-(left) and post-preventive chemotherapy (right) initiation. Created using MapChart (https://mapchart.net/index.html

A nationwide study conducted in 2013–2015 contributed 82 out of 274 prevalence estimates reporting STH prevalence.[35] Excluding this study, the 192 prevalence estimates were in 52 provinces or highly urbanised cities. This covers 16 out of the 17 STH-endemic regions. Leyte (18 prevalence estimates, 9.4%), Capiz (15 prevalence estimates, 7.8%), and Aklan (12 prevalence estimates, 6.3%) were the most common study sites (Fig 2).

Two studies reported estimates of risk factors for STH infections.[36, 37] Among these studies, it was reported that females and those who are wealthy (defined as living in a house with a cement floor, a galvanized roof, cement walls, and a tile/marble floor)[36] had a significantly lower risk of STH infections. On the other hand, having high school/vocational degree or less, having contact with water in rivers, being poor (defined as living in a house with a nipa/palm roof, a soil floor, and without cement walls)[36], having schistosomiasis, and having at least one child with STH infection was associated with a significantly higher risk of STH infection (S4 Table).

#### Meta-analysis

The overall prevalence prior to initiation of preventive chemotherapy was 45.2% for *A*. *lumbricoides*, 40.4% for *T*. *trichiura*, and 17.0% for hookworms. The overall prevalence after initiation of preventive chemotherapy was 23.8% for *A*. *lumbricoides*, 32.0% for *T*. *trichiura*, and 7.3% for hookworm. No significant difference was observed in the overall prevalence prior to initiation of preventive chemotherapy when compared with prevalence after initiation of preventive chemotherapy, with observed wide ranges in the prevalence estimates. In studies conducted after preventive chemotherapy initiation, significantly lower pooled prevalence was observed in preschool-age children, school-age children, and children for *A*. *lumbricoides* and in preschool-age children and school-age children for *T*. *trichiura*, compared to the prevalence prior to initiation of preventive chemotherapy. When analysis was restricted to prevalence estimates using the Kato-Katz technique, a significantly lower prevalence of *T*. *trichiura* in children after the initiation of preventive chemotherapy compared to prior to the initiation of preventive chemotherapy was also observed (Table 2). The overall prevalence prior to preventive chemotherapy initiation was 23.6% for moderate-heavy intensity *A*. *lumbricoides*, 12.2% for moderate-heavy intensity *T*. *trichiura*, and 0.2% for moderate-heavy intensity hookworm. The overall prevalence after preventive chemotherapy initiation of moderate-heavy intensity *A*. *lumbricoides* (6.6%) and of moderate-heavy intensity *T*. *trichiura* (2.7%) were significantly lower than the prevalence prior to preventive chemotherapy initiation. The overall prevalence after preventive chemotherapy initiation of moderate-heavy intensity hookworm infection was 0.1%. Heterogeneity was very high with I^2^ mostly above 90%. Wide prevalence ranges were observed (Table 2).

**Table 2:** Meta-analyses of prevalence of *A*. *lumbricoides*, *T*. *trichiura*, hookworm by preventive chemotherapy status and population (1946–2018).

INFECTION /GROUP	ALL DIAGNOSTIC TESTS								KATO-KATZ/KATO THICK ONLY							
	PRE-PC				POST-PC				PRE-PC				POST-PC			
	N	Prev	95% CI	Range	N	Prev	95% CI	Range	N	Prev	95% CI	Range	N	Prev	95% CI	Range
***A*. *lumbricoides***																
Preschool-age children^~^	1	2.9	(0.4–7.3)	N/A	15	30.9	(19.4–43.1)	(3.9–59.0)	-	N/A	N/A	N/A	15	30.9	(19.4–43.1)	(3.9–59.0)
School-age children**	14	63.2	(41.9–83.2)	(21.0–97.4)	112	19.4	(16.1–22.7)	(0.0–84.1)	10	45.6	(33.5–57.8)	(21.0–74.5)	110	19.1	(15.9–22.4)	(0.0–84.1)
Children**	11	50.1	(37.0–63.1)	(28.7–80.3)	4	17.3	(3.6–33.8)	(2.1–34.1)	7	45.6	(35.1–56.1)	(28.7–64.2)	4	17.3	(3.6–33.8)	(2.1–34.1)
Adults	3	56.1	(0.0–100.0)	(6.3–59.3)	7	19.1	(8.9–30.4)	(3.1–53.3)	1	6.3	(0.1–17.9)	N/A	7	19.1	(8.9–30.4)	(3.1–53.3)
General population	21	39.9	(1.2–84.8)	(20.7–94.0)	29	27.1	(14.4–40.9)	(0.0–84.5)	4	26.8	(0.0–100.0)	(20.7–82.0)	24	27.4	(14.3–41.6)	(0.0–40.3)
Overall	50	45.2	(19.2–72.0)	(2.9–97.4)	167	23.8	(17.5–30.4)	(0.0–84.1)	22	30.6	(0.0–73.2)	(6.3–82.0)	160	23.8	(17.4–30.5)	(0.0–84.1)
**MHI *A*. *lumbricoides*** **	13	23.6	(16.4–31.3)	(3.9–46.5)	140	6.6	(5.0–8.3)	(0.0–66.2)	13	23.6	(16.4–31.3)	(3.9–46.5)	140	6.6	(5.0–8.3)	(0.0–66.2)
***T*. *trichiura***																
Preschool-age children^~^	1	2.0	(0.0–5.8)	N/A	15	24.2	(11.2–38.6)	(3.9–55.1)	-	N/A	N/A	N/A	15	24.2	(11.2–38.6)	(3.9–55.1)
School-age children**	13	65.1	(39.4–88.6)	(14.5–93.5)	112	23.5	(19.2–28.0)	(0.5–94.4)	10	48.9	(30.8–67.1)	(14.5–92.5)	110	23.3	(19.0–27.8)	(0.5–94.4)
Children^#^	11	41.3	(14.8–69.2)	(16.8–92.4)	4	18.0	(0.0–47.3)	(0.2–53.7)	6	52.3	(44.6–60.0)	(38.1–60.8)	4	18.0	(0.0–47.3)	(0.2–53.7)
Adults	3	*23.6	(13.2–34.8)	(15.0–24.1)	7	24.9	(10.0–41.5)	(7.2–63.2)	1	18.8	(6.8–34.4)	N/A	7	24.9	(10.0–41.5)	(7.2–63.2)
General population	19	36.8	(0.0–94.8)	(11.8–95.0)	27	41.9	(20.6–64.0)	(0.0–62.4)	3	20.8	(0.0–100.0)	(16.3–83.2)	24	42.4	(20.5–65.1)	(0.0–62.4)
Overall	48	40.4	(9.0–74.4)	(2.0–95.0)	164	32.0	(22.3–42.1)	(0.0–94.4)	20	26.6	(0.0–78.5)	(14.5–92.5)	160	32.4	(22.5–42.8)	(0.0–94.4)
**MHI *T*. *trichiura*** **	13	12.2	(6.6–18.4)	(0.9–35.3)	140	2.7	(1.9–3.7)	(0.0–48.7)	13	12.2	(6.6–18.4)	(0.9–35.3)	140	2.7	(1.9–3.7)	(0.0–48.7)
**Hookworm**																
Preschool-age children	-	N/A	N/A	N/A	7	*1.1	(0.3–2.1)	(0.0–2.2)	-	N/A	N/A	N/A	7	*1.1	(0.3–2.1)	(0.0–2.2)
School-age children	12	6.3	(0.5–14.1)	(0.2–46.3)	109	0.9	(0.5–1.4)	(0.0–55.2)	9	6.0	(0.1–14.4)	(0.2–46.3)	108	0.9	(0.5–1.3)	(0.0–55.2)
Children	11	5.2	(2.1–8.9)	(0.6–23.4)	3	5.1	(0.0–12.6)	(1.2–11.9)	6	*4.4	(2.0–7.2)	(0.6–8.7)	3	5.1	(0.0–12.6)	(1.2–11.9)
Adults	2	7.9	(0.0–70.0)	(6.6–46.9)	6	6.5	(2.3–11.4)	(1.4–17.0)	-	N/A	N/A	N/A	7	5.8	(2.2–10.1)	(1.4–17.0)
General population	19	23.2	(0.0–62.8)	(0.7–72.9)	29	16.6	(5.7–29.1)	(0.0–52.8)	3	12.8	(0.0–39.8)	(11.4–39.0)	24	16.4	(5.3–29.2)	(0.0–31.3)
Overall	44	17.0	(1.2–37.5)	(0.2–72.9)	155	7.3	(2.9–12.3)	(0.0–55.2)	18	10.9	(0.0–29.0)	(0.2–46.3)	149	7.1	(2.7–12.1)	(0.0–55.2)
**MHI hookworm**	13	*0.2	(0.0–0.4)	(0.0–1.9)	119	*0.1	(0.1–0.1)	(0.0–2.1)	13	*0.2	(0.0–0.4)	(0.0–1.9)	118	*0.1	(0.1–0.1)	(0.0–2.1)

Preschool-age children (1–5 years old), school-age children (6–18 years old), children (1–18 years old), adults (more than 18 years old), general population (any age), overall (includes all prevalence estimates), PC—preventive chemotherapy, N—number of prevalence estimates, Prev–pooled prevalence, CI—confidence interval, MHI—moderate to heavy intensity

** significantly different between pre- and post-PC studies, N/A not applicable, ^~^ significant difference only in “all diagnostic tests”

# significant difference only in “Kato Katz/Kato thick”

*- I^2^ below 90

Leyte was the only province where enough prevalence estimates for STH infections both prior and after preventive chemotherapy initiation were available to allow meta-analysis. The overall prevalence prior and after preventive chemotherapy initiation in Leyte, respectively, were: 79.6% and 39.3% for *A*. *lumbricoides*, 84.3% and 54.8% for *T*. *trichiura*, and 42.9% and 8.2% for hookworms. Reductions in prevalence of all STH species were found (Table 3). When meta-analysis was restricted to estimates obtained within five years before or after preventive chemotherapy, no reduction in prevalence of STH was observed (Table 4).

**Table 3:** Meta-analyses of prevalence of *A*. *lumbricoides*, *T*. *trichiura*, and hookworm by preventive chemotherapy status and population group in Leyte province (1955–2015).

INFECTION/GROUP	ALL DIAGNOSTIC TESTS							
	PRE-PC INITIATION				POST-PC INITIATION			
	N	Prev	95% CI	Range	N	Prev	95% CI	Range
***A*. *lumbricoides***								
School-age children	3	64.4	(47.6–80.4)	(51.7–74.5)	4	43.9	(16.2–72.6)	(16.9–79.3)
Overall**	9	79.6	(70.5–88.1)	(51.7–94.0)	8	39.3	(24.9–54.1)	(16.9–79.3)
***T*. *trichiura***								
School-age children	3	75.1	(36.2–100.0)	(47.0–92.5)	4	58.2	(26.3–88.3)	(28.4–92.5)
Overall**	9	84.3	(72.7–94.3)	(47.0–95.0)	8	54.8	(38.9–70.5)	(28.4–92.5)
**Hookworm**								
School-age children	3	26.0	(0.0–59.7)	(7.4–46.3)	4	12.1	(0.0–39.9)	(0.5–55.2)
Overall**	9	42.9	(17.4–69.4)	(7.4–65.9)	8	8.2	(0.1–20.1)	(0.5–55.2)

School-age children (6–18 years old), overall (includes all prevalence estimates), PC—preventive chemotherapy, N—number of prevalence estimates, Prev—prevalence, CI—confidence interval

** significantly different between pre- and post-PC studies

**Table 4:** Meta-analyses of prevalence of *A*. *lumbricoides*, *T*. *trichiura*, and hookworm by preventive chemotherapy status and population group, pre-PC initiation restricted to 5 years before PC and post-PC initiation restricted to 5 years post-PC.

INFECTION	PRE-PC INITIATION				POST-PC INITIATION			
	N	Prev	95% CI	Range	N	Prev	95% CI	Range
***A*. *lumbricoides***								
Overall	13	26.1	(1.6–56.7)	(10.1–74.5)	42	28.5	(18.7–38.9)	(2.1–84.1)
Moderate-Heavy intensity	7	21.3	(11.6–31.9)	(3.9–40.7)	28	11.6	(7.2–16.3)	(0.0–66.2)
***T*. *trichiura***								
Overall	15	22.2	(0.0–64.9)	(14.5–92.5)	40	34.9	(22.8–47.6)	(0.2–94.4)
Moderate-Heavy intensity	7	9.7	(3.0–17.8)	(0.9–29.8)	27	5.1	(2.4–8.1)	(0.0–34.3)
***Hookworm***								
Overall	14	9.9	(0.0–26.7)	(0.2–46.9)	39	4.6	(0.5–9.9)	(0.0–55.2)
Moderate-Heavy intensity	7	*0.3	(0.0–0.6)	(0.0–1.9)	17	*0.2	(0.1–0.3)	(0.0–2.1)

PC—preventive chemotherapy, N—number of prevalence estimates, Prev—prevalence, CI—confidence interval, MHI—moderate to heavy intensity

*- I^2^ below 90

### Schistosomiasis

#### Descriptive analysis

There were 46 studies corresponding to 244 prevalence estimates that reported prevalence of schistosomiasis (see S2 and S5 Tables). Of these, three studies and 102 prevalence estimates were from grey literature. There were seven studies corresponding to 106 prevalence estimates that reported prevalence of moderate-heavy intensity schistosomiasis, all used Kato-Katz technique.

Kato-Katz and/or Kato thick smear (162 prevalence estimates, 66.4%) were the most common diagnostic techniques used, followed by COPT (17 prevalence estimates, 6.97%), merthiolate-formaldehyde concentration technique (MFCT) (14 prevalence estimates, 5.74%), and MIFC plus COPT (8 prevalence estimates, 3.3%). FECT, ELISA antibody, ELISA antigen, and ELISA (unspecified) were each used in 5 prevalence estimates (2.1%). Digital droplet PCR (4 prevalence estimates, 1.6), qPCR (3 prevalence estimates, 1.2%), and the combination of DFS, FECT, and Harada-Mori technique (2 prevalence estimates, 0.8%) were also used. The following diagnostic techniques were only used for 1 prevalence estimate (0.4%) each: DFS, FECT plus Harada-Mori technique, Kato-thick plus MIFC, Kato-Katz plus ultrasonography, MIFC, and MIFC plus Stoll technique. There were eight prevalence estimates (3.28%) where the stool examination technique used was not specified.

School-age children was the most studied group (161 prevalence estimates, 65.98%) followed by general population (73 prevalence estimates, 29.92%). There were six (2.46%) and three (1.23%) prevalence estimates on preschool-age children and children, respectively, and only one prevalence estimate on adults (0.41%). Most prevalence estimates (154 prevalence estimates, 63.11%) were conducted after preventive chemotherapy initiation. The most recent prevalence estimate was in 2018, while the earliest was in 1954.

**Fig 3:**
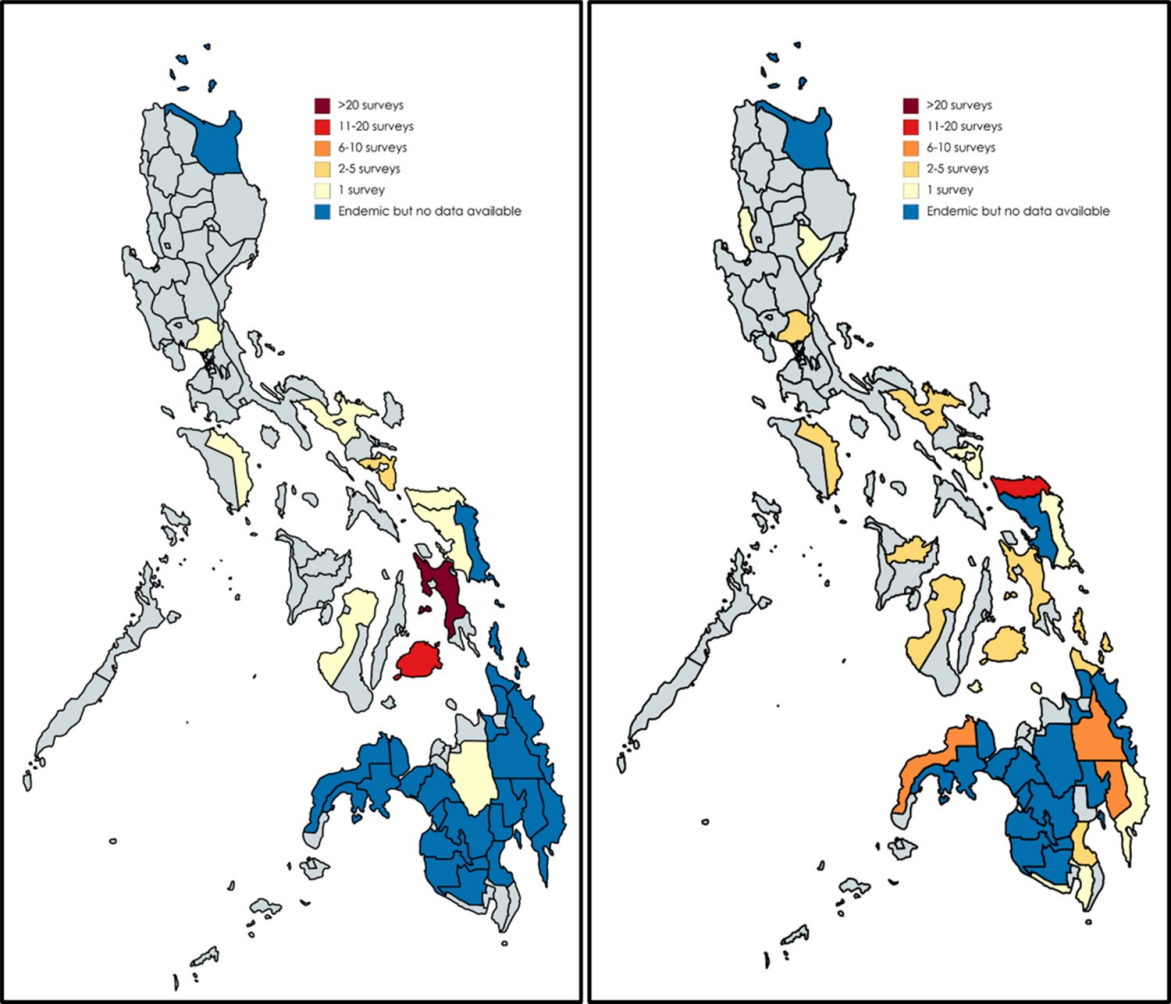
Sites of schistosomiasis prevalence estimates pre-(left) and post-preventive chemotherapy (right) initiation. Created using MapChart (https://mapchart.net/index.html.

There was a nationwide study which contributed 82 out of 244 prevalence estimates reporting prevalence of schistosomiasis. Excluding this nationwide study [35], the 162 prevalence estimates covered 25 provinces, representing 7 out of the 12 schistosomiasis-endemic regions. Leyte (60 prevalence estimates, 35.93%), Bohol (25 prevalence estimates, 14.97%), and Northern Samar (15 prevalence estimates, 8.98%) were the most common study sites (Fig 3).

#### Meta-analysis

The overall prevalence of schistosomiasis prior to preventive chemotherapy initiation was 8.4%, while it was 7.2% after preventive chemotherapy initiation. A significantly lower pooled prevalence was observed in school-age children in studies conducted after the initiation of preventive chemotherapy compared to those conducted prior to the initiation of preventive chemotherapy, although this was no longer observed when the analysis was restricted to prevalence estimates only using Kato-Katz technique (Table 5). The overall prevalence of moderate-heavy intensity schistosomiasis after preventive chemotherapy initiation (0.2%) was significantly lower than the prevalence prior to preventive chemotherapy initiation (3.1%). Heterogeneity was high with I^2^ mostly above 90%. Wide prevalence ranges were observed (Table 5).

**Table 5:** Meta-analyses of prevalence of *Schistosoma japonicum* by preventive chemotherapy status and population group (1954–2018).

INFECTION /GROUP	ALL DIAGNOSTIC TESTS								KATO-KATZ/KATO THICK ONLY							
	PRE-PC INITIATION				POST-PC INITIATION				PRE-PC INITIATION				POST-PC INITIATION			
	N	Prev	95% CI	Range	N	Prev	95% CI	Range	N	Prev	95% CI	Range	N	Prev	95% CI	Range
Preschool-age children	-	N/A	N/A	N/A	6	*0.6	(0.0–1.6)	(0.0–3.2)	-	N/A	N/A	N/A	6	*0.6	(0.0–1.6)	(0.0–3.2)
School-age children^~^	41	30.5	(23.5–37.8)	(0.0–78.0)	96	1.0	(0.4–1.7)	(0.0–86.0)	8	5.0	(0.0–16.7)	(0.0–78.0)	97	1.2	(0.4–2.0)	(0.0–79.3)
Children	3	*9.3	(4.3–14.9)	(5.7–13.9)	1	11.3	(8.7–14.1)	N/A	-	N/A	N/A	N/A	1	11.3	(8.7–14.1)	N/A
Adults	-	N/A	N/A	N/A	1	13.4	(10.3–16.8)	N/A	-	N/A	N/A	N/A	1	13.4	(10.3–16.8)	N/A
General population^#^	36	4.3	(0.8–8.8)	(0.1–43.2)	29	16.3	(3.3–32.0)	(0.0–90.6)	19	1.8	(0.1–4.2)	(0.1–38.9)	28	16.1	(4.4–29.9)	(0.0–39.8)
Overall	80	8.4	(3.5–14.0)	(0.0–79.3)	133	7.2	(2.2–13.30)	(0.0–90.6)	28	2.0	(0.1–4.6)	(0.0–78.0)	131	7.3	(2.4–13.0)	(0.0–39.8)
**MHI *S*. *japonicum*** **	1	3.1	(2.7–3.6)	N/A	105	*0.2	(0.2–0.3)	(0.0–4.7)	1	3.1	(2.7–3.6)	N/A	105	*0.2	(0.2–0.3)	(0.0–3.3)

Preschool-age children (1–5 years old), school-age children (6–18 years old), children (1–18 years old), adults (more than 18 years old), general population (any age), overall (includes all prevalence estimates), PC—preventive chemotherapy, N—number of prevalence estimates, Prev—prevalence, CI—confidence interval, MHI—moderate to heavy intensity

** significantly different between pre- and post-PC studies, N/A not applicable, ^~^ significant difference only in “all diagnostic tests”

# significant difference only in “Kato Katz/Kato thick”

*- I^2^ below 90

The provinces of Bohol and Leyte had enough prevalence estimates for schistosomiasis both for prior to and after preventive chemotherapy initiation to allow meta-analysis. Pooled prevalence of schistosomiasis in Bohol after preventive chemotherapy initiation (0.04%) was significantly lower than the prevalence prior to preventive chemotherapy initiation (0.93%) (Table 6). Pooled prevalence of schistosomiasis in Leyte after preventive chemotherapy initiation was 12.8%, while the prevalence prior to preventive chemotherapy initiation was 35.3% (Table 6).

**Table 6:** Meta-analyses of prevalence of *S*. *japonicum* by preventive chemotherapy status and population group in Bohol and Leyte provinces (1954–2015).

INFECTION/GROUP	ALL DIAGNOSTIC TESTS							
	PRE-PC INITIATION				POST-PC INITIATION			
	N	Prev	95% CI	Range	N	Prev	95% CI	Range
**Bohol**								
Overall	20	0.9	(0.2–1.8)	(0.1–22.2)	2	0.04	(0.0–0.2)	(0.0–16.8)
**Leyte**								
School-age children**	36	37.7	(31.7–43.8)	(0.6–78.0)	2	*0.8	(0.3–1.7)	(0.5–78.0)
Overall	47	35.3	(30.1–40.7)	(0.6–78.0)	6	12.8	(0.0–38.3)	(0.5–79.3)

School-age children (6–18 years old), overall (includes all prevalence estimates), PC—preventive chemotherapy, N—number of prevalence estimates, Prev—prevalence, CI—confidence interval

** significantly different between pre- and post-PC studies

*- I^2^ below 90

When meta-analysis was restricted to only include prevalence estimates obtained within five years before or after preventive chemotherapy initiation, no reduction in prevalence of schistosomiasis was observed. Prevalence prior to preventive chemotherapy initiation from 7 prevalence estimates was at 9.4% (95% CI 0.0–23.8%) with I^2^ = 99.3, while prevalence after preventive chemotherapy initiation from 21 prevalence estimates was 22.5% (95% CI 5.8%-41.8%) with I^2^ = 99.7.

Three studies reported risk factors for schistosomiasis.[36–38] Being female, being wealthy(36), and not working on a farm were associated with a significantly lower risk of schistosomiasis. On the other hand, being 15–40 years old, being a student, being employed particularly in farming and fishing, having only a high school/vocational degree or less, having contact with water in rivers, being poor [36], having STH infection, having at least one sibling with schistosomiasis, and having a family enrolled in a conditional cash transfer program were associated with significantly higher risk of schistosomiasis (S6 Table).

### Lymphatic filariasis

#### Descriptive analysis

Nineteen studies (75 prevalence estimates) reported prevalence of lymphatic filariasis (see S7 Table). All were from studies published in peer-reviewed journals and were conducted in the general population prior to preventive chemotherapy initiation. We were unable to access Transmission Assessment Survey data from the national program or any other unpublished data. The most recent prevalence estimate was in 1994, while the earliest was in 1956.The studies utilised microscopy of blood samples, except for one that used PCR.[39] Most studies utilised nocturnal blood microscopy using Giemsa stain (61 prevalence estimates, 83.56%), while 11 prevalence estimates (15.07%) used variations of blood microscopy such as the use of films and filters.

**Fig 4:**
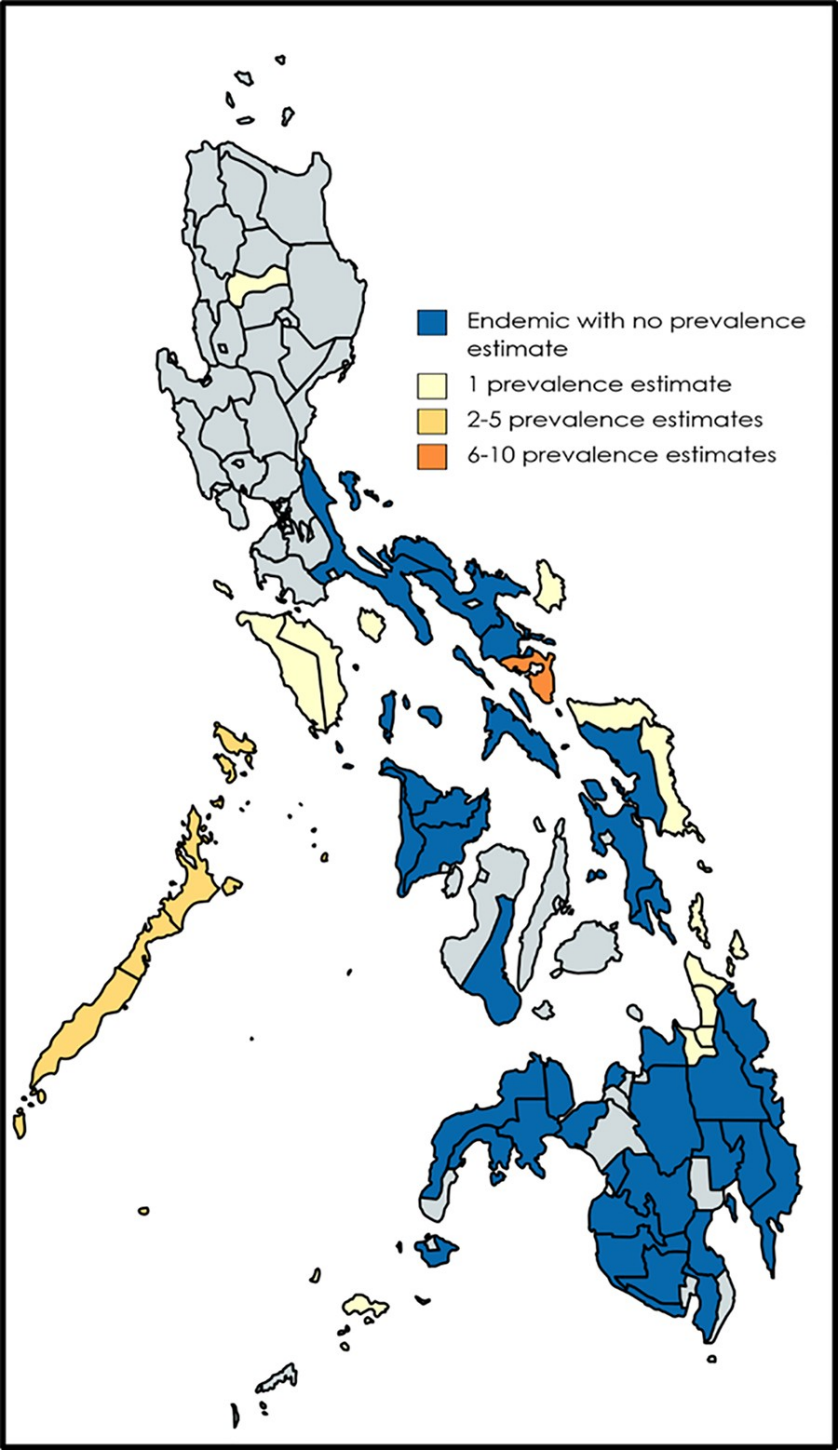
Sites of lymphatic filariasis prevalence estimates. Created using MapChart (https://mapchart.net/index.html.

Excluding a nationwide study that contributed 49 prevalence estimates [40], 12 out of 46 endemic provinces were represented. Sorsogon (10 prevalence estimates, 13.70%) and Palawan (4 prevalence estimates, 5.48%) were the most common study sites (Fig 4).

#### Meta-analysis

Pooled prevalence of lymphatic filariasis prior to preventive chemotherapy initiation was 3.2% (1.1–5.7%) ranging from zero to 64.3%. When the only prevalence estimate which did not use blood microscopy (8 positive out of 54 examined using PCR in Sorsogon province in 1994) was excluded, the pooled prevalence remained the same.

No data on prevalence of lymphatic filariasis data after preventive chemotherapy initiation were available for meta-analysis. However, the national program data from the Department of Health currently classifies only two provinces, namely Zamboanga del Norte and Sultan Kudarat, as endemic and still implementing MDA due to prevalence above 1% obtained through rapid immunochromatographic test (see S8 Table for classification of lymphatic filariasis-endemic provinces). The prevalence of lymphatic filariasis in these two provinces obtained through nocturnal blood microscopy in 1963 were 0.8% and 2.1%, respectively.

## Discussion

Despite programs implementing preventive chemotherapy for the control of helminth-related infections in the Philippines for over a decade, our report appears to be the first comprehensive assessment of program impact.

In the Philippines, preventive chemotherapy for STH is implemented in all provinces and highly urbanised cities twice a year (every January and July) among school-age children and preschool children.[41] In our analysis, prevalence of STH infections among children was significantly lower in studies conducted after the initiation of preventive chemotherapy, although these were still at levels where preventive chemotherapy is recommended.[42] The overall prevalence of moderate-heavy intensity *A*. *lumbricoides* and *T*. *trichiura* after preventive chemotherapy initiation were also significantly lower, although these were still beyond the WHO target of less than 2%.[42] The prevalence of moderate-heavy intensity hookworm infection was below the 2% target.[27]

High STH burden persists in some provinces as seen in the wide prevalence ranges despite biannual preventive chemotherapy targeting children since 2006.[10] A major reason may be the decentralised health system where local governments are responsible for delivering health services. This may result in variable program implementation across local government units. For instance, some local government units may use a house-to-house approach while others may use community assemblies to implement MDA. There may also be differences in social mobilisation activities, as well as in the implementation of other interventions thought to have an impact on STH prevalence such as WASH and health promotion. Advocacy with local government leaders and enhancing the capacity of health service providers to help improve service delivery and ensure more accountability may help address this challenge.[43] Another potential reason for limited impact is the coverage of preventive chemotherapy. Indeed, it has been varying from a low of 15.1% in 2013 to a high of 76.4% in 2016, barely meeting the at least 75% target of the WHO. In 2019, 59% of the over 45 million requiring preventive chemotherapy received treatment. [42, 44, 45]

In the Philippines, preventive chemotherapy for schistosomiasis is implemented in 190 municipalities and 28 provinces once a year (every January) in all residents ages 5 and above of endemic barangays.[41, 46] Preventive chemotherapy coverage has been varying from a low of 14.83% in 2014 to a high of 64.82% in 2017. In 2019, 55.8% of the 2.7 million requiring preventive chemotherapy received treatment. In our analysis, prevalence of schistosomiasis was significantly lower after initiation of MDA but only in school-age children, while the program distributes praziquantel to everyone above five years old in known endemic villages. This may be due to praziquantel being co-administered with albendazole in schools [41], which may increase coverage of this age group.[44, 47] Nevertheless, schistosomiasis could be considered as being eliminated as a public health problem, defined as prevalence of heavy intensity schistosomiasis of 1%.[48] Validating this milestone, however, is needed following a more rigorous and systematic assessment in sentinel sites.[49] Despite the low prevalence of schistosomiasis and of moderate-heavy intensity schistosomiasis at 0.2%, the range of prevalence estimates varies widely which may be due to the highly focal distribution of schistosomiasis. Thus, country-level prevalence may be less useful in understanding the true burden of schistosomiasis.

While preventive chemotherapy as a key strategy of national programs for STH infections and schistosomiasis had positive impact, it may be appropriate to investigate areas for improvement and consider how the observed prevalence reductions can be extended to other age groups and other high-risk areas. Interrupting transmission of STH infections is thought to require shifting from school based deworming to MDA [31, 50, 51], given that school-based preventive chemotherapy is unlikely to impact community-wide STH transmission.[52–54] Indeed, in our meta-analysis, prevalence of STH infections among adults and general population were similar before and after the initiation of preventive chemotherapy. MDA has been used for transmission elimination of other NTDs, including lymphatic filariasis, trachoma, and onchocerciasis.[55–58]

In the Philippines, preventive chemotherapy for lymphatic filariasis is implemented in two provinces (Zamboanga del Norte and Sultan Kudarat) once a year (every July) among all residents ages two and above. Preventive chemotherapy coverage has been generally increasing from a low of 0.92% in 2000 to a high of 79.47% in 2013. In 2019, 72.3% of the 3.6 million requiring preventive chemotherapy received treatment.[44, 59]

The study was unable to pool post-preventive chemotherapy initiation prevalence of lymphatic filariasis due to the lack of published studies, and inability to access data from the transmission assessment surveys.[60] Nonetheless, more than 20 years of preventive chemotherapy seem to have reduced the prevalence of lymphatic filariasis from 3.22% in all provinces, based on the meta-analysis, to only two out of 81 provinces having prevalence of more than 1% and still implementing MDA.

Majority of the studies included were unable to employ appropriate statistical analysis, while a considerable number did not report the sampling procedure and the sample size. Most of these are older studies which may be partly due to the reporting standards for prevalence studies being recently developments. The Joanna Briggs Institute Appraisal Checklist for Studies Reporting Prevalence Data, for instance, was only published in 2015. The authors tried to address these limitations in the included studies, for instance by extracting the raw data from the studies and performing separate statistical analysis.

A limitation of the study is that we did not account for other factors which could also contribute to changes in infection prevalence. Examples include broad changes such as economic development, or more specific interventions including WASH. Neither did the study account for the possibility of suboptimal efficacy of preventive chemotherapy drugs due to possible anthelmintics resistance, which is recommended to be regularly monitored.[61, 62] Additionally, we may not have accessed all grey literature reports on STH infections, schistosomiasis, and lymphatic filariasis prevalence. For instance, there is a lack of prevalence estimates from 14 provinces designated as schistosomiasis-endemic, with most prevalence estimates in Bohol and Leyte. We were also unable to obtain grey literature for lymphatic filariasis. Another limitation is that the “post-preventive chemotherapy initiation” prevalence estimates reflect varying degrees of reinfection because the time elapsed from a given round of preventive chemotherapy to the period of data collection is often not indicated for these prevalence estimates. The highly heterogenous results demonstrated by the high I^2^ values, mostly above 90, may be due to variability in sampling procedures, diagnostic tests, study sites, and years of data collection. We tried to address the highly heterogenous results by using the inverse variance heterogeneity model. The high I^2^ values, which were comparable to the I^2^ values observed in other meta-analysis of prevalence [63–66], is expected in any broad-scope meta-analysis.[67]

In conclusion, while previous publications have utilised meta-analysis to estimate country-level prevalence of STH infections in Africa [65, 68] and South America [69], schistosomiasis in Africa [70, 71] and in South America [22], and lymphatic filariasis in Asia [72], this is the first meta-analysis comparing the prevalence of STH infections, schistosomiasis, and lymphatic filariasis before and after the initiation of large-scale mass treatment programs. This study showed that the burden of STH infections and schistosomiasis were significantly lower in children in studies conducted following the implementation of preventive chemotherapy as part of the national programs. Additionally, it appears that lymphatic filariasis burden has decreased since the implementation of preventive chemotherapy, with all but two provinces having eliminated lymphatic filariasis as a public health problem. Interrupting STH infections and schistosomiasis transmission will require considering MDA, using more accurate diagnostics, and access to improved WASH.

## Supporting information

S1 PRISMA Checklist PRISMA checklist. (DOCX)

S1 Text Search Strings. (DOCX)

S1 Table Risk of bias assessment using the Joanna Briggs Institute’s Critical Appraisal Checklist for Prevalence Studies. (DOCX)

S2 Table Studies which reported STH and schistosomiasis prevalence. (DOCX)

S3 Table Studies which reported STH prevalence only. (DOCX)

S4 Table Risk factors for STH infections. (DOCX)

S5 Table Studies which reported schistosomiasis prevalence only. (DOCX)

S6 Table Risk factors for schistosomiasis. (DOCX)

S7 Table Studies which reported lymphatic filariasis prevalence only. (DOCX)

S8 Table Categories of Lymphatic Filariasis-Endemic Provinces. (DOCX)
