# Supplementary material for: Prevalence of soil-transmitted helminth infections, schistosomiasis, and lymphatic filariasis before and after preventive chemotherapy initiation in the Philippines: A systematic review and meta-analysis
Source: PLoS Negl Trop Dis. 2021 Dec 20;15(12):e0010026. doi: 10.1371/journal.pntd.0010026 (PMC8722724; doi:10.1371/journal.pntd.0010026)
Supplement: S1 Text — (DOCX) [file pntd.0010026.s002.docx]

**S1 Text. Search Strings**

**MEDLINE**

((soil-transmitted helminth or ancylostoma duodenale or ancylostomiasis or *Ascaris* or ascariasis or geohelminths or helminthiasis or helminth infection or helminth or hookworm or hookworm infections or necator or necatoriasis or necator americanus or nematode or nematode infections or roundworm or strongyloides or strongyloides stercoralis or strongyloidiasis or *Trichuris* or trichuriasis or whipworm or schistosomiasis or bilharzia or blood fluke or Katayama fever or schistosomiasis japonicum or *Schistosoma japonicum* or lymphatic filariasis or elephantiasis or filaria or *Wuchereria bancrofti* or *Brugia timori* or *Brugia malayi* or microfilaria or neglected tropical disease) and Philippines and (prevalence or risk factors or deworming or health education or health promotion or mass drug administration or mosquito control or insecticide-treated nets or preventive chemotherapy or selective treatment or snail control or vector control or water, sanitation, hygiene or albendazole or mebendazole or praziquantel or diethylcarbamazine)).mp. [mp=title, abstract, original title, name of substance word, subject heading word, floating sub-heading word, keyword heading word, organism supplementary concept word, protocol supplementary concept word, rare disease supplementary concept word, unique identifier, synonyms]

**EMBASE**

((soil-transmitted helminth or ancylostoma duodenale or ancylostomiasis or *Ascaris* or ascariasis or geohelminths or helminthiasis or helminth infection or helminth or hookworm or hookworm infections or necator or necatoriasis or necator americanus or nematode or nematode infections or roundworm or strongyloides or strongyloides stercoralis or strongyloidiasis or *Trichuris* or trichuriasis or whipworm or schistosomiasis or bilharzia or blood fluke or Katayama fever or schistosomiasis japonicum or *Schistosoma japonicum* or lymphatic filariasis or elephantiasis or filaria or *Wuchereria bancrofti* or *Brugia timori* or *Brugia malayi* or microfilaria or neglected tropical disease) and Philippines and (prevalence or risk factors or deworming or health education or health promotion or mass drug administration or mosquito control or insecticide-treated nets or preventive chemotherapy or selective treatment or snail control or vector control or water, sanitation, hygiene or albendazole or mebendazole or praziquantel or diethylcarbamazine)).mp. [mp=title, abstract, heading word, drug trade name, original title, device manufacturer, drug manufacturer, device trade name, keyword, floating subheading word, candidate term word]

**Global Health**

((soil-transmitted helminth or ancylostoma duodenale or ancylostomiasis or *Ascaris* or ascariasis or geohelminths or helminthiasis or helminth infection or helminth or hookworm or hookworm infections or necator or necatoriasis or necator americanus or nematode or nematode infections or roundworm or strongyloides or strongyloides stercoralis or strongyloidiasis or *Trichuris* or trichuriasis or whipworm or schistosomiasis or bilharzia or blood fluke or Katayama fever or schistosomiasis japonicum or *Schistosoma japonicum* or lymphatic filariasis or elephantiasis or filaria or *Wuchereria bancrofti* or *Brugia timori* or *Brugia malayi* or microfilaria or neglected tropical disease) and Philippines and (prevalence or risk factors or deworming or health education or health promotion or mass drug administration or mosquito control or insecticide-treated nets or preventive chemotherapy or selective treatment or snail control or vector control or water, sanitation, hygiene or albendazole or mebendazole or praziquantel or diethylcarbamazine)).mp. [mp=abstract, title, original title, broad terms, heading words, identifiers, cabicodes]

**Scopus**

( ( *soiltransmitted*  AND  *helminth* )  OR  ( *ancylostoma*  AND  *duodenale* )  OR  *ancylostomiasis*  OR  *Ascaris*  OR  *ascariasis*  OR  *geohelminths*  OR  *helminthiasis*  OR  ( *helminth*  AND  *infection* )  OR  *helminth*  OR  *hookworm*  OR  ( *hookworm*  AND  *infections* )  OR  *necator*  OR  *necatoriasis*  OR  ( *necator*  AND  *americanus* )  OR  *nematode*  OR  ( *nematode*  AND  *infections* )  OR  *roundworm*  OR  *strongyloides*  OR  ( *strongyloides*  AND  *stercoralis* )  OR  *strongyloidiasis*  OR  *Trichuris*  OR  *trichuriasis*  OR  *whipworm*  OR  *schistosomiasis*  OR  *bilharzia*  OR  ( *blood*  AND  *fluke* )  OR  ( *katayama*  AND  *fever* )  OR  ( *schistosomiasis*  AND  *japonicum* )  OR  ( *Schistosoma*  AND  *japonicum* )  OR  ( *lymphatic*  AND  *filariasis* )  OR  *elephantiasis*  OR  *filaria*  OR  ( *wuchereria*  AND  *bancrofti* )  OR  ( *brugia*  AND  *timori* )  OR  ( *brugia*  AND  *malayi* )  OR  *microfilaria*  OR  ( *neglected*  AND  *tropical*  AND  *disease* ) )  AND  ( *philippines* )  AND  ( *prevalence*  OR  ( *risk*  AND  *factors* )  OR  *deworming*  OR  ( *health*  AND  *education* )  OR  ( *health*  AND  *promotion* )  OR  ( *mass*  AND  *drug*  AND  *administration* )  OR  ( *mosquito*  AND  *control* )  OR  ( *insecticide-treated*  AND  *nets* )  OR  ( *preventive*  AND  *chemotherapy* )  OR  ( *selective*  AND  *treatment* )  OR  ( *snail*  AND  *control* )  OR  ( *vector*  AND  *control* )  OR  ( *water,*  AND  *sanitation,*  AND  *hygiene* )  OR  *albendazole*  OR  *mebendazole*  OR  *praziquantel*  OR  *diethylcarbamazine* )  AND  ( LIMIT-TO ( PUBYEAR ,  *2020* )  OR  LIMIT-TO ( PUBYEAR ,  *2019* ) )  AND  ( LIMIT-TO ( DOCTYPE ,  *"ar"* ) )  AND  ( LIMIT-TO ( SUBJAREA ,  *"MEDI"* ) )  AND  ( LIMIT-TO ( LANGUAGE ,  *"English"* ) )

**EMCARE**

((soil-transmitted helminth or ancylostoma duodenale or ancylostomiasis or *Ascaris* or ascariasis or geohelminths or helminthiasis or helminth infection or helminth or hookworm or hookworm infections or necator or necatoriasis or necator americanus or nematode or nematode infections or roundworm or strongyloides or strongyloides stercoralis or strongyloidiasis or *Trichuris* or trichuriasis or whipworm or schistosomiasis or bilharzia or blood fluke or Katayama fever or schistosomiasis japonicum or *Schistosoma japonicum* or lymphatic filariasis or elephantiasis or filaria or *Wuchereria bancrofti* or *Brugia timori* or *Brugia malayi* or microfilaria or neglected tropical disease) and Philippines).mp. [mp=title, abstract, heading word, drug trade name, original title, device manufacturer, drug manufacturer, device trade name, keyword]

**DOAJ**

((soil-transmitted helminth) OR (ancylostoma duodenale) OR ancylostomiasis OR *Ascaris* OR ascariasis OR geohelminths OR helminthiasis OR (helminth infection) OR helminth OR hookworm OR (hookworm infections) OR necator OR necatoriasis OR (necator americanus) OR nematode OR (nematode infections) OR roundworm OR strongyloides OR (strongyloides stercoralis) OR strongyloidiasis OR *Trichuris* OR trichuriasis OR whipworm OR schistosomiasis OR bilharzia OR (blood fluke) OR (Katayama fever) OR (schistosomiasis japonicum) OR (*Schistosoma japonicum*) OR (lymphatic filariasis) OR elephantiasis OR filaria OR (*Wuchereria bancrofti*) OR (*Brugia timori*) OR (*Brugia malayi*) OR microfilaria OR (neglected tropical disease)) AND (Philippines)

**HERDIN**

((soil-transmitted helminth) OR (ancylostoma duodenale) OR ancylostomiasis OR *Ascaris* OR ascariasis OR geohelminths OR helminthiasis OR (helminth infection) OR helminth OR hookworm OR (hookworm infections) OR necator OR necatoriasis OR (necator americanus) OR nematode OR (nematode infections) OR roundworm OR strongyloides OR (strongyloides stercoralis) OR strongyloidiasis OR *Trichuris* OR trichuriasis OR whipworm OR schistosomiasis OR bilharzia OR (blood fluke) OR (Katayama fever) OR (schistosomiasis japonicum) OR (*Schistosoma japonicum*) OR (lymphatic filariasis) OR elephantiasis OR filaria OR (*Wuchereria bancrofti*) OR (*Brugia timori*) OR (*Brugia malayi*) OR microfilaria OR (neglected tropical disease)) fulltext:request-based publication-date:2019-2020
