# Supplementary material for: Prevalence of soil-transmitted helminth infections, schistosomiasis, and lymphatic filariasis before and after preventive chemotherapy initiation in the Philippines: A systematic review and meta-analysis
Source: PLoS Negl Trop Dis. 2021 Dec 20;15(12):e0010026. doi: 10.1371/journal.pntd.0010026 (PMC8722724; doi:10.1371/journal.pntd.0010026)
Supplement: S1 Table — (DOCX) [file pntd.0010026.s003.docx]

**S1 Table. Risk of bias assessment using the Joanna Briggs Institute’s Critical Appraisal Checklist for Prevalence Studies.**

| **Reference** | **Was the sample frame appropriate to address the target population** | **Were study participants sampled in an appropriate way?** | **Was the sample size adequate?** | **Were the study subjects and the setting described in detail?** | **Was the data analysis conducted with sufficient coverage of the identified sample?** | **Were valid method used for the identification of the condition?** | **Was the condition measured in a standard, reliable way for all participations?** | **Was there appropriate statistical analysis?** | **Was the response rate adequate? If not, was the low response rate managed appropriately?** |
| --- | --- | --- | --- | --- | --- | --- | --- | --- | --- |
| [1] | Yes | Yes | Yes | Yes | Yes | Yes | Yes | Yes | Yes |
| [2] | Yes | Unclear | Yes | Yes | Yes | Yes | Yes | Yes | Yes |
| [3] | Yes | Yes | Yes | Yes | Yes | Yes | Yes | Yes | Yes |
| [4] | Yes | Yes | Unclear | Yes | Yes | Yes | Yes | Yes | Yes |
| [5] | Yes | Yes | Yes | Yes | Yes | Yes | Yes | No | Yes |
| [6] | Yes | Yes | Yes | Yes | Yes | Yes | Yes | Yes | Yes |
| [7] | Yes | Yes | Yes | Yes | Yes | Yes | Yes | Yes | Yes |
| [8] | Yes | Yes | Yes | Yes | Yes | Yes | Yes | Yes | Yes |
| [9] | Unclear | Unclear | Unclear | Unclear | Unclear | Yes | Yes | Yes | Unclear |
| [10] | Yes | Yes | Yes | Yes | Yes | Yes | Yes | Yes | Yes |
| [11] | Yes | Yes | Yes | Yes | No | Yes | Yes | Yes | No |
| [12] | Unclear | Unclear | Unclear | Yes | Unclear | Yes | Unclear | Yes | Unclear |
| [13] | Unclear | Unclear | Unclear | Yes | Unclear | Yes | Unclear | Yes | Unclear |
| [14] | Yes | Unclear | Unclear | Yes | No | Yes | Yes | Yes | No |
| [15] | Yes | No | Yes | Yes | Yes | Yes | Yes | Yes | Unclear |
| [16] | Yes | No | No | Yes | Yes | Yes | Yes | No | Unclear |
| [17] | Yes | Yes | Yes | Yes | Yes | Yes | Yes | Yes | Yes |
| [18] | Yes | No | No | Yes | Yes | Yes | Yes | Yes | Unclear |
| [19] | Yes | Unclear | Yes | Yes | Yes | Yes | Yes | Yes | Unclear |
| [20] | Unclear | Unclear | Unclear | Yes | No | Yes | Unclear | Yes | No |
| [21] | Unclear | Unclear | Unclear | Yes | Yes | Yes | Unclear | Yes | Unclear |
| [22] | Yes | Yes | Yes | Yes | Yes | Yes | Yes | Yes | Unclear |
| [23] | No | No | Yes | Yes | Yes | Yes | Yes | Yes | Yes |
| [24] | Yes | No | No | Yes | Yes | Yes | Yes | Yes | Unclear |
| [25] | Yes | Unclear | No | Yes | Yes | Yes | Yes | Yes | Unclear |
| [26] | Yes | Unclear | Yes | Yes | Yes | Unclear | Unclear | Yes | Unclear |
| [27] | Yes | Yes | Yes | Yes | Yes | Yes | Yes | Yes | Yes |
| [28] | Yes | Unclear | Unclear | Yes | Yes | Yes | Unclear | Yes | Unclear |
| [29] | Yes | Unclear | Unclear | Yes | Yes | Unclear | Unclear | Yes | Unclear |
| [30] | Yes | Yes | Yes | Yes | Yes | Yes | Yes | Yes | Yes |
| [31] | Yes | No | Unclear | Yes | Yes | Yes | Yes | Yes | Unclear |
| [32] | Yes | Yes | Yes | Yes | Yes | Yes | Yes | No | Yes |
| [33] | Yes | No | No | Yes | Yes | Yes | Yes | Yes | Unclear |
| [34] | Yes | Unclear | Yes | Yes | Unclear | Yes | Unclear | Yes | Unclear |
| [35] | Yes | Yes | Yes | Yes | Yes | Yes | Yes | No | Yes |
| [36] | Yes | Yes | Yes | Yes | Yes | Yes | Yes | Yes | Yes |
| [37] | No | Yes | Yes | Yes | Unclear | Yes | Unclear | Yes | Unclear |
| [38] | Yes | Yes | Yes | Yes | Yes | Yes | Yes | Yes | Yes |
| [39] | Yes | Yes | Yes | Yes | Yes | No | Yes | Yes | Yes |
| [40] | Yes | No | Yes | Yes | Yes | Yes | Yes | Yes | Yes |
| [41] | Yes | Yes | Yes | Yes | Yes | Yes | Yes | Yes | Yes |
| [42] | Yes | Yes | Yes | Yes | Yes | Yes | Yes | Yes | Yes |
| [43] | Yes | Yes | Yes | Yes | Yes | Yes | Yes | Yes | Yes |
| [44] | No | Yes | Yes | Yes | Yes | Yes | Yes | Yes | Yes |
| [45] | Yes | No | Unclear | Yes | Unclear | Yes | Yes | No | Unclear |
| [46] | Yes | Yes | Yes | Yes | Yes | Yes | Yes | No | Yes |
| [47] | Unclear | Unclear | Unclear | No | Unclear | Yes | Yes | No | Unclear |
| [48] | Unclear | Unclear | Yes | No | Yes | Yes | Yes | No | Unclear |
| [49] | Unclear | Unclear | Unclear | No | Yes | Yes | Yes | No | Unclear |
| [50] | Unclear | Unclear | Unclear | Yes | Yes | Yes | Yes | No | Unclear |
| [51] | Unclear | Yes | Unclear | No | Unclear | Yes |  | Yes | Unclear |
| [52] | Unclear | Unclear | Unclear | No | Yes | Yes | Yes | Yes | No |
| [53] | Yes | Yes | Yes | Yes | Yes | Yes | Yes | No | Yes |
| [54] | Yes | Yes | Yes | Yes | Yes | Yes | Yes | No |  |
| [55] | Yes | No | Yes | No | Yes | Yes | Yes | No | Yes |
| [56] | No | Yes | Yes | Yes | Yes | Yes | Yes | No | Yes |
| [57] | Yes | Unclear | Unclear | Yes | Yes | Yes | Yes | Yes | Unclear |
| [58] | Yes | Yes | Yes | Yes | Yes | No | Yes | No | Yes |
| [59] | Unclear | No | Unclear | No | Yes | Yes | Yes | No | Yes |
| [60] | Yes | Yes | Yes | Yes | Yes | Yes | Yes | No | Yes |
| [61] | Yes | Yes | Yes | Yes | Yes | Yes | Yes | No | Yes |
| [62] | Yes | Unclear | Unclear | Yes | Yes | Yes | Yes | No | Yes |
| [63] | Yes | Yes | Yes | Yes | Yes | No | Yes | No | Unclear |
| [64] | No | Yes | Yes | Yes | Yes | Yes | Yes | No | Yes |
| [65] | Yes | Yes | Unclear | Yes | No | Yes | Yes | No | No |
| [66] | Yes | Yes | Yes | Yes | Yes | Yes | Yes | No | Yes |
| [67] | No | Yes | Yes | Yes | Yes | Yes | Yes | No | Yes |
| [68] | Yes | Yes | No | Yes | Yes | Yes | Yes | No | Yes |
| [69] | Yes | Unclear | Unclear | Yes | Yes | No | Unclear | No | Yes |
| [70] | Yes | Unclear | Yes | Yes | Yes | Yes | Yes | No | Yes |
| [71] | Yes | Unclear | Unclear | Yes | Unclear | Yes | Yes | No | Unclear |
| [72] | No | Yes | No | Yes | Yes | Yes | Yes | No | Yes |
| [73] | No | Yes | Unclear | Yes | Unclear | Yes | Yes | No | Yes |
| [74] | Yes | Yes | Yes | Yes | Yes | Yes | Yes | No | Yes |
| [75] | No | No | Unclear | Yes | Yes | Yes | Yes | No | Yes |
| [76] | No | No | Unclear | Yes | Yes | Yes | Yes | No | Yes |
| [77] | Yes | Yes | Yes | Yes | Yes | Yes | Yes | Yes | Yes |
| [78] | Yes | Yes | Yes | Yes | Yes | Yes | Yes | No | Yes |
| [79] | Unclear | Unclear | Unclear | Yes | Yes | No | Yes | No | Yes |
| [80] | Yes | No | Unclear | No | Unclear | Yes | Yes | No | Yes |
| [81] | No | No | Unclear | Yes | Unclear | Yes | Yes | No | Yes |
| [82] | Unclear | Unclear | Unclear | Yes | Yes | Yes | Yes | No | Yes |
| [83] | Yes | Yes | Unclear | Yes | Yes | Yes | Yes | No | Yes |
| [84] | Yes | Yes | Yes | Yes | Yes | Yes | Yes | Yes | Yes |
| [85] | Yes | No | Unclear | Yes | Yes | Yes | Yes | No | Yes |
| [86] | No | Yes | Unclear | Yes | No | Yes | Yes | No | No |
| [87] | Yes | Yes | Yes | Yes | No | Yes | Yes | No | Yes |
| [88] | Yes | Yes | Yes | No | Yes | Yes | Yes | No | Yes |
| [89] | No | Unclear | Unclear | No | Yes | Yes | Yes | No | Yes |
| [90] | No | Unclear | Unclear | No | Yes | Yes | Yes | No | Yes |
| [91] | Yes | Yes | Yes | Yes | Yes | Yes | Yes | No | Yes |
| [92] | Yes | Yes | Yes | Yes | Yes | Yes | Yes | No | Yes |
| [93] | Yes | Yes | Unclear | Yes | Yes | Yes | Yes | No | Yes |
| [94] | Yes | Yes | Yes | Yes | Yes | Yes | Yes | No | Yes |
| [95] | Yes | Yes | Unclear | Yes | Yes | Yes | Yes | No | Yes |
| [96] | Yes | No | Unclear | Yes | Yes | Yes | Yes | No | Yes |
| [97] | Unclear | Unclear | Unclear | Yes | Yes | Yes | Yes | No | Yes |
| [98] | No | Unclear | Unclear | Yes | Yes | Yes | Yes | No | Yes |
| [99] | Yes | Unclear | Yes | Yes | Unclear | Yes | Yes | No | Yes |
| [100] | No | Unclear | Unclear | Yes | Yes | Yes | Yes | No | Yes |
| [101] | No | No | Unclear | Yes | Unclear | Yes | Yes | No | Yes |
| [102] | No | Unclear | Unclear | Yes | Unclear | Yes | Yes | No | Yes |
| [103] | No | Unclear | Unclear | Yes | Unclear | Yes | Yes | No | Yes |
| [104] | No | Unclear | Unclear | Yes | Unclear | No | Unclear | No | Yes |
| [105] | No | Unclear | Unclear | Yes | Unclear | Yes | Yes | No | Yes |
| [106] | No | Unclear | Unclear | Yes | Unclear | Yes | Yes | No | Yes |
| [107] | Yes | Yes | Yes | Yes | Yes | No | No | No | Yes |

**References**

1. Labana R, Romero VA, Guinto AM, Caril AN, Untalan KD, Reboa AJC, et al. Prevalence and intensity of soil-transmitted helminth infections among school-age children in the Cagayan Valley, the Philippines. Asian Pac J Trop Med. 2021;14(3):113-21.

2. Aula OP, McManus DP, Weerakoon KG, Olveda R, Ross AG, Rogers MJ, et al. Molecular identification of Ancylostoma ceylanicum in the Philippines. Parasitology. 2020;147(14):1718-22.

3. delos Trinos JPCR, Belizario VY, Sison OT, Erasmo JN, Te MJ, Modequillo MC. Child development center-based sentinel surveillance of soil-transmitted helminthiases in preschool-age children in selected local government units in the Philippines. Acta Tropica. 2019;194:100-5.

4. Cai PF, Weerakoon KG, Mu Y, Olveda RM, Ross AG, Olveda DU, et al. Comparison of Kato Katz, antibody-based ELISA and droplet digital PCR diagnosis of schistosomiasis japonica: Lessons learnt from a setting of low infection intensity. Plos Neglect Trop Dis. 2019;13(3):17.

5. Palasi L, Galit, Tangcalagan, Bernal, Eugenio, 2018. Community-Based Survey on the Prevalence of Soil-Transmitted Helminths, Schistosomiasis, and Other Intestinal Parasitic Infections among Preschool Age Children, Adolescents and Adults [DRAFT]. 2018.

6. Weerakoon KG, Gordon CA, Williams GM, Cai P, Gobert GN, Olveda RM, et al. Co-parasitism of intestinal protozoa and Schistosoma japonicum in a rural community in the Philippines. Infectious Diseases of Poverty. 2018;7 (1) (no pagination)(121):121.

7. Belizario V, Destura R, Gabunada RR, Petronio-Santos JA, de la Tonga A, Amarillo ML, et al. Evaluation of fecal and serological tests for the diagnosis of schistosomiasis in selected near-elimination and endemic areas in the philippines. Southeast Asian Journal of Tropical Medicine and Public Health. 2018;49(2):198-207.

8. Belizario VY. War on Worms Lapu-Lapu City Follow-Up Parasitological Assessment. 2017.

9. Weerakoon KG, Gordon CA, Williams GM, Cai PF, Gobert GN, Olveda RM, et al. Droplet Digital PCR Diagnosis of Human Schistosomiasis: Parasite Cell-Free DNA Detection in Diverse Clinical Samples. J Infect Dis. 2017;216(12):1611-22.

10. Liwanag HJ, Uy J, Bataller R, Gatchalian JR, De La Calzada B, Uy JA, et al. Soil-transmitted helminthiasis and schistosomiasis in children of poor families in leyte, Philippines: Lessons for disease prevention and control. Journal of Tropical Pediatrics. 2017;63(5):335-45.

11. Mationg MLS, Gordon CA, Tallo VL, Olveda RM, Alday PP, Renosa MDC, et al. Status of soil-transmitted helminth infections in schoolchildren in Laguna Province, the Philippines: Determined by parasitological and molecular diagnostic techniques. Plos Neglect Trop Dis. 2017;11(11):16.

12. Yu W, Ross AG, Olveda RM, Harn DA, Li Y, Chy D, et al. Risk of human helminthiases: geospatial distribution and targeted control. International Journal of Infectious Diseases. 2017;55:131-8.

13. Ross AG, Olveda RM, McManus DP, Harn DA, Chy D, Li Y, et al. Risk factors for human helminthiases in rural Philippines. International Journal of Infectious Diseases. 2017;54:150-5.

14. Olveda DU, Inobaya M, Olveda RM, Vinluan ML, Ng SK, Weerakoon K, et al. Diagnosing schistosomiasis-induced liver morbidity: implications for global control. International Journal of Infectious Diseases. 2017;54:138-44.

15. Belizario VY, Ng JV, Amarillo MLE, delos Trinos JPCR, Reyes MR, Fudalan O. High burden of soil-transmitted helminthiases in preschool-age children in masbate: A decade of implementation of the integrated helminth control program in the Philippines. Southeast Asian Journal of Tropical Medicine and Public Health. 2016;47(4):667-79.

16. Belizario V, Jr., Bungay AA, Su GS, de Veyra C, Lacuna JD. Assessment of three schistosomiasis endemic areas using kato-katz technique and elisa antigen and antibody tests. Southeast Asian Journal of Tropical Medicine and Public Health. 2016;47(4):638-50.

17. RITM. National Survey on the Prevalence of Soil-Transmitted Helminths (STH), Schistosomiasis and other Intestinal Parasitic Infections among Public School Children in the Philippines [DRAFT]. 2016.

18. Belizario VY, Jr., Liwanag HJ, Naig JR, Chua PL, Madamba MI, Dahildahil RO. Parasitological and nutritional status of school-age and preschool-age children in four villages in Southern Leyte, Philippines: Lessons for monitoring the outcome of Community-Led Total Sanitation. Acta Tropica. 2015;141(Pt A):16-24.

19. Belizario VY, Jr., Erfe JM, Naig J, Chua P. Evidence of increasing risk of schistosomiasis among school-age children in municipality of Calatrava, Province of Negros Occidental, Philippines. Asian Pac J Trop Med. 2015;8(5):373-7.

20. Ross AG, Olveda RM, Chy D, Olveda DU, Li Y, Harn DA, et al. Can mass drug administration lead to the sustainable control of schistosomiasis? J Infect Dis. 2015;211(2):283-9.

21. Gordon CA, McManus DP, Acosta LP, Olveda RM, Williams GM, Ross AG, et al. Multiplex real-time PCR monitoring of intestinal helminths in humans reveals widespread polyparasitism in Northern Samar, the Philippines. International Journal for Parasitology. 2015;45(7):477-83.

22. Gordon CA, Acosta LP, Gobert GN, Olveda RM, Ross AG, Williams GM, et al. Real-time PCR Demonstrates High Prevalence of Schistosoma japonicum in the Philippines: Implications for Surveillance and Control. Plos Neglect Trop Dis. 2015;9(1):14.

23. Vicente Y. Belizario, Arlene G. Bertuso, Jolene Kristine G. Gatmaitan, Myra S. Mistica, Janis Ruth C. Gatchalian, Chua PLC. Food-Borne Helminth Infections in Selected Sites in the Philippines. Manila; 2015.

24. Belizario VJ TF, De Leon WU, Naig JRA. Improving Current Helminth Control Strategies: Lessons from a Baseline Prevalence Survey of Soil-Transmitted Helminth Infections in Adolescent Females and Pregnant Women in Selected Local Government Units in the Philippines. Acta Med Philipp. 2015;49(3): 5-11.

25. Belizario VY, Totanes FI, de Leon WU, Matias KM. School-based control of soil-transmitted helminthiasis in western Visayas, Philippines. The Southeast Asian journal of tropical medicine and public health. 2014;45(3):556-67.

26. Belizario Jr V, Chua PL, Liwanag HJ, Naig JR, Erfe JM. Soil-transmitted helminthiases in secondary school students in selected sites in two provinces in the Philippines: Policy implications. Journal of Tropical Pediatrics. 2014;60(4):303-7.

27. Ng JV, Vicente Y. Belizario J, Claveria FG. Determination of soil-transmitted helminth infection and its association with hemoglobin levels among Aeta schoolchildren of Katutubo Village in Planas, Porac, Pampanga Philippine Science Letters. 2014;7(1):73-80.

28. Horiuchi S, Paller VG, Uga S. Soil contamination by parasite eggs in rural village in the Philippines. Trop. 2013;30(3):495-503.

29. Sanza M, Totanes FI, Chua PL, Belizario VY, Jr. Monitoring the impact of a mebendazole mass drug administration initiative for soil-transmitted helminthiasis (STH) control in the Western Visayas Region of the Philippines from 2007 through 2011. Acta Tropica. 2013;127(2):112-7.

30. Monse B, Benzian H, Naliponguit E, Belizario V, Schratz A, Helderman WV. The Fit for School health outcome study - a longitudinal survey to assess health impacts of an integrated school health programme in the Philippines. BMC Public Health. 2013;13:10.

31. Cassion CC, Pingal ET, Maniago RB, Medina JRC, Belizario Jr VY. Schistosomiasis and soil-transmitted helminth infections in school children in the Lake Mainit area in Northeastern Mindanao: An opportunity for integrated helminth control in the school setting. Acta Med Philippina. 2013;47(3):4-10.

32. Abner O. Lawangen MCS, Danesto B. Anacio, Junelyn T. Tomin. Epidemiology of Soil-Transmitted Helminth Parasitism Among Schoolchildren in Tublay, Benguet. Tangkoyob: University of the Cordilleras Multidisciplinary Research Journal. 2012;6(1):15.

33. Belizario VY, Jr., Totanes FIG, Leon WUd, Lumampao YF, Ciro RNT. Soil-transmitted helminth and other intestinal parasitic infections among school children in indigenous people communities in Davao del Norte, Philippines. (Special Issue: The diagnostics and control of neglected tropical helminth diseases.). Acta Tropica. 2011;120(Supplement 1):S12-S8.

34. Belizario Jr VY, Plan AO, De Leon WU, Totañes FIG, Ciro RNT. Impact of a local government unit supported school-based initiative for control of intestinal helminth infections. Acta Med Philippina. 2011;45(2):18-23.

35. Batbatan CG. Prevalence of intestinal helminthic infections and assoiciated socio-cultural variables among schooling children of the public elementary schools of Maramag, Bukdinon. Central Mindanao University; 2009.

36. Belizario VY, Jr., Leon WUd, Lumampao YF, Anastacio MBM, Tai CMC. Sentinel surveillance of soil-transmitted helminthiasis in selected local government units in the Philippines. Asia Pacific Journal of Public Health. 2009;21(1):26-42.

37. Olson CL, Acosta LP, Hochberg NS, Olveda RM, Jiz M, McGarvey ST, et al. Anemia of inflammation is related to cognitive impairment among children in Leyte, The Philippines. Plos Neglect Trop Dis. 2009;3 (10) (no pagination)(e533).

38. Leonardo LR, Rivera P, Saniel O, Villacorte E, Crisostomo B, Hernandez L, et al. Prevalence survey of schistosomiasis in Mindanao and the Visayas, The Philippines. Parasitology International. 2008;57(3):246-51.

39. Tengco LW, Rayco-Solon P, Solon FS, Solon JA, Sarol JN. Determinants of Anemia among Preschool Children in the Philippines. J Am Coll Nutr. 2008;27(2):229-43.

40. Ezeamama AE, McGarvey ST, Acosta LP, Zierler S, Manalo DL, Wu HW, et al. The synergistic effect of concomitant schistosomiasis, hookworm, and trichuris infections on children's anemia burden. PLoS Negl Trop Dis. 2008;2(6):e245.

41. Tarafder MR, Balolong E, Carabin H, Belisle P, Tallo V, Joseph L, et al. A cross-sectional study of the prevalence of intensity of infection with Schistosoma japonicum in 50 irrigated and rain-fed villages in Samar Province, the Philippines. BMC Public Health. 2006;6:10.

42. Vicente Y. Belizario, Winifreda U. de Leon, Wambangco MAL, Esparar DG. Baseline assessment of intestinal parasitism in selected public elementary schools in Luzon, Visayas and Mindanao. Acta Med Philippina. 2005;39(2):11-21.

43. Vicente Y Belizario JMSV, Winifreda U de Leon, Donato G Esparar, Mark Phili Bugayong. Hookworm in the military: A parasitologic survey of military and para-military personnel in a Philippine military camp in Northern Luzon. Philippine Journal of Internal Medicine. 2005;43(4):169-74.

44. Ezeamama AE, Friedman JF, Acosta LP, Bellinger DC, Langdon GC, Manalo DL, et al. Helminth infection and cognitive impairment among Filipino children. Am J Trop Med Hyg. 2005;72(5):540-8.

45. Baldo ET, Belizario VY, De Leon WU, Kong HH, Chung DI. Infection status of intestinal parasites in children living in residential institutions in Metro Manila, the Philippines. The Korean journal of parasitology. 2004;42(2):67-70.

46. Ramirez BL, Hernandez L, Alberto FF, Collins M, Nfonsam V, Punsalan T, et al. Contrasting Wuchereria bancrofti microfilaria rates in two Mangyan-populated Philippine villages. Am J Trop Med Hyg. 2004;71(1):17-23.

47. Kim BJ, Ock MS, Chung DI, Yong TS, Lee KJ. The intestinal parasite infection status of inhabitants in the Roxas city, The Philippines. The Korean journal of parasitology. 2003;41(2):113-5.

48. Torres EP, Ramirez BL, Salazar F, Pasay MCJ, Alamares JG, Santiago ML, et al. Detection of bancroftian filariasis in human blood samples from Sorsogon province, the Philippines by polymerase chain reaction. Parasitol Res. 2001;87(8):677-9.

49. Lee KJ, Ahn YK, Yong TS. A small-scale survey of intestinal parasite infections among children and adolescents in Legaspi city, the Philippines. The Korean journal of parasitology. 2000;38(3):183-5.

50. Yamamoto R, Nagai N, Kawabata M, Ubas-De Leon W, Ninomiya R, Koizumi N. Effect of intestinal helminthiasis on nutritional status of schoolchildren. Southeast Asian Journal of Tropical Medicine and Public Health. 2000;31(4):755-61.

51. Kardorff R, Olveda RM, Acosta LP, Duebbelde UJ, Aligui GD, Alcorn NJ, et al. Hepatosplenic morbidity in schistosomiasis japonica: Evaluation with Doppler sonography. Am J Trop Med Hyg. 1999;60(6):954-9.

52. Ryoji Y, Nobuhiko N, Ubas-de Leon W, Naoko K. The relation between serum fatty acids and soil-transmitted helminthiasis in the Philippines. Southeast Asian Journal of Tropical Medicine and Public Health. 1997;28(2):329-34.

53. Yasuraoka K, Blas BL, Matsuda H, Irie Y, Nihei N, Ohmae H, et al. Approaches to the elimination of schistosomiasis on Bohol Island, Philippines. Japanese Journal of Parasitology. 1996;45(5):391-9.

54. Olveda RM, Daniel BL, Ramirez BDL, Aligui GDL, Acosta LP, Fevidal P, et al. Schistosomiasis japonica in the Philippines: the long-term impact of population-based chemotherapy on infection, transmission, and morbidity. J Infect Dis. 1996;174(1):163-72.

55. Go VM. Lymphatic filariasis in a recently described endemic area in Marinduque, Philippines. The Southeast Asian journal of tropical medicine and public health. 1993;24 Suppl 2:19-22.

56. McGarvey ST, Aligui G, Daniel BL, Peters P, Olveda R, Olds GR. Child growth and Schistosomiasis japonica in Northeastern Leyte, the Philippines: Cross-sectional results. Am J Trop Med Hyg. 1992;46(5):571-81.

57. Auer C. Health status of children living in a squatter area of Manila, Philippines, with particular emphasis on intestinal parasitoses. The Southeast Asian journal of tropical medicine and public health. 1990;21(2):289-300.

58. Laws HF, 2nd, Enriquez M. The prevalence of parasitism in preschool Americans in the Philippines. Mil Med. 1990;155(12):585-7.

59. Oberst RB, Alquiza LM. Survey of intestinal parasites on Palawan, Philippines. The Southeast Asian journal of tropical medicine and public health. 1987;18(2):197-201.

60. Nakao M, Matsuda H, Tanaka H, Santos AT, Jr., Blas BL, Nakamura S. Negative conversion of COP (circumoval precipitation) reactions after selective mass chemotherapy on schistosomiasis japonica with praziquantel in Bohol, Philippines. Japanese Journal of Experimental Medicine. 1987;57(5):261-6.

61. Fuentes CC. Double blind randomized study on the antihelminthic effect ofipil-ipil seed powder against Ascaris lumbricoides. Cebu Doctors' Proceedings. 1987;5(1):18-26.

62. Tanaka H, Blas BL, Nosenas JS, Matsuda H, Hayashi Y, Santos AT, Jr. Longitudinal study on Schistosoma japonicum infections in the Philippines. 4. Effect of mass-chemotherapy with praziquantel on incidence at Dagami, Leyte. Japanese Journal of Experimental Medicine. 1985;55(4):161-5.

63. Yogore MG, Lewert RM, Blas BL. SEROEPIDEMIOLOGY OF SCHISTOSOMIASIS-JAPONICA BY ELISA IN THE PHILIPPINES .3. SELECTIVE MASS CHEMOTHERAPY WITH PRAZIQUANTEL IN A CONTROL PROGRAM. Am J Trop Med Hyg. 1984;33(5):882-90.

64. Cabrera BD. Reinfection and infection rates of ascariasis in relation to seasonal variation in the Philippines. The Southeast Asian journal of tropical medicine and public health. 1984;15(3):394-401.

65. Tanaka H, Blas BL, Nosenas JS, Matsuda H, Hayashi Y, Santos AT, Jr. Epidemiology and transmission dynamics; evaluation of control measures by means of annual incidence of infection with Schistosoma japonicum among school children at Dagami, Leyte, Philippines. Southeast Asian Journal of Tropical Medicine and Public Health. 1984;15(4):480-1.

66. Tiu E, Fevidal Jr P, de Veyra Jr F, Icatlo Jr FC, Domingo EO. Relationship of prevalence and intensity of infection to morbidity in schistosomiasis japonica: A study of three communities in Leyte, Philippines. Am J Trop Med Hyg. 1983;32(6):1312-21.

67. Yogore MG, Lewert RM, Blas BL. SEROEPIDEMIOLOGY OF SCHISTOSOMIASIS JAPONICA BY ELISA IN THE PHILIPPINES .1. UNDERESTIMATION BY STOOL EXAMINATION OF THE PREVALENCE OF INFECTION IN SCHOOL-CHILDREN. Am J Trop Med Hyg. 1983;32(6):1322-34.

68. Tanaka H, Blas BL, Nosenas JS, Matsuda H, Ishige M, Kamiya H, et al. Longitudinal study on Schistosoma japonicum infections in the Philippines. 3. Incidence and environmental modification at Dagami, Leyte. Japanese Journal of Experimental Medicine. 1983;53(2):87-94.

69. Cabrera BD, Cruz AC. A comparative study on the effect of mass treatment of the entire community and selective treatment of children on the total prevalence of soil-transmitted helminthiasis in two communities, Mindoro, Philippines. Transactions of the National Academy of Science and Technology. 1983;5(6):97-124.

70. Olveda RM, Icatlo FC, Jr., Libranda BD, Fevidal P, Jr., Domingo EO. A community-based clinical trial of albendazole in Leyte, Philippines. Philippine Journal of Internal Medicine. 1983;21(3):126-33.

71. Ishii A, Cabrera BD, Suguri S, Kobayashi M, Go TG, Valeza F. An epidemiological study of filariasis in Sorsogon province, Republic of the Philippines, with notes on experimental mosquito infection. Journal of Tropical Medicine and Hygiene. 1983;86(2):59-64.

72. Tomita M, Nakagawa T, Caragay R. The survey of parasitosis (I) in Bay (Philippines). Kobe Journal of Medical Sciences. 1982;28(1):1-5.

73. Enarson DA, Enarson PM. Filariasis in an upland population in the Philippines. Tropical and Geographical Medicine. 1982;34(4):353-8.

74. Shibuya T, Tanaka H, Cabrera BD, Valesa FS, Instrella R, Go T, et al. Low density microfilaremia of Wuchereria bancrofti infections in pre- and post-treatment phases in the Philippines. Japanese Journal of Experimental Medicine. 1981;51(2):133-5.

75. Carney WP, De Veyra V, Cala EM, Cross JH. Intestinal parasites of man in Bukidnon, Philippines, with emphasis on schistosomiasis. Southeast Asian Journal of Tropical Medicine and Public Health. 1981;12(1):24-9.

76. Carney WP, Banzon T, de Veyra V, Papasin MC, Cross JH. Intestinal parasites of man in Oriental Mindoro, Philippines, with emphasis on schistosomiasis. Southeast Asian J Trop Med Public Health. 1981;12(1):12-8.

77. Tiu E, Peters PA, Warren KS, Mahmoud AA, Houser HB. Morbidity in Schistosomiasis japonica in relation to intensity of infection: Study of a community in Leyte, Philippines. Am J Trop Med Hyg. 1980;29(5):I.

78. World Health Organisation. QUANTITATIVE ASPECTS OF THE EPIDEMIOLOGY OF SCHISTOSOMA-JAPONICUM INFECTION IN A RURAL-COMMUNITY OF LUZON, PHILIPPINES. Bull World Health Organ. 1980;58(4):629-38.

79. Tanaka H, Nakai H, Omoto K, Shibuya T, Hirai M, Mercado AS, et al. The high prevalence of Wuchereria bancrofti infections in indigenous tribes in Northern Mindanao, Philippines. Japanese Journal of Experimental Medicine. 1980;50(2):85-9.

80. Carney WP, Banzon T, de Veyra V, Dana E, Cross JH. Intestinal parasites of man in northern Bohol, Philippines, with emphasis on schistosomiasis. Southeast Asian Journal of Tropical Medicine and Public Health. 1980;11(4):473-9.

81. Blas BL, Nosenas JS, Tanaka H, Matsudo O, Onodera N, Matsuda H, et al. Longitudinal study on Schistosoma japonicum infections in the Philippines. Incidence and prevalence among school children in Dagami Area, Leyte. Japanese Journal of Experimental Medicine. 1979;49(2):107-15.

82. Cabrera BD, Sy FS. Oxantel-pyrantel in various regimens for the treatment of soil transmitted helminthiasis in rural and urban communities. Drugs. 1978;15(Suppl. 1):78-85.

83. Cabrera BD, Valeza F, Santos AT, Jr., Cruz I. Current status of schistosomiasis japonica in Sorsogon Province, Republic of the Philippines. Southeast Asian J Trop Med Public Health. 1978;9(1):86-92.

84. Grove DI, Valeza FS, Cabrera BD. Bancroftian filariasis in a Philippine village: clinical, parasitological, immunological, and social aspects. Bull World Health Organ. 1978;56(6):975-84.

85. F Valeza, Cabrera B. The reinfection rate of soil-transmitted helminth in the pilot areas after treatment. Asian Parasite Control Organization [APCO]: Collec.

86. Cross JH, Banzon T, Wheeling CH, Cometa H, Lien JC, Clarke R, et al. Biomedical survey in North Samar Province, Philippine Islands. Southeast Asian J Trop Med Public Health. 1977;8(4):464-75.

87. Banzon TC, Singson CN, Cross JH. Mebendazole treatment for intestinal nematodes in a Philippine barrio. [not specified]. Journal of the Philippine Islands Medical Association. 1976;52(7/8):239-43.

88. Cabrera BD, Arambulo PV, III, Portillo GP. Ascariasis control and/or eradication in a rural community in the Philippines. Southeast Asian Journal of Tropical Medicine and Public Health. 1975;6(4):510-8.

89. Villanueva N MA, De Castro CR, Rapanot N, Tantengco VO. Nutritional anemia in Filipino school children. Journal of the Philippine Medical Association. 1973.

90. Jueco NL, Palaypay MA, Aceremo L. Prevalence and intensity of intestinal parasitism on Talim Island, Binangonan, Rizal, Philippines. Southeast Asian J Trop Med Public Health. 1973;4(4):582-7.

91. Cabrera BD, Valeza F. Filariasis in Sablayan Island, Juban, Sorsogon. Acta Med Philippina. 1972;8(4):145-51.

92. Wenceslao JM, Oban E, Cabrera BD. Eastern Samar, the fourth endemic focus for Malayan filariasis in the Republic of the Philippines. Southeast Asian Journal of Tropical Medicine and Public Health. 1972;3(4):552-61.

93. Cabrera BD, Jueco NL. Filariasis survey among indigenous tribes of Palawan, Republic of the Philippines. Stheast Asian J. 1972;Trop. Med. Publ. Hlth. 3(1):31-9.

94. Jueco NL, Cabrera BD. Reinfection rates of successfully treated cases of ascariasis in Victoria, Laguna. [not specified]. Journal of the Philippine Islands Medical Association. 1971;47(9):449-54.

95. Celis GR, Viloria EV. A parasitological survey of Leveriza, Malate, Manila. [not specified]. Acta Med Philippina. 1970;6(3):95-101.

96. Cabrera BD, Cruz I. The second endemic locus for malayan filariasis in the repubUc of the Philippines. Acta Medphilipp. 1968;5(1):1-24.

97. Cabrera BD, Tamondong CT. Bancroftian and malayan filariasis in palawan. Extent and distribution. Acta Med Philippina. 1966;3(1):20-36.

98. Rozeboom LE, Cabrera BD. FILARIASIS CAUSED BY WUCHERERIA-BANCROFTI IN PALAWAN, REPUBLIC-OF-THE-PHILIPPINES. Am J Epidemiol. 1965;81(2):216-21.

99. Estrada JP, Basio DG. Filariasis in the Philippines. [not specified]. Journal of the Philippine Islands Medical Association. 1965;41(2):100-53.

100. Rozeboom LE, Cabrera BD. FILARIASIS CAUSED BY BRUGIA-MALAYI IN THE REPUBLIC-OF-THE-PHILIPPINES. Am J Epidemiol. 1965;81(2):200-15.

101. Rozeboom LE, Cabrera BD. Filariasis in Mountain Province, Luzon, Republic of the Philippines. [not specified]. Journal of Medical Entomology. 1964;1(1):18-28.

102. Kuntz RE. Intestinal parasites of man in Leyte. Acta Med Philippina. 1964;1(1):5-10.

103. Kuntz R. Intestinal Parasites of Man in Palawan, Republic of the Philippines. Journal of the Philippine Islands Medical Association. 1963;39(7):590-600.

104. Pesigan TP, Hairston NG. The effect of snail control on the prevalence of Schistosoma japonicum infection in the Philippines. [not specified]. Bull World Health Organ. 1961;25(4/5):479-82.

105. Baisas FE. Notes on Philippine mosquitoes, XIX. The mosquito problem in the control of fllariasis in Sorsogon Province. [not specified]. Philippine Journal of Science. 1958;86(1):71-120.

106. Pesigan TP, Farooq M, Hairston NG, Jauregui JJ, Garcia EG, Santos AT, et al. Studies on Schistosoma japonicum infection in the Philippines. 1. General considerations and epidemiology. Bull World Health Organ. 1958;18(3):345-455.

107. Calubaquib PB, Rolda H. The Incidence of Intestinal Parasitism among Food Handlers. [not specified]. Journal of the Philippine Islands Medical Association. 1947;23(4):149-52.
