# Supplementary material for: Prevalence of soil-transmitted helminth infections, schistosomiasis, and lymphatic filariasis before and after preventive chemotherapy initiation in the Philippines: A systematic review and meta-analysis
Source: PLoS Negl Trop Dis. 2021 Dec 20;15(12):e0010026. doi: 10.1371/journal.pntd.0010026 (PMC8722724; doi:10.1371/journal.pntd.0010026)
Supplement: S2 Table — (DOCX) [file pntd.0010026.s004.docx]

**S2 Table. Studies which reported STH and schistosomiasis prevalence**

| **Reference** | **Site (Province/Highly-urbanised cities)** | **Clusters** | **Pop** | **Year** | **Test** | **N** | ***Ascaris*** | | **MHI *A. lumbricoides*** | | | ***Trichuris trichiura*** | | **MHI *T. trichiura*** | | | **Hookworm** | | **MHI Hookworm** | | | **Schisto somiasis** | | **MHI Schisto somiasis** | |
| --- | --- | --- | --- | --- | --- | --- | --- | --- | --- | --- | --- | --- | --- | --- | --- | --- | --- | --- | --- | --- | --- | --- | --- | --- | --- |
|  |  |  |  |  |  |  | **n** | **%** | **n** | | **%** | **n** | **%** | **n** | | **%** | **n** | **%** | **n** | | **%** | **n** | **%** | **n** | **%** |
| **POST-PREVENTIVE CHEMOTHERAPY INITIATION** | | | | | | | | | | | | | | | | | | | | | | | | | |
| [1] | La Union | community-based, number of clusters not described | GP | 2018 | KK | 323 | 50 | 15.5 | 25 | 7.7 | | 70 | 21.7 | 21 | 6.5 | | 6 | 1.9 | 1 | 0.3 | | 0 | 0.0 | 0 | 0.0 |
|  | Batanes |  |  |  |  | 370 | 0 | 0.0 | 0 | 0.0 | | 0 | 0.0 | 0 | 0.0 | | 0 | 0.0 | 0 | 0.0 | | 0 | 0.0 | 0 | 0.0 |
|  | Batanes |  |  |  |  | 351 | 1 | 0.3 | 1 | 0.3 | | 2 | 0.6 | 0 | 0.0 | | 0 | 0.0 | 0 | 0.0 | | 0 | 0.0 | 0 | 0.0 |
|  | Quirino |  |  |  |  | 422 | 11 | 2.6 | 5 | 1.2 | | 2 | 0.5 | 0 | 0.0 | | 0 | 0.0 | 0 | 0.0 | | 0 | 0.0 | 0 | 0.0 |
|  | Oriental mindoro |  |  |  |  | 947 | 133 | 14.0 | 58 | 6.1 | | 151 | 15.9 | 26 | 2.7 | | 61 | 6.4 | 3 | 0.3 | | 2 | 0.2 | 1 | 0.1 |
|  | Oriental mindoro |  |  |  |  | 603 | 49 | 8.1 | 12 | 2.0 | | 37 | 6.1 | 1 | 0.2 | | 16 | 2.7 | 0 | 0.0 | | 7 | 1.2 | 2 | 0.3 |
|  | Sorsogon |  |  |  |  | 899 | 266 | 29.6 | 95 | 10.6 | | 448 | 49.8 | 59 | 6.6 | | 3 | 0.3 | 0 | 0.0 | | 9 | 1.0 | 2 | 0.2 |
|  | Capiz |  |  |  |  | 394 | 94 | 23.9 | 64 | 16.2 | | 154 | 39.1 | 53 | 13.5 | | 5 | 1.3 | 0 | 0.0 | | 0 | 0.0 | 0 | 0.0 |
|  | Capiz |  |  |  |  | 568 | 101 | 17.8 | 36 | 6.3 | | 177 | 31.2 | 44 | 7.7 | | 14 | 2.5 | 0 | 0.0 | | 0 | 0.0 | 0 | 0.0 |
|  | Negros occidental |  |  |  |  | 1082 | 47 | 4.3 | 6 | 0.6 | | 115 | 10.6 | 4 | 0.4 | | 165 | 15.2 | 1 | 0.1 | | 17 | 1.6 | 1 | 0.1 |
|  | Siquijor |  |  |  |  | 748 | 9 | 1.2 | 0 | 0.0 | | 14 | 1.9 | 1 | 0.1 | | 8 | 1.1 | 0 | 0.0 | | 0 | 0.0 | 0 | 0.0 |
|  | Eastern samar |  |  |  |  | 1120 | 172 | 15.4 | 61 | 5.4 | | 127 | 11.3 | 4 | 0.4 | | 7 | 0.6 | 0 | 0.0 | | 17 | 1.5 | 3 | 0.3 |
|  | Zamboanga del Norte |  |  |  |  | 997 | 41 | 4.1 | 10 | 1.0 | | 51 | 5.1 | 3 | 0.3 | | 21 | 2.1 | 0 | 0.0 | | 4 | 0.4 | 0 | 0.0 |
|  | Camiguin |  |  |  |  | 532 | 50 | 9.4 | 21 | 3.9 | | 82 | 15.4 | 4 | 0.8 | | 10 | 1.9 | 3 | 0.6 | | 0 | 0.0 | 0 | 0.0 |
|  | Compostela valley |  |  |  |  | 2103 | 32 | 1.5 | 5 | 0.2 | | 23 | 1.1 | 0 | 0.0 | | 11 | 0.5 | 0 | 0.0 | | 9 | 0.4 | 1 | 0.0 |
|  | Sarangani |  |  |  |  | 866 | 62 | 7.2 | 24 | 2.8 | | 71 | 8.2 | 5 | 0.6 | | 13 | 1.5 | 0 | 0.0 | | 0 | 0.0 | 0 | 0.0 |
| [2] | Northern Samar | 22 brgys, 2 mun | GP | 2012 | KK | 10434 | 4200 | 40.3 |  | | | 6512 | 62.4 |  | | | 3268 | 31.3 |  | | | 2831 | 27.1 |  | |
| [3] | Northern Samar | 18 brgys, 2 mun | GP | 2012 | KK | 6976 | 2546 | 36.5 |  |  |  | 4311 | 61.8 |  |  |  | 1981 | 28.4 |  |  |  | 2016 | 28.9 |  |  |
| [4] | Leyte | 4 brgys, 1 mun | Children | 2015 | KK | 533 | 182 | 34.1 | 109 | | 20.5 | 286 | 53.7 | 89 | | 16.7 | 14 | 2.6 | 0 | | 0.0 | 60 | 11.3 | 18 | 3.4 |
|  |  |  | Adults |  |  | 418 | 110 | 26.3 | 46 | | 11.0 | 206 | 49.3 | 41 | | 9.8 | 43 | 10.3 | 2 | | 0.5 | 56 | 13.4 | 8 | 1.9 |
| [5] | Ilocos Norte | 5 schools | SAC | 2015 | KK | 203 | 0 | 0.0 | 0 | | 0.0 | 1 | 0.5 | 0 | | 0.0 | 0 | 0.0 | 0 | | 0.0 | 1 | 0.5 | 0 | 0.0 |
|  | Ilocos Sur |  |  | 2015 |  | 279 | 43 | 15.4 | 23 | | 8.2 | 55 | 19.7 | 14 | | 5.0 | 0 | 0.0 | 0 | | 0.0 | 0 | 0.0 | 0 | 0.0 |
|  | La Union |  |  |  |  | 231 | 25 | 10.8 | 7 | | 3.0 | 65 | 28.1 | 12 | | 5.2 | 0 | 0.0 | 0 | | 0.0 | 0 | 0.0 | 0 | 0.0 |
|  | Pangasinan |  |  |  |  | 179 | 24 | 13.4 | 9 | | 5.0 | 20 | 11.2 | 0 | | 0.0 | 0 | 0.0 | 0 | | 0.0 | 0 | 0.0 | 0 | 0.0 |
|  | Batanes |  |  |  |  | 155 | 3 | 1.9 | 0 | | 0.0 | 1 | 0.6 | 0 | | 0.0 | 0 | 0.0 | 0 | | 0.0 | 0 | 0.0 | 0 | 0.0 |
|  | Cagayan | 10 schools |  |  |  | 493 | 36 | 7.3 | 12 | | 2.4 | 107 | 21.7 | 27 | | 5.5 | 0 | 0.0 | 0 | | 0.0 | 12 | 2.4 | 2 | 0.4 |
|  | Isabela | 5 schools |  |  |  | 282 | 18 | 6.4 | 3 | | 1.1 | 8 | 2.8 | 0 | | 0.0 | 0 | 0.0 | 0 | | 0.0 | 0 | 0.0 | 0 | 0.0 |
|  | Nueva Vizcaya |  |  |  |  | 131 | 14 | 10.7 | 4 | | 3.1 | 6 | 4.6 | 1 | | 0.8 | 0 | 0.0 | 0 | | 0.0 | 0 | 0.0 | 0 | 0.0 |
|  | Quirino |  |  |  |  | 307 | 11 | 3.6 | 2 | | 0.7 | 7 | 2.3 | 0 | | 0.0 | 0 | 0.0 | 0 | | 0.0 | 0 | 0.0 | 0 | 0.0 |
|  | Aurora |  |  |  |  | 174 | 41 | 23.6 | 15 | | 8.6 | 37 | 21.3 | 1 | | 0.6 | 0 | 0.0 | 0 | | 0.0 | 0 | 0.0 | 0 | 0.0 |
|  | Bataan |  |  |  |  | 142 | 16 | 11.3 | 12 | | 8.5 | 29 | 20.4 | 4 | | 2.8 | 0 | 0.0 | 0 | | 0.0 | 0 | 0.0 | 0 | 0.0 |
|  | Bulacan |  |  |  |  | 215 | 21 | 9.8 | 1 | | 0.5 | 11 | 5.1 | 3 | | 1.4 | 0 | 0.0 | 0 | | 0.0 | 0 | 0.0 | 0 | 0.0 |
|  | Nueva Ecija |  |  |  |  | 228 | 38 | 16.7 | 14 | | 6.1 | 24 | 10.5 | 5 | | 2.2 | 0 | 0.0 | 0 | | 0.0 | 0 | 0.0 | 0 | 0.0 |
|  | Pampanga |  |  |  |  | 234 | 68 | 29.1 | 31 | | 13.2 | 88 | 37.6 | 9 | | 3.8 | 0 | 0.0 | 0 | | 0.0 | 0 | 0.0 | 0 | 0.0 |
|  | Tarlac |  |  |  |  | 195 | 55 | 28.2 | 19 | | 9.7 | 55 | 28.2 | 5 | | 2.6 | 0 | 0.0 | 0 | | 0.0 | 0 | 0.0 | 0 | 0.0 |
|  | Zambales |  |  |  |  | 140 | 32 | 22.9 | 3 | | 2.1 | 41 | 29.3 | 1 | | 0.7 | 0 | 0.0 | 0 | | 0.0 | 0 | 0.0 | 0 | 0.0 |
|  | Batangas |  |  |  |  | 244 | 26 | 10.7 | 12 | | 4.9 | 17 | 7.0 | 4 | | 1.6 | 0 | 0.0 | 0 | | 0.0 | 0 | 0.0 | 0 | 0.0 |
|  | Cavite |  |  |  |  | 260 | 48 | 18.5 | 25 | | 9.6 | 26 | 10.0 | 4 | | 1.5 | 0 | 0.0 | 0 | | 0.0 | 0 | 0.0 | 0 | 0.0 |
|  | Laguna |  |  |  |  | 188 | 26 | 13.8 | 11 | | 5.9 | 13 | 6.9 | 2 | | 1.1 | 0 | 0.0 | 0 | | 0.0 | 0 | 0.0 | 0 | 0.0 |
|  | Quezon |  |  |  |  | 204 | 21 | 10.3 | 5 | | 2.5 | 18 | 8.8 | 0 | | 0.0 | 0 | 0.0 | 0 | | 0.0 | 0 | 0.0 | 0 | 0.0 |
|  | Rizal |  |  |  |  | 255 | 64 | 25.1 | 17 | | 6.7 | 13 | 5.1 | 3 | | 1.2 | 0 | 0.0 | 0 | | 0.0 | 0 | 0.0 | 0 | 0.0 |
|  | Marinduque |  |  |  |  | 215 | 50 | 23.3 | 20 | | 9.3 | 61 | 28.4 | 12 | | 5.6 | 1 | 0.5 | 0 | | 0.0 | 0 | 0.0 | 0 | 0.0 |
|  | Occidental Mindoro |  |  |  |  | 222 | 76 | 34.2 | 34 | | 15.3 | 84 | 37.8 | 14 | | 6.3 | 0 | 0.0 | 0 | | 0.0 | 0 | 0.0 | 0 | 0.0 |
|  | Oriental Mindoro | 10 schools |  | 2015 |  | 481 | 92 | 19.1 | 36 | | 7.5 | 72 | 15.0 | 7 | | 1.5 | 1 | 0.2 | 0 | | 0.0 | 1 | 0.2 | 0 | 0.0 |
|  | Palawan | 5 schools |  |  |  | 275 | 87 | 31.6 | 57 | | 20.7 | 113 | 41.1 | 28 | | 10.2 | 2 | 0.7 | 0 | | 0.0 | 0 | 0.0 | 0 | 0.0 |
|  | Romblon |  |  |  |  | 259 | 70 | 27.0 | 41 | | 15.8 | 87 | 33.6 | 4 | | 1.5 | 1 | 0.4 | 0 | | 0.0 | 0 | 0.0 | 0 | 0.0 |
|  | Albay | 5 schools |  | 2015 |  | 241 | 87 | 36.1 | 33 | | 13.7 | 62 | 25.7 | 5 | | 2.1 | 0 | 0.0 | 0 | | 0.0 | 0 | 0.0 | 0 | 0.0 |
|  | Camarines Norte |  |  |  |  | 154 | 72 | 46.8 | 44 | | 28.6 | 48 | 31.2 | 9 | | 5.8 | 0 | 0.0 | 0 | | 0.0 | 0 | 0.0 | 0 | 0.0 |
|  | Camarines Sur |  |  |  |  | 255 | 102 | 40.0 | 52 | | 20.4 | 86 | 33.7 | 15 | | 5.9 | 0 | 0.0 | 0 | | 0.0 | 0 | 0.0 | 0 | 0.0 |
|  | Catanduanes |  |  |  |  | 202 | 78 | 38.6 | 41 | | 20.3 | 101 | 50.0 | 45 | | 22.3 | 1 | 0.5 | 0 | | 0.0 | 0 | 0.0 | 0 | 0.0 |
|  | Masbate |  |  |  |  | 236 | 133 | 56.4 | 77 | | 32.6 | 157 | 66.5 | 40 | | 16.9 | 1 | 0.4 | 0 | | 0.0 | 0 | 0.0 | 0 | 0.0 |
|  | Sorsogon | 10 schools |  |  |  | 429 | 292 | 68.1 | 173 | | 40.3 | 351 | 81.8 | 113 | | 26.3 | 2 | 0.5 | 0 | | 0.0 | 17 | 4.0 | 3 | 0.7 |
|  | Aklan | 5 schools |  |  |  | 316 | 57 | 18.0 | 31 | | 9.8 | 79 | 25.0 | 22 | | 7.0 | 0 | 0.0 | 0 | | 0.0 | 0 | 0.0 | 0 | 0.0 |
|  | Antique |  |  |  |  | 315 | 25 | 7.9 | 9 | | 2.9 | 58 | 18.4 | 7 | | 2.2 | 0 | 0.0 | 0 | | 0.0 | 0 | 0.0 | 0 | 0.0 |
|  | Capiz |  |  |  |  | 260 | 43 | 16.5 | 18 | | 6.9 | 97 | 37.3 | 21 | | 8.1 | 1 | 0.4 | 0 | | 0.0 | 0 | 0.0 | 0 | 0.0 |
|  | Guimaras |  |  |  |  | 283 | 16 | 5.7 | 4 | | 1.4 | 22 | 7.8 | 0 | | 0.0 | 0 | 0.0 | 0 | | 0.0 | 0 | 0.0 | 0 | 0.0 |
|  | Iloilo |  |  |  |  | 255 | 80 | 31.4 | 65 | | 25.5 | 120 | 47.1 | 52 | | 20.4 | 0 | 0.0 | 0 | | 0.0 | 0 | 0.0 | 0 | 0.0 |
|  | Negros Occidental | 10 schools |  |  |  | 619 | 68 | 11.0 | 17 | | 2.7 | 87 | 14.1 | 5 | | 0.8 | 0 | 0.0 | 0 | | 0.0 | 2 | 0.3 | 1 | 0.2 |
|  | Bohol |  |  | 2014 |  | 657 | 16 | 2.4 | 2 | | 0.3 | 147 | 22.4 | 3 | | 0.5 | 7 | 1.1 | 0 | | 0.0 | 0 | 0.0 | 0 | 0.0 |
|  | Cebu | 5 schools |  |  |  | 366 | 102 | 27.9 | 38 | | 10.4 | 60 | 16.4 | 5 | | 1.4 | 6 | 1.6 | 0 | | 0.0 | 0 | 0.0 | 0 | 0.0 |
|  | Negros Oriental |  |  |  |  | 327 | 18 | 5.5 | 1 | | 0.3 | 22 | 6.7 | 2 | | 0.6 | 4 | 1.2 | 0 | | 0.0 | 0 | 0.0 | 0 | 0.0 |
|  | Siquijor |  |  |  |  | 303 | 3 | 1.0 | 0 | | 0.0 | 8 | 2.6 | 2 | | 0.7 | 0 | 0.0 | 0 | | 0.0 | 0 | 0.0 | 0 | 0.0 |
|  | Biliran |  |  | 2015 |  | 295 | 68 | 23.1 | 32 | | 10.8 | 119 | 40.3 | 34 | | 11.5 | 0 | 0.0 | 0 | | 0.0 | 1 | 0.3 | 0 | 0.0 |
|  | Eastern Samar | 10 schools |  |  |  | 506 | 132 | 26.1 | 48 | | 9.5 | 238 | 47.0 | 43 | | 8.5 | 4 | 0.8 | 0 | | 0.0 | 1 | 0.2 | 0 | 0.0 |
|  | Leyte |  |  |  |  | 443 | 75 | 16.9 | 24 | | 5.4 | 126 | 28.4 | 18 | | 4.1 | 2 | 0.5 | 0 | | 0.0 | 2 | 0.5 | 1 | 0.2 |
|  | Northern Samar |  |  |  |  | 589 | 280 | 47.5 | 163 | | 27.7 | 361 | 61.3 | 143 | | 24.3 | 0 | 0.0 | 0 | | 0.0 | 59 | 10.0 | 12 | 2.0 |
|  | Southern Leyte |  |  |  |  | 264 | 60 | 22.7 | 36 | | 13.6 | 80 | 30.3 | 13 | | 4.9 | 0 | 0.0 | 0 | | 0.0 | 0 | 0.0 | 0 | 0.0 |
|  | Samar (Western Samar) |  |  |  |  | 445 | 210 | 47.2 | 114 | | 25.6 | 221 | 49.7 | 30 | | 6.7 | 2 | 0.4 | 0 | | 0.0 | 6 | 1.3 | 2 | 0.4 |
|  | Zamboanga Del Norte |  |  | 2014 |  | 546 | 135 | 24.7 | 63 | | 11.5 | 147 | 26.9 | 49 | | 9.0 | 2 | 0.4 | 0 | | 0.0 | 1 | 0.2 | 1 | 0.2 |
|  | Zamboanga Del Sur |  |  |  |  | 597 | 61 | 10.2 | 16 | | 2.7 | 50 | 8.4 | 1 | | 0.2 | 24 | 4.0 | 1 | | 0.2 | 2 | 0.3 | 1 | 0.2 |
|  | Zamboanga Sibugay |  |  |  |  | 481 | 71 | 14.8 | 21 | | 4.4 | 100 | 20.8 | 10 | | 2.1 | 10 | 2.1 | 0 | | 0.0 | 6 | 1.2 | 1 | 0.2 |
|  | Bukidnon |  |  | 2013 |  | 522 | 37 | 7.1 | 8 | | 1.5 | 12 | 2.3 | 0 | | 0.0 | 9 | 1.7 | 1 | | 0.2 | 20 | 3.8 | 2 | 0.4 |
|  | Camiguin | 5 schools |  |  |  | 231 | 22 | 9.5 | 5 | | 2.2 | 26 | 11.3 | 2 | | 0.9 | 0 | 0.0 | 0 | | 0.0 | 0 | 0.0 | 0 | 0.0 |
|  | Lanao Del Norte | 10 schools |  |  |  | 426 | 62 | 14.6 | 24 | | 5.6 | 71 | 16.7 | 3 | | 0.7 | 9 | 2.1 | 0 | | 0.0 | 5 | 1.2 | 0 | 0.0 |
|  | Misamis Occidental | 5 schools |  |  |  | 365 | 54 | 14.8 | 16 | | 4.4 | 65 | 17.8 | 10 | | 2.7 | 0 | 0.0 | 0 | | 0.0 | 2 | 0.5 | 0 | 0.0 |
|  | Misamis Oriental |  |  |  |  | 265 | 39 | 14.7 | 15 | | 5.7 | 48 | 18.1 | 6 | | 2.3 | 0 | 0.0 | 0 | | 0.0 | 0 | 0.0 | 0 | 0.0 |
|  | Compostela Valley | 10 schools |  | 2014 |  | 550 | 20 | 3.6 | 2 | | 0.4 | 21 | 3.8 | 0 | | 0.0 | 1 | 0.2 | 0 | | 0.0 | 0 | 0.0 | 0 | 0.0 |
|  | Davao Del Norte |  |  |  |  | 527 | 23 | 4.4 | 4 | | 0.8 | 23 | 4.4 | 1 | | 0.2 | 2 | 0.4 | 0 | | 0.0 | 3 | 0.6 | 2 | 0.4 |
|  | Davao Del Sur |  |  |  |  | 461 | 22 | 4.8 | 6 | | 1.3 | 25 | 5.4 | 1 | | 0.2 | 0 | 0.0 | 0 | | 0.0 | 14 | 3.0 | 2 | 0.4 |
|  | Davao Oriental |  |  |  |  | 541 | 78 | 14.4 | 2 | | 0.4 | 62 | 11.5 | 1 | | 0.2 | 0 | 0.0 | 0 | | 0.0 | 0 | 0.0 | 0 | 0.0 |
|  | Cotabato (North Cotabato) |  |  |  |  | 551 | 68 | 12.3 | 20 | | 3.6 | 69 | 12.5 | 6 | | 1.1 | 16 | 2.9 | 0 | | 0.0 | 19 | 3.4 | 18 | 3.3 |
|  | Sarangani | 5 schools |  |  |  | 265 | 54 | 20.4 | 13 | | 4.9 | 50 | 18.9 | 3 | | 1.1 | 8 | 3.0 | 0 | | 0.0 | 0 | 0.0 | 0 | 0.0 |
|  | South Cotabato | 10 schools |  |  |  | 558 | 68 | 12.2 | 15 | | 2.7 | 41 | 7.3 | 5 | | 0.9 | 5 | 0.9 | 0 | | 0.0 | 2 | 0.4 | 0 | 0.0 |
|  | Sultan Kudarat |  |  |  |  | 508 | 65 | 12.8 | 21 | | 4.1 | 74 | 14.6 | 4 | | 0.8 | 4 | 0.8 | 0 | | 0.0 | 5 | 1.0 | 1 | 0.2 |
|  | NCR 1st District | 5 schools |  | 2015 |  | 250 | 61 | 24.4 | 31 | | 12.4 | 20 | 8.0 | 1 | | 0.4 | 0 | 0.0 | 0 | | 0.0 | 0 | 0.0 | 0 | 0.0 |
|  | NCR 2nd District |  |  |  |  | 167 | 24 | 14.4 | 15 | | 9.0 | 12 | 7.2 | 1 | | 0.6 | 0 | 0.0 | 0 | | 0.0 | 0 | 0.0 | 0 | 0.0 |
|  | NCR 3rd District |  |  |  |  | 167 | 22 | 13.2 | 6 | | 3.6 | 9 | 5.4 | 1 | | 0.6 | 0 | 0.0 | 0 | | 0.0 | 0 | 0.0 | 0 | 0.0 |
|  | NCR 4th District |  |  |  |  | 141 | 15 | 10.6 | 5 | | 3.5 | 7 | 5.0 | 0 | | 0.0 | 0 | 0.0 | 0 | | 0.0 | 0 | 0.0 | 0 | 0.0 |
|  | Abra |  |  |  |  | 252 | 7 | 2.8 | 2 | | 0.8 | 6 | 2.4 | 0 | | 0.0 | 0 | 0.0 | 0 | | 0.0 | 0 | 0.0 | 0 | 0.0 |
|  | Apayao |  |  |  |  | 307 | 11 | 3.6 | 2 | | 0.7 | 13 | 4.2 | 1 | | 0.3 | 0 | 0.0 | 0 | | 0.0 | 0 | 0.0 | 0 | 0.0 |
|  | Benguet |  |  |  |  | 230 | 5 | 2.2 | 0 | | 0.0 | 3 | 1.3 | 0 | | 0.0 | 0 | 0.0 | 0 | | 0.0 | 0 | 0.0 | 0 | 0.0 |
|  | Ifugao |  |  |  |  | 282 | 14 | 5.0 | 0 | | 0.0 | 10 | 3.5 | 1 | | 0.4 | 0 | 0.0 | 0 | | 0.0 | 0 | 0.0 | 0 | 0.0 |
|  | Kalinga |  |  |  |  | 275 | 18 | 6.5 | 4 | | 1.5 | 27 | 9.8 | 1 | | 0.4 | 0 | 0.0 | 0 | | 0.0 | 0 | 0.0 | 0 | 0.0 |
|  | Mountain Province |  |  |  |  | 292 | 3 | 1.0 | 0 | | 0.0 | 15 | 5.1 | 2 | | 0.7 | 0 | 0.0 | 0 | | 0.0 | 0 | 0.0 | 0 | 0.0 |
|  | Basilan |  |  | 2014 |  | 240 | 102 | 42.5 | 48 | | 20.0 | 100 | 41.7 | 12 | | 5.0 | 1 | 0.4 | 0 | | 0.0 | 1 | 0.4 | 1 | 0.4 |
|  | Lanao Del Sur | 10 schools |  |  |  | 601 | 108 | 18.0 | 21 | | 3.5 | 75 | 12.5 | 5 | | 0.8 | 0 | 0.0 | 0 | | 0.0 | 18 | 3.0 | 3 | 0.5 |
|  | Tawi-Tawi | 5 schools |  |  |  | 197 | 57 | 28.9 | 14 | | 7.1 | 70 | 35.5 | 13 | | 6.6 | 4 | 2.0 | 0 | | 0.0 | 1 | 0.5 | 1 | 0.5 |
|  | Agusan Del Norte | 10 schools |  | 2013 |  | 369 | 57 | 15.4 | 22 | | 6.0 | 65 | 17.6 | 12 | | 3.3 | 6 | 1.6 | 1 | | 0.3 | 16 | 4.3 | 3 | 0.8 |
|  | Agusan Del Sur |  |  |  |  | 216 | 34 | 15.7 | 11 | | 5.1 | 19 | 8.8 | 0 | | 0.0 | 6 | 2.8 | 1 | | 0.5 | 7 | 3.2 | 1 | 0.5 |
|  | Dinagat Islands |  |  |  |  | 224 | 54 | 24.1 | 32 | | 14.3 | 29 | 12.9 | 2 | | 0.9 | 0 | 0.0 | 0 | | 0.0 | 1 | 0.4 | 0 | 0.0 |
|  | Surigao Del Norte |  |  |  |  | 337 | 52 | 15.4 | 37 | | 11.0 | 59 | 17.5 | 23 | | 6.8 | 2 | 0.6 | 2 | | 0.6 | 5 | 1.5 | 2 | 0.6 |
|  | Surigao Del Sur |  |  |  |  | 279 | 15 | 5.4 | 4 | | 1.4 | 27 | 9.7 | 5 | | 1.8 | 0 | 0.0 | 0 | | 0.0 | 1 | 0.4 | 0 | 0.0 |
| [6] | Northern Samar | 22 brgys, 2 mun | GP | 2012 | KK | 10434 | 4205 | 40.3 |  | | | 6511 | 62.4 |  | | | 3266 | 31.3 |  | | | 2828 | 27.1 |  | |
| [7] | Northern Samar | 6 brgys, 1 mun | GP | 2011 | qPCR | 545 | 317 | 58.2 |  |  |  |  | |  |  |  | 262 | 48.1 |  |  |  | 494 | 90.6 |  |  |
|  |  |  |  |  |  |  |  |  |  | | |  | |  | | |  |  |  |  |  |  |  |  |  |
| [8] | Bulacan | 2 mun per province | Pre-SAC | 2009 | KK | 407 | 60 | 14.7 | 30 | | 7.4 | 56 | 13.8 | 10 | | 2.5 | 0 | 0.0 |  |  |  | 0 | 0.0 |  |  |
|  | Camarines Sur |  |  |  |  | 497 | 239 | 48.1 | 161 | | 32.4 | 191 | 38.4 | 62 | | 12.5 | 0 | 0.0 |  |  |  | 0 | 0.0 |  |  |
|  | Negros Occidental |  |  |  |  | 434 | 206 | 47.5 | 149 | | 34.3 | 239 | 55.1 | 149 | | 34.3 | 8 | 1.8 |  |  |  | 0 | 0.0 |  |  |
|  | Leyte |  |  |  |  | 500 | 170 | 34.0 | 67 | | 13.4 | 222 | 44.4 | 45 | | 9.0 | 7 | 1.4 |  |  |  | 11 | 2.2 |  |  |
|  | Compostela Valley |  |  |  |  | 447 | 48 | 10.7 | 18 | | 4.0 | 20 | 4.5 | 2 | | 0.4 | 10 | 2.2 |  |  |  | 4 | 0.9 |  |  |
|  | Surigao Del Norte |  |  |  |  | 189 | 41 | 21.7 | 25 | | 13.2 | 48 | 25.4 | 22 | | 11.6 | 2 | 1.1 |  |  |  | 6 | 3.2 |  |  |
|  | Bulacan |  | SAC |  |  | 507 | 85 | 16.8 | 27 | | 5.3 | 111 | 21.9 | 8 | | 1.6 | 0 | 0.0 |  |  |  | 0 | 0.0 |  |  |
|  | Camarines Sur |  |  |  |  | 636 | 291 | 45.8 | 167 | | 26.3 | 220 | 34.6 | 64 | | 10.1 | 0 | 0.0 |  |  |  | 0 | 0.0 |  |  |
|  | Negros Occidental |  |  |  |  | 468 | 138 | 29.5 | 101 | | 21.6 | 271 | 57.9 | 139 | | 29.7 | 6 | 1.3 |  |  |  | 0 | 0.0 |  |  |
|  | Leyte |  |  |  |  | 500 | 199 | 39.8 | 99 | | 19.8 | 249 | 49.8 | 61 | | 12.2 | 19 | 3.8 |  |  |  | 6 | 1.2 |  |  |
|  | Compostela Valley |  |  |  |  | 458 | 20 | 4.4 | 5 | | 1.1 | 37 | 8.1 | 1 | | 0.2 | 25 | 5.5 |  |  |  | 12 | 2.6 |  |  |
|  | Surigao Del Norte |  |  |  |  | 182 | 29 | 15.9 | 16 | | 8.8 | 29 | 15.9 | 7 | | 3.8 | 1 | 0.5 |  |  |  | 12 | 6.6 |  |  |
| [9] | Leyte | 3 brgy | SAC |  | KK | 507 | 402 | 79.3 |  | | | 469 | 92.5 |  | | | 280 | 55.2 |  |  |  | 402 | 79.3 |  |  |
| [10] | Compostela Valley | 4 schools /brgys, 2 mun per province | SAC, adults | 2014 | KK | 525 | 12 | 2.3 |  |  |  | 6 | 1.1 |  |  |  | 20 | 3.8 |  |  |  | 16 | 3.0 |  |  |
|  | Davao Oriental |  |  |  |  | 435 | 55 | 12.6 |  |  |  | 170 | 39.1 |  |  |  | 23 | 5.3 |  |  |  | 1 | 0.2 |  |  |
|  | Agusan Del Sur |  |  |  |  | 752 | 67 | 8.9 |  |  |  | 29 | 3.9 |  |  |  | 67 | 8.9 |  |  |  | 59 | 7.8 |  |  |
|  | Surigao Del Norte |  |  |  |  | 528 | 59 | 11.2 |  |  |  | 69 | 13.1 |  |  |  | 4 | 0.8 |  |  |  | 33 | 6.3 |  |  |
| **PRE-PREVENTIVE CHEMOTHERAPY INITIATION** | | | | | | | | | | | | | | | | | | | | | | | | | |
| [11] | Surigao del Norte | 32 brgy, 4 mun | SAC | 2007 | KK | 1539 | 383 | 24.9 | 152 | | 9.9 | 517 | 33.6 | 76 | | 4.9 | 25 | 1.6 | 0 | | 0 | 167 | 43 |  | |
| [12] | Leyte | 1 brgy, 1 mun | SAC | 2002 | KK | 322 | 240 | 74.5 |  | |  | 298 | 92.5 |  | |  | 149 | 46.3 |  | | | 251 | 78.0 |  |  |
| [13] | Bulacan | 8 schools, 2 mun per province | SAC | 2006 | KK | 466 | 102 | 21.9 | 18 | | 3.9 | 112 | 24.0 | 4 | | 0.9 | 16 | 3.4 | 1 | | 0.2 | 0 | 0.0 |  |  |
|  | Camarines Sur |  |  |  |  | 602 | 279 | 46.3 | 171 | | 28.4 | 241 | 40.0 | 65 | | 10.8 | 3 | 0.5 | 0 | | 0.0 | 0 | 0.0 |  |  |
|  | Negros Occidental |  |  |  |  | 527 | 233 | 44.2 | 134 | | 25.4 | 315 | 59.8 | 157 | | 29.8 | 11 | 2.1 | 1 | | 0.2 | 3 | 0.6 |  |  |
|  | Leyte |  |  |  |  | 526 | 272 | 51.7 | 108 | | 20.5 | 247 | 47.0 | 47 | | 8.9 | 39 | 7.4 | 10 | | 1.9 | 3 | 0.6 |  |  |
|  | Compostela Valley |  |  |  |  | 600 | 126 | 21.0 | 50 | | 8.3 | 87 | 14.5 | 8 | | 1.3 | 45 | 7.5 | 2 | | 0.3 | 11 | 1.8 |  |  |
|  | Surigao Del Norte |  |  |  |  | 652 | 289 | 44.3 | 185 | | 28.4 | 298 | 45.7 | 64 | | 9.8 | 13 | 2.0 | 0 | | 0.0 | 24 | 3.7 |  |  |
| [14] | Leyte | 1 village, 1 mun | SAC |  | KK | 319 | 236 | 73.9 |  | | | 294 | 92.2 |  | | | 146 | 45.8 |  | | | 244 | 76.5 |  |  |
| [15] | Leyte | 6 brgys | GP | 1989 | KK | 1561 | 1273 | 81.5 |  |  |  | 1299 | 83.2 |  |  |  | 476 | 30.5 |  |  |  | 418 | 26.8 |  |  |
| [16] | Bukidnon | 4 brgys, 1 mun | GP | 1978 | FECT, Harada Mori | 831 | 268 | 32.3 |  |  |  | 98 | 11.8 |  |  |  | 363 | 43.7 |  |  |  | 135 | 16.2 |  |  |
| [17] | Oriental Mindoro | 4 brgys, 1 mun | GP | 1978 | DFS, FECT, Harada Mori | 1058 | 683 | 64.6 |  |  |  | 695 | 65.7 |  |  |  | 430 | 40.6 |  |  |  | 211 | 19.9 |  |  |
| [18] | Bohol | 8 brgys, 2 mun | GP | 1977 | DFS, FECT, Harada Mori | 1694 | 754 | 44.5 |  |  |  | 979 | 57.8 |  |  |  | 1210 | 71.4 |  |  |  | 91 | 5.4 |  |  |
| [19] | Northern Samar | 8 brgys | GP | 1973 | DFS | 1394 | 1100 | 78.9 |  |  |  | 1261 | 90.5 |  |  |  | 901 | 64.6 |  |  |  | 203 | 14.6 |  |  |
| [20] | Leyte | 1 brgy, 1 mun | GP | 1974 | FECT | 472 |  | 0.0 |  |  |  | 373 | 79.0 |  |  |  | 60 | 12.7 |  |  |  | 27 | 5.7 |  |  |
| [21] | Leyte | 1 mun | GP |  | MIFC | 304 | 286 | 94.0 |  |  |  | 277 | 91.0 |  |  |  | 112 | 37.0 |  |  |  | 40 | 13.0 |  |  |
| [22] | Leyte | 1 mun | GP | 1955 | MIFC, Stoll | 2909 | 2365 | 81.3 |  |  |  | 2561 | 88.0 |  |  |  | 1918 | 65.9 |  |  |  | 1395 | 48.0 |  |  |

**References**

1. Palasi L, Galit, Tangcalagan, Bernal, Eugenio, 2018. Community-Based Survey on the Prevalence of Soil-Transmitted Helminths, Schistosomiasis, and Other Intestinal Parasitic Infections among Preschool Age Children, Adolescents and Adults [DRAFT]. 2018.

2. Yu W, Ross AG, Olveda RM, Harn DA, Li Y, Chy D, et al. Risk of human helminthiases: geospatial distribution and targeted control. Int J Infect Dis. 2017;55:131-8.

3. Ross AG, Olveda RM, McManus DP, Harn DA, Chy D, Li Y, et al. Risk factors for human helminthiases in rural Philippines. Int J Infect Dis. 2017;54:150-5.

4. Liwanag HJ, Uy J, Bataller R, Gatchalian JR, De La Calzada B, Uy JA, et al. Soil-transmitted helminthiasis and schistosomiasis in children of poor families in leyte, Philippines: Lessons for disease prevention and control. J Trop Pediatr. 2017;63(5):335-45.

5. RITM. National Survey on the Prevalence of Soil-Transmitted Helminths (STH), Schistosomiasis and other Intestinal Parasitic Infections among Public School Children in the Philippines [DRAFT]. 2016.

6. Ross AG, Olveda RM, Chy D, Olveda DU, Li Y, Harn DA, et al. Can mass drug administration lead to the sustainable control of schistosomiasis? J Infect Dis. 2015;211(2):283-9.

7. Gordon CA, McManus DP, Acosta LP, Olveda RM, Williams GM, Ross AG, et al. Multiplex real-time PCR monitoring of intestinal helminths in humans reveals widespread polyparasitism in Northern Samar, the Philippines. Int J Parasitol. 2015;45(7):477-83.

8. Belizario VY, Totanes FIG, De Leon WU, Ciro RNT, Lumampao YF. Sentinel surveillance of soil-transmitted helminthiasis in preschool-aged and school-aged children in selected local government units in the Philippines: Follow-up assessment. Asia-Pac J Public Health. 2015;27(2):NP1604-NP15.

9. Ezeamama AE, McGarvey ST, Acosta LP, Zierler S, Manalo DL, Wu HW, et al. The synergistic effect of concomitant schistosomiasis, hookworm, and trichuris infections on children's anemia burden. PLoS Negl Trop Dis. 2008;2(6):e245.

10. Vicente Y. Belizario, Arlene G. Bertuso, Jolene Kristine G. Gatmaitan, Myra S. Mistica, Janis Ruth C. Gatchalian, Chua PLC. Food-Borne Helminth Infections in Selected Sites in the Philippines. Manila; 2015.

11. Cassion CC, Pingal ET, Maniago RB, Medina JRC, Belizario Jr VY. Schistosomiasis and soil-transmitted helminth infections in school children in the Lake Mainit area in Northeastern Mindanao: An opportunity for integrated helminth control in the school setting. Acta Medica Philippina. 2013;47(3):4-10.

12. Olson CL, Acosta LP, Hochberg NS, Olveda RM, Jiz M, McGarvey ST, et al. Anemia of inflammation is related to cognitive impairment among children in Leyte, The Philippines. PLoS Neglected Tropical Diseases. 2009;3 (10) (no pagination)(e533).

13. Belizario VY, Jr., Leon WUd, Lumampao YF, Anastacio MBM, Tai CMC. Sentinel surveillance of soil-transmitted helminthiasis in selected local government units in the Philippines. Asia Pacific Journal of Public Health. 2009;21(1):26-42.

14. Ezeamama AE, Friedman JF, Acosta LP, Bellinger DC, Langdon GC, Manalo DL, et al. Helminth infection and cognitive impairment among Filipino children. American Journal of Tropical Medicine and Hygiene. 2005;72(5):540-8.

15. McGarvey ST, Aligui G, Daniel BL, Peters P, Olveda R, Olds GR. Child growth and Schistosomiasis japonica in Northeastern Leyte, the Philippines: Cross-sectional results. American Journal of Tropical Medicine and Hygiene. 1992;46(5):571-81.

16. Carney WP, De Veyra V, Cala EM, Cross JH. Intestinal parasites of man in Bukidnon, Philippines, with emphasis on schistosomiasis. Southeast Asian Journal of Tropical Medicine and Public Health. 1981;12(1):24-9.

17. Carney WP, Banzon T, de Veyra V, Papasin MC, Cross JH. Intestinal parasites of man in Oriental Mindoro, Philippines, with emphasis on schistosomiasis. Southeast Asian J Trop Med Public Health. 1981;12(1):12-8.

18. Carney WP, Banzon T, de Veyra V, Dana E, Cross JH. Intestinal parasites of man in northern Bohol, Philippines, with emphasis on schistosomiasis. Southeast Asian Journal of Tropical Medicine and Public Health. 1980;11(4):473-9.

19. Cross JH, Banzon T, Wheeling CH, Cometa H, Lien JC, Clarke R, et al. Biomedical survey in North Samar Province, Philippine Islands. Southeast Asian J Trop Med Public Health. 1977;8(4):464-75.

20. Cabrera BD, Arambulo PV, III, Portillo GP. Ascariasis control and/or eradication in a rural community in the Philippines. Southeast Asian Journal of Tropical Medicine and Public Health. 1975;6(4):510-8.

21. Kuntz RE. Intestinal parasites of man in Leyte. Acta Medica Philippina. 1964;1(1):5-10.

22. Pesigan TP, Farooq M, Hairston NG, Jauregui JJ, Garcia EG, Santos AT, et al. Studies on Schistosoma japonicum infection in the Philippines. 1. General considerations and epidemiology. Bull World Health Organ. 1958;18(3):345-455.
