# Supplementary material for: Prevalence of soil-transmitted helminth infections, schistosomiasis, and lymphatic filariasis before and after preventive chemotherapy initiation in the Philippines: A systematic review and meta-analysis
Source: PLoS Negl Trop Dis. 2021 Dec 20;15(12):e0010026. doi: 10.1371/journal.pntd.0010026 (PMC8722724; doi:10.1371/journal.pntd.0010026)
Supplement: S3 Table — (DOCX) [file pntd.0010026.s005.docx]

**S3 Table. Studies which reported STH prevalence only**

| **Reference** | **Site (Province/Highly-urbanised cities)** | **Clusters** | **Pop** | **Year** | **Test** | **N** | ***Ascaris***  ***lumbricoides*** | | **MHI *A. lumbricoides*** | | ***Trichuris trichiura*** | | **MHI *T. trichiura*** | | **Hookworm** | | **MHI Hookworm** | |
| --- | --- | --- | --- | --- | --- | --- | --- | --- | --- | --- | --- | --- | --- | --- | --- | --- | --- | --- |
|  |  |  |  |  |  |  | **n** | **%** | **n** | **%** | **n** | **%** | **n** | **%** | **n** | **%** | **n** | **%** |
| **POST-PREVENTIVE CHEMOTHERAPY INITIATION** | | | | | | | | | | | | | | | | | | |
| [1] | Davao Del Norte | 4-5 schools per mun 2 mun | Pre-SAC | 2013 | KK | 281 | 11 | 3.9 | 4 | 1.4 | 11 | 3.9 | 0 | 0.0 |  | |  | |
|  | Iloilo City |  |  | 2014 |  | 121 | 31 | 25.6 | 19 | 15.7 | 26 | 21.5 | 8 | 6.6 |  |  |  |  |
|  | Negros Occidental |  |  |  |  | 134 | 21 | 15.7 | 8 | 6.0 | 22 | 16.4 | 0 | 0.0 |  |  |  |  |
|  | Iloilo |  |  |  |  | 191 | 33 | 17.3 | 13 | 6.8 | 22 | 11.5 | 2 | 1.0 |  |  |  |  |
|  | Cavite |  |  |  |  | 219 | 67 | 30.6 | 45 | 20.5 | 50 | 22.8 | 21 | 9.6 |  |  |  |  |
|  | Guimaras |  |  |  |  | 330 | 44 | 13.3 | 20 | 6.1 | 55 | 16.7 | 6 | 1.8 |  |  |  |  |
|  | Lapu Lapu City |  |  | 2015 |  | 456 | 124 | 27.2 | 58 | 12.7 | 23 | 5.0 | 0 | 0.0 |  |  |  |  |
| [2] | Laguna | 10 schools, 10 mun | SAC | 2014 | KK, qPCR | 263 | 178 | 67.7 |  | | 141 | 53.6 |  | | 18 | 6.8 |  |  |
| [3] | Lapu Lapu City | 5 brgy, 1 city | Pre-SAC | 2017 | KK | 729 | 175 | 24.0 | 101 | 13.9 | 33 | 4.5 | 1 | 0.1 |  | |  |  |
| [4] | Masbate | 39 brgy, 4 mun | Pre-SAC | 2015 | KK | 1224 | 722 | 59.0 | 461 | 37.7 | 661 | 54.0 | 188 | 15.4 | 18 | 1.5 |  |  |
| [5] | Southern Leyte | 4 brgy | SAC, Pre-SAC | 2013 | KK/KT | 341 | 54 | 15.8 |  | | 68 | 19.9 |  | | 4 | 1.2 |  |  |
|  |  |  |  |  | KK | 316 |  | | 23 | 7.3 |  | | 5 | 1.6 |  | | 0 | 0.0 |
| [6] | Baguio City | 35 secondary schools, 16 local health units | Female highschool students | 2011 | KK/KT | 152 | 4 | 2.6 |  | | 17 | 11.2 |  | | 1 | 0.7 |  | |
|  | Cavite |  |  |  |  | 483 | 72 | 14.9 |  |  | 92 | 19.0 |  |  | 0 | 0.0 |  |  |
|  | Cebu City |  |  |  |  | 500 | 76 | 15.2 |  |  | 61 | 12.2 |  |  | 12 | 2.4 |  |  |
|  | Leyte |  |  |  |  | 445 | 159 | 35.7 |  |  | 222 | 49.9 |  |  | 29 | 6.5 |  |  |
|  | Davao City |  |  |  |  | 361 | 42 | 11.6 |  |  | 43 | 11.9 |  |  | 0 | 0.0 |  |  |
|  | Zamboanga Del Norte |  |  |  |  | 526 | 49 | 9.3 |  |  | 68 | 12.9 |  |  | 26 | 4.9 |  |  |
|  | Baguio City |  | Pregnant women |  |  | 228 | 7 | 3.1 |  |  | 24 | 10.5 |  |  | 4 | 1.8 |  |  |
|  | Cavite |  |  |  |  | 254 | 50 | 19.7 |  |  | 61 | 24.0 |  |  | 9 | 3.5 |  |  |
|  | Cebu City |  |  |  |  | 250 | 57 | 22.8 |  |  | 18 | 7.2 |  |  | 6 | 2.4 |  |  |
|  | Leyte |  |  |  |  | 182 | 97 | 53.3 |  |  | 115 | 63.2 |  |  | 31 | 17.0 |  |  |
|  | Davao City |  |  |  |  | 288 | 30 | 10.4 |  |  | 35 | 12.2 |  |  | 4 | 1.4 |  |  |
|  | Zamboanga Del Norte |  |  |  |  | 272 | 36 | 13.2 |  |  | 47 | 17.3 |  |  | 27 | 9.9 |  |  |
|  | Baguio City |  | Female highschool students |  | KK | 152 |  | | 0 | 0.0 |  | | 1 | 0.7 |  | | 0 | 0.0 |
|  | Cavite |  |  |  |  | 477 |  |  | 27 | 5.7 |  |  | 9 | 1.9 |  |  | 0 | 0.0 |
|  | Cebu City |  |  |  |  | 499 |  |  | 21 | 4.2 |  |  | 2 | 0.4 |  |  | 1 | 0.2 |
|  | Leyte |  |  |  |  | 443 |  |  | 75 | 16.9 |  |  | 48 | 10.8 |  |  | 2 | 0.5 |
|  | Davao City |  |  |  |  | 353 |  |  | 19 | 5.4 |  |  | 6 | 1.7 |  |  | 0 | 0.0 |
|  | Zamboanga Del Norte |  |  |  |  | 525 |  |  | 13 | 2.5 |  |  | 8 | 1.5 |  |  | 0 | 0.0 |
|  | Baguio City |  | Pregnant women |  |  | 228 |  |  | 1 | 0.4 |  |  | 1 | 0.4 |  |  | 0 | 0.0 |
|  | Cavite |  |  |  |  | 246 |  |  | 22 | 8.9 |  |  | 11 | 4.5 |  |  | 0 | 0.0 |
|  | Cebu City |  |  |  |  | 250 |  |  | 16 | 6.4 |  |  | 0 | 0.0 |  |  | 0 | 0.0 |
|  | Leyte |  |  |  |  | 182 |  |  | 57 | 31.3 |  |  | 45 | 24.7 |  |  | 0 | 0.0 |
|  | Davao City |  |  |  |  | 278 |  |  | 6 | 2.2 |  |  | 0 | 0.0 |  |  | 0 | 0.0 |
|  | Zamboanga Del Norte |  |  |  |  | 272 |  |  | 19 | 7.0 |  |  | 10 | 3.7 |  |  | 3 | 1.1 |
| [7] | Pampanga | 1 village, 1 mun | SAC | 2011 | KK | 195 | 164 | 84.1 | 129 | 66.2 | 184 | 94.4 | 95 | 48.7 | 42 | 21.5 | 4 | 2.1 |
| [8] | Aklan | 4-5 schools per district, 2 districts | SAC | 2007 | KK | 476 | 249 | 52.3 |  | | 305 | 64.1 |  | | 3 | 0.6 |  | |
|  | Antique |  |  |  |  | 397 | 149 | 37.5 |  |  | 272 | 68.5 |  |  | 20 | 5.0 |  |  |
|  | Capiz |  |  |  |  | 357 | 104 | 29.1 |  |  | 208 | 58.3 |  |  | 1 | 0.3 |  |  |
|  | Aklan |  |  | 2009 |  | 430 | 195 | 45.3 |  |  | 239 | 55.6 |  |  | 0 | 0.0 |  |  |
|  | Antique |  |  |  |  | 390 | 54 | 13.8 |  |  | 149 | 38.2 |  |  | 9 | 2.3 |  |  |
|  | Capiz |  |  |  |  | 529 | 93 | 17.6 |  |  | 228 | 43.1 |  |  | 16 | 3.0 |  |  |
|  | Aklan |  |  | 2011 |  | 418 | 87 | 20.8 |  |  | 199 | 47.6 |  |  | 44 | 10.5 |  |  |
|  | Antique |  |  |  |  | 372 | 59 | 15.9 |  |  | 146 | 39.2 |  |  | 0 | 0.0 |  |  |
|  | Capiz |  |  |  |  | 421 | 62 | 14.7 |  |  | 125 | 29.7 |  |  | 0 | 0.0 |  |  |
| [9] | Guimaras | 4 secondary schools, 2 mun | SAC | 2012 | KK/KT | 219 | 37 | 16.9 |  |  | 30 | 13.7 |  |  | 0 | 0.0 |  |  |
|  | Cavite | 8 secondary schools, 4 mun/cities |  | 2011 | KK/KT | 414 | 88 | 21.3 |  |  | 106 | 25.6 |  |  | 1 | 0.2 |  |  |
|  | Guimaras | 4 secondary schools, 2 mun |  | 2012 | KK | 212 |  | | 3 | 1.4 |  | | 1 | 0.5 |  | | 0 | 0.0 |
|  | Cavite | 8 secondary schools, 4 mun/cities |  | 2011 | KK | 396 |  |  | 38 | 9.6 |  |  | 19 | 4.8 |  |  | 0 | 0.0 |
| [10] | Laguna | 1 school, 1 mun | SAC | 2012 | FECT | 70 | 25 | 35.7 |  | | 34 | 48.6 |  | |  | |  | |
| [11] | Davao Del Norte | 11 brgy, 4 mun | IP and non-IP children | 2009 | KK/KT | 572 | 115 | 20.1 |  |  | 67 | 11.7 |  |  | 68 | 11.9 |  |  |
|  |  |  |  |  | KK | 510 |  | | 29 | 5.7 |  | | 1 | 0.2 |  | | 1 | 0.2 |
| [12] | Bukidnon | 5 schools | SAC | 2009 | DFS | 418 | 6 | 1.4 |  | | 1 | 0.2 |  | | 4 | 1.0 |  | |
| [13] | Cebu City | 10 schools, 2 districts | SAC | 2007 | KK | 536 | 236 | 44.0 | 133 | 24.8 | 205 | 38.2 | 24 | 4.5 |  | |  |  |
| [14] | Visayas | 55 brgy, 11 provinces | GP | 2007 | KK | 6321 | 2187 | 34.6 |  | | 2901 | 45.9 |  | | 499 | 7.9 |  |  |
| [15] | Pasay City | 1 school, 1 city | SAC | 1983 | FECT | 320 | 237 | 74.0 |  |  |  |  |  |  |  |  |  |  |
| [16] | Oriental Mindoro | 1 brgy, 1 mun | GP | 1982 | Stool exam | 623 | 62 | 10.0 |  |  | 174 | 27.9 |  |  | 33 | 5.3 |  |  |
| [17] | Ilocos Sur | 1 brgy, 1 mun | GP | 1975 | DFS,  FECT | 445 | 13 | 3.0 |  |  | 40 | 9.0 |  |  | 9 | 2.0 |  |  |
| **PRE-PREVENTIVE CHEMOTHERAPY INITIATION** | | | | | | | | | | | | | | | | | | |
| [18] | Benguet | 10 schools, 1 brgy | Pre-SAC, SAC |  | KK | 428 | 9 | 2.1 |  | | 1 | 0.2 |  | |  | |  | |
| [13] | Cebu City | 10 schools, 2 districts | SAC | 2006 | KK | 551 | 321 | 58.3 | 224 | 40.7 | 302 | 54.8 | 108 | 19.6 | 1 | 0.2 |  |  |
| [19] | Cebu | 8 villages, 2 mun (2,6) | Pre-SAC, SAC | 2002 | Stool  exam | 1990 | 1039 | 52.2 |  | | 335 | 16.8 |  | | 72 | 3.6 |  | |
| [14] | Mindanao | 110 brgy, 22 provinces | GP | 2005 | KK | 21390 | 4428 | 20.7 |  |  | 3487 | 16.3 |  |  | 2438 | 11.4 |  | |
| [20] | Quezon City | 4-6 schools per district, 2 districts | Pre-SAC, SAC | 2000 | KK | 310 | 89 | 28.7 | 46 | 14.8 | 118 | 38.1 | 31 | 10.0 | 21 | 6.8 |  |  |
|  | Pangasinan |  |  |  |  | 204 | 106 | 52.0 | 64 | 31.4 | 124 | 60.8 | 30 | 14.7 | 4 | 2.0 |  |  |
|  | Nueva Ecija |  |  |  |  | 310 | 199 | 64.2 | 144 | 46.5 | 187 | 60.3 | 65 | 21.0 | 2 | 0.6 |  |  |
|  | Cavite |  |  |  |  | 312 | 133 | 42.6 | 100 | 32.1 | 189 | 60.6 | 110 | 35.3 | 16 | 5.1 |  |  |
|  | Cebu City |  |  |  |  | 368 | 208 | 56.5 | 119 | 32.3 | 195 | 53.0 | 72 | 19.6 | 32 | 8.7 |  |  |
|  | Davao City |  |  |  |  | 367 | 132 | 36.0 | 75 | 20.4 | 165 | 45.0 | 41 | 11.2 | 13 | 3.5 |  |  |
| [21] | Metro Manila | 11 institutions, 3 communities | Street children | 2002 | FECT | 172 | 62 | 36.0 |  | | 77 | 44.8 |  | | 12 | 7.0 |  |  |
| [22] | Capiz | 1 city | GP | 2002 | FECT | 301 | 154 | 51.2 |  |  | 83 | 27.6 |  |  | 24 | 8.0 |  |  |
| [23] | Laguna | 1 mun | SAC |  | KK | 77 | 31 | 40.3 |  |  | 55 | 71.4 |  |  |  |  |  |  |
| [24] | Albay | 1 city | Pre-SAC, SAC, adult | 1998 | FECT | 64 | 26 | 40.6 |  |  | 33 | 51.6 |  |  | 15 | 23.4 |  |  |
| [25] | Laguna | 1 mun | Females age 15 to 55 years old |  | KK | 32 | 2 | 6.3 |  |  | 6 | 18.8 |  |  |  |  |  |  |
| [26] | Pampanga | 1 preschool | Pre-SAC |  | Stool  exam | 102 | 3 | 2.9 |  |  | 2 | 2.0 |  |  |  |  |  |  |
| [27] | Manila | 1 brgy, 1 mun | Pre-SAC, SAC | 1988 | KK, SAF | 238 | 191 | 80.3 |  |  | 220 | 92.4 |  |  | 23 | 9.7 |  |  |
| [28] | Palawan | 1 brgy,1 city | GP | 1986 | DFS, FECT | 365 | 127 | 34.8 |  |  | 92 | 25.2 |  |  | 127 | 34.8 |  |  |
| [29] | Cebu | 1 brgy, 1 mun | Children in the orphanage and milk recipients within the community |  | KK | 109 | 37 | 33.9 |  |  |  |  |  |  |  |  |  |  |
| [30] | Leyte | 1 brgy, 1 mun | GP |  | KK | 323 | 265 | 82.0 |  |  | 245 | 76.0 |  |  | 126 | 39.0 |  |  |
| [16] | Oriental Mindoro | 2 brgy, 1 mun | GP | 1979 | Stool  exam | 728 | 521 | 71.6 |  |  | 584 | 80.2 |  |  | 161 | 22.1 |  |  |
| [31] | Laguna | 1 brgy, 1 mun | SAC | 1981 | DFS | 38 | 37 | 97.4 |  |  | 25 | 65.8 |  |  | 3 | 7.9 |  |  |
| [32] | Leyte | 1 brgy, 1 mun | GP | 1977 | FECT | 204 | 151 | 74.0 |  |  | 194 | 95.0 |  |  | 24 | 12.0 |  |  |
|  | Manila | 1 brgy, 1 city | GP | 1977 | FECT | 150 | 48 | 32.0 |  |  | 114 | 76.0 |  |  | 1 | 0.7 |  |  |
| [17] | Ilocos Sur | 1 brgy, 1 mun | GP | 1974 | DFS,  FECT | 440 | 343 | 78.0 |  |  | 387 | 88.0 |  |  | 136 | 31.0 |  |  |
| [33] | Leyte |  | GP | 1974 | FECT | 606 | 512 | 84.5 |  |  |  |  |  |  |  |  |  |  |
| [34] | Rizal | 12 brgy, 2 mun | SAC | 1971 | KT,  DFS,  FECT | 2083 | 1914 | 91.9 |  |  | 1947 | 93.5 |  |  | 157 | 7.5 |  |  |
| [35] | Laguna | 5 brgy, 1 mun | GP | 1969 | KT | 761 | 605 | 79.5 |  |  |  |  |  |  |  |  |  |  |
| [36] | Manila | 1 brgy, 1 city | GP | 1969 | DFS | 294 | 179 | 60.9 |  |  | 145 | 49.3 |  |  | 3 | 1.0 |  |  |
| [37] | Palawan | 1 mun | SAC, adults |  | MIFC | 306 | 171 | 56.0 |  |  | 187 | 61.0 |  |  | 122 | 40.0 |  |  |
| [38] | Manila & Rizal | 3 schools | SAC | 1972 | DFS | 226 | 156 | 69.0 |  |  | 131 | 58.0 |  |  | 7 | 3.0 |  |  |
| [39] | PGH | 1 hospital | Chinese and Filipino food handlers | 1946 | Stool  exam | 3862 | 2292 | 59.3 |  |  | 930 | 24.1 |  |  | 255 | 6.6 |  |  |
| [40] | Isabela | 1 military camp | Soldiers | 2002 | FECT | 207 | 21 | 10.1 |  |  | 31 | 15.0 |  |  | 97 | 46.9 |  |  |
| [41] | Sorsogon | 1 brgy, 1 mun | GP | 1977 | Stool  exam | 361 | 296 | 82.0 |  |  | 335 | 92.8 |  |  | 263 | 72.9 |  |  |

Cluster - sampling cluster, pop - population examined, year - year of data collection, N - total number of participants examined/tested, n - number of participants positive, MHI - moderate to heavy intensity

**References**

1. delos Trinos JPCR, Belizario VY, Sison OT, Erasmo JN, Te MJ, Modequillo MC. Child development center-based sentinel surveillance of soil-transmitted helminthiases in preschool-age children in selected local government units in the Philippines. Acta Tropica. 2019;194:100-5.

2. Mationg MLS, Gordon CA, Tallo VL, Olveda RM, Alday PP, Renosa MDC, et al. Status of soil-transmitted helminth infections in schoolchildren in Laguna Province, the Philippines: Determined by parasitological and molecular diagnostic techniques. Plos Neglected Tropical Diseases. 2017;11(11):16.

3. Belizario VY. War on Worms Lapu-Lapu City Follow-Up Parasitological Assessment. 2017.

4. Belizario VY, Ng JV, Amarillo MLE, delos Trinos JPCR, Reyes MR, Fudalan O. High burden of soil-transmitted helminthiases in preschool-age children in masbate: A decade of implementation of the integrated helminth control program in the Philippines. Se Asian J Trop Med. 2016;47(4):667-79.

5. Belizario VY, Jr., Liwanag HJ, Naig JR, Chua PL, Madamba MI, Dahildahil RO. Parasitological and nutritional status of school-age and preschool-age children in four villages in Southern Leyte, Philippines: Lessons for monitoring the outcome of Community-Led Total Sanitation. Acta Trop. 2015;141(Pt A):16-24.

6. Belizario VJ TF, De Leon WU, Naig JRA. Improving Current Helminth Control Strategies: Lessons from a Baseline Prevalence Survey of Soil-Transmitted Helminth Infections in Adolescent Females and Pregnant Women in Selected Local Government Units in the Philippines. Acta Med Philipp. 2015;49(3): 5-11.

7. Ng JV, Vicente Y. Belizario J, Claveria FG. Determination of soil-transmitted helminth infection and its association with hemoglobin levels among Aeta schoolchildren of Katutubo Village in Planas, Porac, Pampanga Philippine Science Letters. 2014;7(1):73-80.

8. Belizario VY, Totanes FI, de Leon WU, Matias KM. School-based control of soil-transmitted helminthiasis in western Visayas, Philippines. The Southeast Asian journal of tropical medicine and public health. 2014;45(3):556-67.

9. Belizario Jr V, Chua PL, Liwanag HJ, Naig JR, Erfe JM. Soil-transmitted helminthiases in secondary school students in selected sites in two provinces in the Philippines: Policy implications. J Trop Pediatr. 2014;60(4):303-7.

10. Horiuchi S, Paller VG, Uga S. Soil contamination by parasite eggs in rural village in the Philippines. Trop. 2013;30(3):495-503.

11. Belizario VY, Jr., Totanes FIG, Leon WUd, Lumampao YF, Ciro RNT. Soil-transmitted helminth and other intestinal parasitic infections among school children in indigenous people communities in Davao del Norte, Philippines. (Special Issue: The diagnostics and control of neglected tropical helminth diseases.). Acta Trop. 2011;120(Supplement 1):S12-S8.

12. Batbatan CG. Prevalence of intestinal helminthic infections and assoiciated socio-cultural variables among schooling children of the public elementary schools of Maramag, Bukdinon. Central Mindanao University; 2009.

13. Belizario Jr VY, Plan AO, De Leon WU, Totañes FIG, Ciro RNT. Impact of a local government unit supported school-based initiative for control of intestinal helminth infections. Acta Med Philippina. 2011;45(2):18-23.

14. Leonardo LR, Rivera P, Saniel O, Villacorte E, Crisostomo B, Hernandez L, et al. Prevalence survey of schistosomiasis in Mindanao and the Visayas, The Philippines. Parasitol Int. 2008;57(3):246-51.

15. Cabrera BD. Reinfection and infection rates of ascariasis in relation to seasonal variation in the Philippines. The Southeast Asian journal of tropical medicine and public health. 1984;15(3):394-401.

16. Cabrera BD, Cruz AC. A comparative study on the effect of mass treatment of the entire community and selective treatment of children on the total prevalence of soil-transmitted helminthiasis in two communities, Mindoro, Philippines. Transactions of the National Academy of Science and Technology. 1983;5(6):97-124.

17. Banzon TC, Singson CN, Cross JH. Mebendazole treatment for intestinal nematodes in a Philippine barrio. [not specified]. Journal of the Philippine Islands Medical Association. 1976;52(7/8):239-43.

18. Abner O. Lawangen MCS, Danesto B. Anacio, Junelyn T. Tomin. Epidemiology of Soil-Transmitted Helminth Parasitism Among Schoolchildren in Tublay, Benguet. Tangkoyob: University of the Cordilleras Multidisciplinary Research Journal. 2012;6(1):15.

19. Tengco LW, Rayco-Solon P, Solon FS, Solon JA, Sarol JN. Determinants of Anemia among Preschool Children in the Philippines. J Am Coll Nutr. 2008;27(2):229-43.

20. Vicente Y. Belizario, Winifreda U. de Leon, Wambangco MAL, Esparar DG. Baseline assessment of intestinal parasitism in selected public elementary schools in Luzon, Visayas and Mindanao. Acta Medica Philippina. 2005;39(2):11-21.

21. Baldo ET, Belizario VY, De Leon WU, Kong HH, Chung DI. Infection status of intestinal parasites in children living in residential institutions in Metro Manila, the Philippines. The Korean journal of parasitology. 2004;42(2):67-70.

22. Kim BJ, Ock MS, Chung DI, Yong TS, Lee KJ. The intestinal parasite infection status of inhabitants in the Roxas city, The Philippines. The Korean journal of parasitology. 2003;41(2):113-5.

23. Yamamoto R, Nagai N, Kawabata M, Ubas-De Leon W, Ninomiya R, Koizumi N. Effect of intestinal helminthiasis on nutritional status of schoolchildren. Southeast Asian Journal of Tropical Medicine and Public Health. 2000;31(4):755-61.

24. Lee KJ, Ahn YK, Yong TS. A small-scale survey of intestinal parasite infections among children and adolescents in Legaspi city, the Philippines. The Korean journal of parasitology. 2000;38(3):183-5.

25. Ryoji Y, Nobuhiko N, Ubas-de Leon W, Naoko K. The relation between serum fatty acids and soil-transmitted helminthiasis in the Philippines. Southeast Asian Journal of Tropical Medicine and Public Health. 1997;28(2):329-34.

26. Laws HF, 2nd, Enriquez M. The prevalence of parasitism in preschool Americans in the Philippines. Military Medicine. 1990;155(12):585-7.

27. Auer C. Health status of children living in a squatter area of Manila, Philippines, with particular emphasis on intestinal parasitoses. The Southeast Asian journal of tropical medicine and public health. 1990;21(2):289-300.

28. Oberst RB, Alquiza LM. Survey of intestinal parasites on Palawan, Philippines. The Southeast Asian journal of tropical medicine and public health. 1987;18(2):197-201.

29. Fuentes CC. Double blind randomized study on the antihelminthic effect ofipil-ipil seed powder against Ascaris lumbricoides. Cebu Doctors' Proceedings. 1987;5(1):18-26.

30. Olveda RM, Icatlo FC, Jr., Libranda BD, Fevidal P, Jr., Domingo EO. A community-based clinical trial of albendazole in Leyte, Philippines. Philippine Journal of Internal Medicine. 1983;21(3):126-33.

31. Tomita M, Nakagawa T, Caragay R. The survey of parasitosis (I) in Bay (Philippines). Kobe Journal of Medical Sciences. 1982;28(1):1-5.

32. Cabrera BD, Sy FS. Oxantel-pyrantel in various regimens for the treatment of soil transmitted helminthiasis in rural and urban communities. Drugs. 1978;15(Suppl. 1):78-85.

33. Cabrera BD, Arambulo PV, III, Portillo GP. Ascariasis control and/or eradication in a rural community in the Philippines. Southeast Asian Journal of Tropical Medicine and Public Health. 1975;6(4):510-8.

34. Jueco NL, Palaypay MA, Aceremo L. Prevalence and intensity of intestinal parasitism on Talim Island, Binangonan, Rizal, Philippines. Southeast Asian J Trop Med Public Health. 1973;4(4):582-7.

35. Jueco NL, Cabrera BD. Reinfection rates of successfully treated cases of ascariasis in Victoria, Laguna. [not specified]. Journal of the Philippine Islands Medical Association. 1971;47(9):449-54.

36. Celis GR, Viloria EV. A parasitological survey of Leveriza, Malate, Manila. [not specified]. Acta Medica Philippina. 1970;6(3):95-101.

37. Kuntz R. Intestinal Parasites of Man in Palawan, Republic of the Philippines. Journal of the Philippine Islands Medical Association. 1963;39(7):590-600.

38. Villanueva N MA, De Castro CR, Rapanot N, Tantengco VO. Nutritional anemia in Filipino school children. J Philipp Med Assoc. 1973.

39. Calubaquib PB, Rolda H. The Incidence of Intestinal Parasitism among Food Handlers. [not specified]. Journal of the Philippine Islands Medical Association. 1947;23(4):149-52.

40. Vicente Y Belizario JMSV, Winifreda U de Leon, Donato G Esparar, Mark Phili Bugayong. Hookworm in the military: A parasitologic survey of military and para-military personnel in a Philippine military camp in Northern Luzon. Philippine Journal of Internal Medicine. 2005;43(4):169-74.

41. F Valeza, Cabrera B. The reinfection rate of soil-transmitted helminth in the pilot areas after treatment. Asian Parasite Control Organization [APCO]: Collec.
