# Supplementary material for: Prevalence of soil-transmitted helminth infections, schistosomiasis, and lymphatic filariasis before and after preventive chemotherapy initiation in the Philippines: A systematic review and meta-analysis
Source: PLoS Negl Trop Dis. 2021 Dec 20;15(12):e0010026. doi: 10.1371/journal.pntd.0010026 (PMC8722724; doi:10.1371/journal.pntd.0010026)
Supplement: S4 Table — (DOCX) [file pntd.0010026.s006.docx]

**S4 Table. Risk factors for STH infections**

| **Study** | **Risk factors** | **Relative Risk** | **95% CI** |
| --- | --- | --- | --- |
| [1] | Male | Reference |  |
|  | Female | 0.59 | (0.51-0.69) |
|  | None/preschool | Reference |  |
|  | Elementary | 1.90 | (1.40-2.56) |
|  | High school/vocational | 1.55 | (1.12-2.13) |
|  | College/post-graduate | 0.72 | 0.49-1.05 |
|  | Do not go to river | Reference |  |
|  | Go to river | 1.44 | (1.19-1.75) |
|  | Wealthy | 0.67 | (0.56-0.82) |
|  | Medium | Reference |  |
|  | Poor | 1.93 | (1.44-2.59) |
| [2] | Wealth | 0.81 | (0.71-0.92) |
|  | Schistosomiasis | 2.39 | (1.09-5.21) |
|  | At least one child has STH | 4.56 | (2.17-9.56) |

**References**

1. Ross AG, Olveda RM, McManus DP, Harn DA, Chy D, Li Y, et al. Risk factors for human helminthiases in rural Philippines. Int J Infect Dis. 2017;54:150-5.

2. Liwanag HJ, Uy J, Bataller R, Gatchalian JR, De La Calzada B, Uy JA, et al. Soil-transmitted helminthiasis and schistosomiasis in children of poor families in leyte, Philippines: Lessons for disease prevention and control. J Trop Pediatr. 2017;63(5):335-45.
