# Supplementary material for: Prevalence of soil-transmitted helminth infections, schistosomiasis, and lymphatic filariasis before and after preventive chemotherapy initiation in the Philippines: A systematic review and meta-analysis
Source: PLoS Negl Trop Dis. 2021 Dec 20;15(12):e0010026. doi: 10.1371/journal.pntd.0010026 (PMC8722724; doi:10.1371/journal.pntd.0010026)
Supplement: S5 Table — (DOCX) [file pntd.0010026.s007.docx]

**S5 Table. Studies which reported schistosomiasis prevalence only**

| **Reference** | **Site (Province/Highly-urbanised cities)** | **Clusters** | **Pop** | **Year** | **Test** | **N** | ***Schistosoma*** | | **MHI *Schistosoma*** | |
| --- | --- | --- | --- | --- | --- | --- | --- | --- | --- | --- |
|  |  |  |  |  |  |  | **n** | **%** | **n** | **%** |
| **POST-PREVENTIVE CHEMOTHERAPY INITIATION** | | | | | | | | | | |
| [1] | Northern Samar | 18 brgy, 2 mun | GP | 2015 | KK | 412 | 108 | 26.2 | 4 | 1.0 |
| [2] | Northern Samar | 18 brgy, 2 mun | GP | 2015 | qPCR | 412 | 307 | 74.5 |  | |
| [3] | Agusan Del Sur | 2 mun | SAC | 2013 | KK | 364 | 64 | 17.6 | 17 | 4.7 |
|  |  |  |  |  | FECT | 364 | 9 | 2.5 |  | |
|  |  |  |  |  | COPT | 364 | 99 | 27.2 |  |  |
|  |  |  |  |  | ELISA Ab | 364 | 313 | 86.0 |  |  |
|  |  |  |  |  | ELISA Ag | 364 | 175 | 48.1 |  |  |
|  | Bohol And Zamboanga Del Norte | 2 mun | SAC | 2013 | KK | 748 | 0 | 0.0 | 0 | 0.0 |
|  |  |  |  |  | FECT | 748 | 1 | 0.1 |  | |
|  |  |  |  |  | COPT | 748 | 82 | 11.0 |  |  |
|  |  |  |  |  | ELISA Ab | 748 | 126 | 16.8 |  |  |
|  |  |  |  |  | ELISA Ag | 748 | 64 | 8.6 |  |  |
| [4] | Northern Samar | 18 brgy, 2 mun | GP | 2015 | KK | 412 | 108 | 26.2 |  |  |
|  |  |  |  |  | ddPCR  feces | 412 | 307 | 74.5 |  |  |
|  |  |  |  |  | ddPCR serum | 412 | 277 | 67.2 |  |  |
|  |  |  |  |  | ddPCR  urine | 412 | 196 | 47.6 |  |  |
|  |  |  |  |  | ddPCR  saliva | 412 | 105 | 25.5 |  |  |
| [5] | Northern Samar | 18 brgy, 2 mun | GP | 2012 | KK | 565 | 192 | 34.0 |  |  |
| [6] | Davao City | 4 schools, 2 districts | SAC | 2015 | KK | 363 | 0 | 0.0 |  |  |
|  | Davao Del Sur |  |  |  |  | 433 | 2 | 0.5 |  |  |
|  | Compostela Valley |  |  |  |  | 501 | 18 | 3.6 |  |  |
|  | Davao City |  |  |  | ELISA Ag | 363 | 18 | 5.0 |  |  |
|  | Davao Del Sur |  |  |  |  | 433 | 13 | 3.0 |  |  |
|  | Compostela Valley |  |  |  |  | 501 | 72 | 14.4 |  |  |
|  | Davao City |  |  |  | ELISA Ab | 363 | 125 | 34.4 |  |  |
|  | Davao Del Sur |  |  |  |  | 433 | 83 | 19.2 |  |  |
|  | Compostela Valley |  |  |  |  | 501 | 283 | 56.5 |  |  |
| [7] | Northern Samar | 6 brgy, 1 mun | GP | 2011 | qPCR | 560 | 505 | 90.2 |  |  |
|  |  |  |  |  | KK | 560 | 128 | 22.9 |  |  |
| [8] | Negros Occidental | 7 schools, 1 mun | SAC | 2010 | KK /KT | 365 | 1 | 0.3 |  |  |
|  |  |  |  | 2012 | KK /KT | 465 | 32 | 6.9 |  |  |
|  |  |  |  | 2010 | KK | 362 |  | | 0 | 0.0 |
|  |  |  |  | 2012 | KK | 455 |  |  | 6 | 1.3 |
| **PRE-PREVENTIVE CHEMOTHERAPY INITIATION** | | | | | | | | | | |
| [9] | Western Samar | 50 villages | GP | 2004 | KK | 5624 | 995 | 17.7 | 174 | 3.1 |
| [10] | Leyte | 1 district | GP | 1997 | KK, Ultrasound | 118 | 41 | 34.7 |  | |
| [11] | Bohol | 2 mun | GP | 1981 | KK | 2595 | 122 | 4.7 |  |  |
| [11] | Bohol | 2 mun | GP | 1982 |  | 3337 | 59 | 1.8 |  |  |
|  |  |  |  | 1983 |  | 2922 | 50 | 1.7 |  |  |
|  |  |  |  | 1984 |  | 3212 | 139 | 4.3 |  |  |
|  |  |  |  | 1985 |  | 3642 | 39 | 1.1 |  |  |
|  |  |  |  | 1986 |  | 2641 | 71 | 2.7 |  |  |
|  |  |  |  | 1987 |  | 5807 | 92 | 1.6 |  |  |
|  |  |  |  | 1988 |  | 4135 | 59 | 1.4 |  |  |
|  |  |  |  | 1989 |  | 6162 | 31 | 0.5 |  |  |
|  |  |  |  | 1990 |  | 7307 | 22 | 0.3 |  |  |
|  |  |  |  | 1991 |  | 9536 | 59 | 0.6 |  |  |
|  |  |  |  | 1992 |  | 8054 | 32 | 0.4 |  |  |
|  |  |  |  | 1993 |  | 6954 | 14 | 0.2 |  |  |
|  |  |  |  | 1994 |  | 8642 | 15 | 0.2 |  |  |
|  |  |  |  | 1995 |  | 8779 | 7 | 0.1 |  |  |
| [12] | Leyte | 3 brgy, 3 mun | GP | 1981 | KK | 2233 | 870 | 38.9 |  |  |
| [13] | Bohol | 1 brgy, 1 mun | GP | 1984 | COPT | 464 | 103 | 22.2 |  |  |
|  |  |  |  | 1986 |  | 473 | 66 | 14.0 |  |  |
|  |  | 1 brgy, 1 mun |  | 1982 |  | 389 | 83 | 21.3 |  |  |
|  |  |  |  | 1986 |  | 508 | 103 | 20.3 |  |  |
| [14] | Leyte | 9 schools, 1 mun | SAC | 1976 | MIFC,  COPT | 690 | 231 | 33.5 |  |  |
|  |  |  |  | 1977 |  | 741 | 253 | 34.1 |  |  |
|  |  |  |  | 1978 |  | 1515 | 718 | 47.4 |  |  |
|  |  |  |  | 1979 |  | 1439 | 412 | 28.6 |  |  |
|  |  |  |  | 1980 |  | 1261 | 551 | 43.7 |  |  |
|  |  |  |  | 1981 |  | 1154 | 330 | 28.6 |  |  |
|  |  |  |  | 1983 |  | 1704 | 450 | 26.4 |  |  |
|  |  |  |  | 1984 |  | 1283 | 314 | 24.5 |  |  |
| [15] | Leyte | 1 school, 1 mun | SAC | 1979 | Stool exam (unspecified) | 401 | 127 | 31.7 |  |  |
|  |  |  |  | 1980 |  | 373 | 71 | 19.0 |  |  |
|  |  |  |  | 1981 |  | 407 | 69 | 17.0 |  |  |
|  |  |  |  | 1982 |  | 294 | 39 | 13.2 |  |  |
|  |  |  |  | 1979 | ELISA (unspecified) | 410 | 251 | 61.2 |  |  |
|  |  |  |  | 1980 |  | 381 | 207 | 54.3 |  |  |
|  |  |  |  | 1981 |  | 413 | 210 | 50.8 |  |  |
|  |  |  |  | 1982 |  | 322 | 159 | 49.4 |  |  |
| [16] | Leyte | 9 schools, 1 mun | SAC | 1976 | COPT | 690 | 231 | 33.5 |  |  |
|  |  |  |  | 1977 |  | 741 | 253 | 34.1 |  |  |
|  |  |  |  | 1978 |  | 1515 | 718 | 47.4 |  |  |
|  |  |  |  | 1979 |  | 1439 | 412 | 28.6 |  |  |
|  |  |  |  | 1980 |  | 1261 | 551 | 43.7 |  |  |
|  |  |  |  | 1981 |  | 1154 | 330 | 28.6 |  |  |
| [17] | Leyte | 1 school, 1 mun | SAC | 1981 | MFCT | 598 | 170 | 28.4 |  |  |
|  |  |  |  |  | ELISA (unspecified) | 598 | 336 | 56.2 |  |  |
| [18] | Leyte | 3 brgy, 3 mun | GP | 1982 | KT | 2226 | 886 | 39.8 |  |  |
| [19] | Leyte | 9 schools, 1 mun | SAC | 1976 | MFCT | 690 | 231 | 33.5 |  |  |
|  |  |  |  | 1977 |  | 741 | 253 | 34.1 |  |  |
|  |  |  |  | 1978 |  | 1515 | 718 | 47.4 |  |  |
|  |  |  |  | 1979 |  | 1439 | 412 | 28.6 |  |  |
|  |  |  |  | 1980 |  | 1261 | 551 | 43.7 |  |  |
|  |  | 1 school, 1 mun |  | 1976 |  | 151 | 35 | 23.2 |  |  |
|  |  |  |  | 1977 |  | 113 | 22 | 19.5 |  |  |
|  |  |  |  | 1978 |  | 382 | 170 | 44.5 |  |  |
|  |  |  |  | 1979 |  | 370 | 103 | 27.8 |  |  |
|  |  |  |  | 1980 |  | 268 | 90 | 33.6 |  |  |
| [20] | Leyte | 1 brgy, 1 mun | GP | 1979 | KK | 1010 | 354 | 35.0 |  |  |
| [21] | Sorsogon | 2 brgy, 1 mun | GP | 1978 | KT, MIFC | 755 | 326 | 43.2 |  |  |
| [22] | Leyte | 9 schools, 1 mun | SAC | 1975 | MFCT | 552 | 186 | 33.7 |  |  |
|  |  |  |  | 1975 | COPT | 624 | 481 | 77.1 |  |  |
|  |  |  |  | 1976 | MFCT | 577 | 176 | 30.5 |  |  |
|  |  |  |  | 1976 | COPT | 673 | 142 | 21.1 |  |  |
|  |  |  |  | 1977 | MFCT | 554 | 148 | 26.7 |  |  |
|  |  |  |  | 1977 | COPT | 732 | 185 | 25.3 |  |  |
| [23] | Sorsogon | 1 mun | GP | 1977 | COPT | 237 | 29 | 12.2 |  |  |
|  |  |  |  |  | FECT | 564 | 16 | 2.8 |  |  |
|  |  |  |  |  | COPT | 280 | 35 | 12.5 |  |  |
|  |  |  |  |  | FECT | 655 | 19 | 2.9 |  |  |
| [24] | Leyte | 1 mun | GP | 1954 | Stool exam (unspecified) | 1356 | 528 | 38.9 |  |  |
|  |  |  |  | 1959 |  | 1621 | 519 | 32.0 |  |  |
|  |  |  | < 10 y/o | 1954 |  | 239 | 26 | 10.9 |  |  |
|  |  |  |  | 1959 |  | 332 | 46 | 13.9 |  |  |
| [25] Pesigan et al. 1958 | Leyte | 3 mun | GP | 1955 | MIFC, Stoll | 4079 | 1159 | 28.4 |  |  |

Cluster - sampling cluster, pop - population examined, year - year of data collection, N - total number of participants examined/tested, n - number of participants positive, MHI - moderate to heavy intensity

**References**

1. Cai PF, Weerakoon KG, Mu Y, Olveda RM, Ross AG, Olveda DU, et al. Comparison of Kato Katz, antibody-based ELISA and droplet digital PCR diagnosis of schistosomiasis japonica: Lessons learnt from a setting of low infection intensity. Plos Neglected Tropical Diseases. 2019;13(3):17.

2. Weerakoon KG, Gordon CA, Williams GM, Cai P, Gobert GN, Olveda RM, et al. Co-parasitism of intestinal protozoa and Schistosoma japonicum in a rural community in the Philippines. Infect. 2018;7 (1) (no pagination)(121):121.

3. Belizario V, Destura R, Gabunada RR, Petronio-Santos JA, de la Tonga A, Amarillo ML, et al. Evaluation of fecal and serological tests for the diagnosis of schistosomiasis in selected near-elimination and endemic areas in the philippines. Southeast Asian Journal of Tropical Medicine and Public Health. 2018;49(2):198-207.

4. Weerakoon KG, Gordon CA, Williams GM, Cai PF, Gobert GN, Olveda RM, et al. Droplet Digital PCR Diagnosis of Human Schistosomiasis: Parasite Cell-Free DNA Detection in Diverse Clinical Samples. J Infect Dis. 2017;216(12):1611-22.

5. Olveda DU, Inobaya M, Olveda RM, Vinluan ML, Ng SK, Weerakoon K, et al. Diagnosing schistosomiasis-induced liver morbidity: implications for global control. Int J Infect Dis. 2017;54:138-44.

6. Belizario V, Jr., Bungay AA, Su GS, de Veyra C, Lacuna JD. Assessment of three schistosomiasis endemic areas using kato-katz technique and elisa antigen and antibody tests. Southeast Asian Journal of Tropical Medicine and Public Health. 2016;47(4):638-50.

7. Gordon CA, Acosta LP, Gobert GN, Olveda RM, Ross AG, Williams GM, et al. Real-time PCR Demonstrates High Prevalence of Schistosoma japonicum in the Philippines: Implications for Surveillance and Control. Plos Neglected Tropical Diseases. 2015;9(1):14.

8. Belizario VY, Jr., Erfe JM, Naig J, Chua P. Evidence of increasing risk of schistosomiasis among school-age children in municipality of Calatrava, Province of Negros Occidental, Philippines. Asian Pacific Journal of Tropical Medicine. 2015;8(5):373-7.

9. Tarafder MR, Balolong E, Carabin H, Belisle P, Tallo V, Joseph L, et al. A cross-sectional study of the prevalence of intensity of infection with Schistosoma japonicum in 50 irrigated and rain-fed villages in Samar Province, the Philippines. BMC Public Health. 2006;6:10.

10. Kardorff R, Olveda RM, Acosta LP, Duebbelde UJ, Aligui GD, Alcorn NJ, et al. Hepatosplenic morbidity in schistosomiasis japonica: Evaluation with Doppler sonography. American Journal of Tropical Medicine and Hygiene. 1999;60(6):954-9.

11. Yasuraoka K, Blas BL, Matsuda H, Irie Y, Nihei N, Ohmae H, et al. Approaches to the elimination of schistosomiasis on Bohol Island, Philippines. Japanese Journal of Parasitology. 1996;45(5):391-9.

12. Olveda RM, Daniel BL, Ramirez BDL, Aligui GDL, Acosta LP, Fevidal P, et al. Schistosomiasis japonica in the Philippines: the long-term impact of population-based chemotherapy on infection, transmission, and morbidity. J Infect Dis. 1996;174(1):163-72.

13. Nakao M, Matsuda H, Tanaka H, Santos AT, Jr., Blas BL, Nakamura S. Negative conversion of COP (circumoval precipitation) reactions after selective mass chemotherapy on schistosomiasis japonica with praziquantel in Bohol, Philippines. Jpn J Exp Med. 1987;57(5):261-6.

14. Tanaka H, Blas BL, Nosenas JS, Matsuda H, Hayashi Y, Santos AT, Jr. Longitudinal study on Schistosoma japonicum infections in the Philippines. 4. Effect of mass-chemotherapy with praziquantel on incidence at Dagami, Leyte. Jpn J Exp Med. 1985;55(4):161-5.

15. Yogore MG, Lewert RM, Blas BL. SEROEPIDEMIOLOGY OF SCHISTOSOMIASIS-JAPONICA BY ELISA IN THE PHILIPPINES .3. SELECTIVE MASS CHEMOTHERAPY WITH PRAZIQUANTEL IN A CONTROL PROGRAM. American Journal of Tropical Medicine and Hygiene. 1984;33(5):882-90.

16. Tanaka H, Blas BL, Nosenas JS, Matsuda H, Hayashi Y, Santos AT, Jr. Epidemiology and transmission dynamics; evaluation of control measures by means of annual incidence of infection with Schistosoma japonicum among school children at Dagami, Leyte, Philippines. Southeast Asian Journal of Tropical Medicine and Public Health. 1984;15(4):480-1.

17. Yogore MG, Lewert RM, Blas BL. SEROEPIDEMIOLOGY OF SCHISTOSOMIASIS JAPONICA BY ELISA IN THE PHILIPPINES .1. UNDERESTIMATION BY STOOL EXAMINATION OF THE PREVALENCE OF INFECTION IN SCHOOL-CHILDREN. American Journal of Tropical Medicine and Hygiene. 1983;32(6):1322-34.

18. Tiu E, Fevidal Jr P, de Veyra Jr F, Icatlo Jr FC, Domingo EO. Relationship of prevalence and intensity of infection to morbidity in schistosomiasis japonica: A study of three communities in Leyte, Philippines. American Journal of Tropical Medicine and Hygiene. 1983;32(6):1312-21.

19. Tanaka H, Blas BL, Nosenas JS, Matsuda H, Ishige M, Kamiya H, et al. Longitudinal study on Schistosoma japonicum infections in the Philippines. 3. Incidence and environmental modification at Dagami, Leyte. Jpn J Exp Med. 1983;53(2):87-94.

20. Tiu E, Peters PA, Warren KS, Mahmoud AA, Houser HB. Morbidity in Schistosomiasis japonica in relation to intensity of infection: Study of a community in Leyte, Philippines. American Journal of Tropical Medicine and Hygiene. 1980;29(5):I.

21. World Health Organisation. QUANTITATIVE ASPECTS OF THE EPIDEMIOLOGY OF SCHISTOSOMA-JAPONICUM INFECTION IN A RURAL-COMMUNITY OF LUZON, PHILIPPINES. Bull World Health Organ. 1980;58(4):629-38.

22. Blas BL, Nosenas JS, Tanaka H, Matsudo O, Onodera N, Matsuda H, et al. Longitudinal study on Schistosoma japonicum infections in the Philippines. Incidence and prevalence among school children in Dagami Area, Leyte. Jpn J Exp Med. 1979;49(2):107-15.

23. Cabrera BD, Valeza F, Santos AT, Jr., Cruz I. Current status of schistosomiasis japonica in Sorsogon Province, Republic of the Philippines. Southeast Asian J Trop Med Public Health. 1978;9(1):86-92.

24. Pesigan TP, Hairston NG. The effect of snail control on the prevalence of Schistosoma japonicum infection in the Philippines. [not specified]. Bull World Health Organ. 1961;25(4/5):479-82.

25. Pesigan TP, Farooq M, Hairston NG, Jauregui JJ, Garcia EG, Santos AT, et al. Studies on Schistosoma japonicum infection in the Philippines. 1. General considerations and epidemiology. Bull World Health Organ. 1958;18(3):345-455.
