# Supplementary material for: Prevalence of soil-transmitted helminth infections, schistosomiasis, and lymphatic filariasis before and after preventive chemotherapy initiation in the Philippines: A systematic review and meta-analysis
Source: PLoS Negl Trop Dis. 2021 Dec 20;15(12):e0010026. doi: 10.1371/journal.pntd.0010026 (PMC8722724; doi:10.1371/journal.pntd.0010026)
Supplement: S6 Table — (DOCX) [file pntd.0010026.s008.docx]

**S6 Table. Risk factors for schistosomiasis**

| **Study** | **Risk factors** | **Relative Risk** | **95% CI** |
| --- | --- | --- | --- |
| [1] | Male | Reference |  |
|  | Female | 0.41 | (0.33-0.52) |
|  | <15 | Reference |  |
|  | 15-34 y/o | 1.66 | (1.15-2.39) |
|  | 35 and above | 1.21 | (0.81-1.80) |
|  | Unemployed/retired/housewife | Reference |  |
|  | Student | 2.29 | (1.69-3.28) |
|  | Farmer | 1.91 | (1.34-2.70) |
|  | Fishing | 3.42 | (1.56-7.49) |
|  | Professional/worker/other | 1.47 | (1.02-2.13) |
|  | None/preschool | Reference |  |
|  | Elementary | 3.12 | (1.98-4.90) |
|  | High-school/vocational | 2.23 | (1.36-3.67) |
|  | College/post-graduate | 0.65 | (0.35-1.23) |
|  | Do not go to river | Reference |  |
|  | Go to river | 1.93 | (1.47-2.54) |
|  | Wealthy | 0.48 | (0.37-0.63) |
|  | Medium | Reference |  |
|  | Poor | 1.70 | (1.17-2.46) |
|  | Barangay variance | 0.66 | (0.31-1.40) |
|  | Household variance | 1.48 | (1.04-2.12) |
| [2] | STH | 2.11 | (1.04-4.27) |
|  | At least one sibling has schistosomiasis | 5.78 | (3.10-10.80) |
|  | Family is enrolled in CCT program | 2.94 | (1.43-6.04) |
|  | Wealth index | 0.77 | (0.64-0.92) |
| [3] | Rain-fed villages | Reference |  |
|  | Irrigated villages | 1.41 | (0.50-3.21) |
|  | Works most of the time on a rice farm | Reference |  |
|  | Works sometimes on a rice farm | 1.32 | (0.82-2.02) |
|  | Works on a farm but never on a rice farm | 0.42 | (0.15-0.87) |
|  | Does not work on a farm | 0.55 | (0.30-0.90) |
|  | Does not work on a rice farm, may work on another type of farm | 0.52 | (0.37-0.72) |
|  | Male, <10 | Reference |  |
|  | Male, 10-16 year | 6.10 | (3.86-9.28) |
|  | Male, 16-40 | 8.76 | (6.03-12.47) |
|  | Male, >40 years | 3.53 | (2.37-5.15) |
|  | Female, <10 years | Reference |  |
|  | Female, 10-16 years | 8.59 | (4.74-14.28) |
|  | 16-40 years | 5.98 | (3.53-9.61) |
|  | >40 years | 3.46 | (2.02-5.55) |

**References**

1. Ross AG, Olveda RM, McManus DP, Harn DA, Chy D, Li Y, et al. Risk factors for human helminthiases in rural Philippines. Int J Infect Dis. 2017;54:150-5.

2. Liwanag HJ, Uy J, Bataller R, Gatchalian JR, De La Calzada B, Uy JA, et al. Soil-transmitted helminthiasis and schistosomiasis in children of poor families in leyte, Philippines: Lessons for disease prevention and control. J Trop Pediatr. 2017;63(5):335-45.

3. Tarafder MR, Balolong E, Carabin H, Belisle P, Tallo V, Joseph L, et al. A cross-sectional study of the prevalence of intensity of infection with Schistosoma japonicum in 50 irrigated and rain-fed villages in Samar Province, the Philippines. BMC Public Health. 2006;6:10.
