# Supplementary material for: Prevalence of soil-transmitted helminth infections, schistosomiasis, and lymphatic filariasis before and after preventive chemotherapy initiation in the Philippines: A systematic review and meta-analysis
Source: PLoS Negl Trop Dis. 2021 Dec 20;15(12):e0010026. doi: 10.1371/journal.pntd.0010026 (PMC8722724; doi:10.1371/journal.pntd.0010026)
Supplement: S7 Table — (DOCX) [file pntd.0010026.s009.docx]

**S7 Table. Studies which reported lymphatic filariasis prevalence only**

| **Reference** | **Site (Province/Highly-urbanised cities)** | **Clusters** | **Year** | **Test** | **N** | **n** | **Prev (%)** |
| --- | --- | --- | --- | --- | --- | --- | --- |
| [1] | Oriental Mindoro | 2 brgy, 1 mun |  | NBEG | 564 | 44 | 7.8 |
| [2] | Sorsogon | 1 brgy, 1 mun | 1994 | BEG | 54 | 3 | 5.6 |
|  |  |  |  | Micros. of membrane filtered blood | 54 | 4 | 7.4 |
|  |  |  |  | PCR | 54 | 8 | 14.8 |
| [3] | Marinduque | 3 brgy | 1990 | NBEG | 297 | 47 | 15.8 |
| [4] | Sorsogon | 2 brgy, 1 mun |  | NBEG | 262 | 82 | 31.3 |
| [5] | Mindoro | 6 mun | 1980 | NBEG | 972 | 136 | 14.0 |
| [6] | Sorsogon | 1 brgy, 1 mun | 1978 | NBEM | 192 | 31 | 16.1 |
|  | Sorsogon |  |  | Micros. blood film | 192 | 22 | 11.5 |
|  | Sorsogon |  |  | Micros. Millipore filter | 192 | 25 | 13.0 |
|  | Sorsogon |  |  | Micros. nucleopore filter | 192 | 30 | 15.6 |
| [7] | Agusan Del Norte | 2 brgy, 2 mun | 1978 | DBEG | 200 | 50 | 25.0 |
|  | Surigao Del Norte | 1 brgy, 1 mun |  |  | 83 | 16 | 19.3 |
| [8] | Catanduanes | 1 brgy, 1 mun |  | Micros. Millipore filter | 459 | 74 | 16.1 |
| [9] | Northern Samar |  | 1973 | BEG | 1859 | 65 | 3.5 |
| [10] | Eastern Samar | 22 brgy, 1 mun | 1971 | NBEG | 14543 | 743 | 5.1 |
| [11] | Sorsogon | 1 brgy, 1 mun | 1972 | NBEG | 757 | 86 | 11.4 |
| [12] | Palawan | 4 brgy, 2 mun | 1971 | NBEG | 248 | 37 | 14.9 |
| [13] | Sulu | 44 brgy, 9 mun | 1967 | NBEG | 3695 | 120 | 3.2 |
| [14] | Palawan | 88 brgy, 12 mun | 1965 | NBEG | 3726 | 388 | 10.4 |
| [15] | Palawan | 1 brgy, 1 mun |  | NBEG | 14 | 9 | 64.3 |
| [16] | Palawan | 29 brgy, 5 mun | 1964 | NBEG | 915 | 176 | 19.2 |
| [17] | Abra |  | 1963 | NBEG | 4337 | 0 | 0.0 |
|  | Agusan |  |  |  | 5487 | 129 | 2.4 |
|  | Albay |  |  |  | 1723 | 750 | 43.5 |
|  | Bataan |  |  |  | 1435 | 1 | 0.1 |
|  | Batangas |  |  |  | 1508 | 1 | 0.1 |
|  | Bohol |  |  |  | 5414 | 15 | 0.3 |
|  | Bulacan |  |  |  | 149 | 0 | 0.0 |
|  | Bukidnon |  |  |  | 3573 | 89 | 2.5 |
|  | Cagayan |  |  |  | 6294 | 0 | 0.0 |
|  | Camarines Norte |  |  |  | 1725 | 105 | 6.1 |
|  | Camarines Sur |  |  |  | 2389 | 183 | 7.7 |
|  | Catanduanes |  |  |  | 2252 | 77 | 3.4 |
|  | Cavite |  |  |  | 1209 | 1 | 0.1 |
|  | Cebu |  |  |  | 3766 | 1 | 0.0 |
|  | Cotabato |  |  |  | 6498 | 135 | 2.1 |
|  | Davao |  |  |  | 8423 | 173 | 2.1 |
|  | Ilocos Norte |  |  |  | 3373 | 0 | 0.0 |
|  | Ilocos Sur |  |  |  | 4909 | 0 | 0.0 |
|  | Isabela |  |  |  | 893 | 0 | 0.0 |
|  | Laguna |  |  |  | 938 | 0 | 0.0 |
|  | Lanao Del Norte |  |  |  | 3382 | 7 | 0.2 |
|  | Lanao Del Sur |  |  |  | 3617 | 69 | 1.9 |
|  | La Union |  |  |  | 2970 | 0 | 0.0 |
|  | Leyte |  |  |  | 16193 | 269 | 1.7 |
|  | Southern Leyte |  |  |  | 5555 | 105 | 1.9 |
|  | Masbate |  |  |  | 6894 | 206 | 3.0 |
|  | Misamis Occidental |  |  |  | 4364 | 6 | 0.1 |
|  | Misamis Oriental |  |  |  | 6751 | 165 | 2.4 |
|  | Mountain Province |  |  |  | 3790 | 5 | 0.1 |
|  | Negros Oriental |  |  |  | 1958 | 1 | 0.1 |
|  | Nueva Ecija |  |  |  | 2154 | 0 | 0.0 |
|  | Nueva Vizcaya |  |  |  | 1697 | 0 | 0.0 |
|  | Occidental Mindoro |  |  |  | 3751 | 9 | 0.2 |
|  | Oriental Mindoro |  |  |  | 4373 | 65 | 1.5 |
|  | Palawan |  |  |  | 14968 | 353 | 2.4 |
|  | Pampanga |  |  |  | 1400 | 0 | 0.0 |
|  | Pangasinan |  |  |  | 6511 | 0 | 0.0 |
|  | Quezon |  |  |  | 3043 | 26 | 0.9 |
|  | Rizal |  |  |  | 776 | 1 | 0.1 |
|  | Romblon |  |  |  | 2652 | 46 | 1.7 |
|  | Samar |  |  |  | 20922 | 845 | 4.0 |
|  | Sorsogon |  |  |  | 77650 | 6525 | 8.4 |
|  | Sulu |  |  |  | 982 | 100 | 10.2 |
|  | Surigao Del Norte |  |  |  | 4406 | 226 | 5.1 |
|  | Surigao Del Sur |  |  |  | 2413 | 84 | 3.5 |
|  | Tarlac |  |  |  | 1673 | 0 | 0.0 |
|  | Zambales |  |  |  | 2991 | 0 | 0.0 |
|  | Zamboanga Del Norte |  |  |  | 4528 | 36 | 0.8 |
|  | Zamboanga Del Sur |  |  |  | 4053 | 14 | 0.3 |
| [18] | Mountain Province | 26 brgy, 6 mun |  | NBEG | 280 | 30 | 10.7 |
| [19] | Sorsogon | 108 brgy, 9 mun | 1956 | BEG | 12758 | 1321 | 10.4 |

Cluster - sampling cluster, year - year of data collection, N - total number of participants examined/tested, n - number of participants positive, MHI - moderate to heavy intensity

**References**

1. Ramirez BL, Hernandez L, Alberto FF, Collins M, Nfonsam V, Punsalan T, et al. Contrasting Wuchereria bancrofti microfilaria rates in two Mangyan-populated Philippine villages. American Journal of Tropical Medicine and Hygiene. 2004;71(1):17-23.

2. Torres EP, Ramirez BL, Salazar F, Pasay MCJ, Alamares JG, Santiago ML, et al. Detection of bancroftian filariasis in human blood samples from Sorsogon province, the Philippines by polymerase chain reaction. Parasitol Res. 2001;87(8):677-9.

3. Go VM. Lymphatic filariasis in a recently described endemic area in Marinduque, Philippines. The Southeast Asian journal of tropical medicine and public health. 1993;24 Suppl 2:19-22.

4. Ishii A, Cabrera BD, Suguri S, Kobayashi M, Go TG, Valeza F. An epidemiological study of filariasis in Sorsogon province, Republic of the Philippines, with notes on experimental mosquito infection. Journal of Tropical Medicine and Hygiene. 1983;86(2):59-64.

5. Enarson DA, Enarson PM. Filariasis in an upland population in the Philippines. Tropical and Geographical Medicine. 1982;34(4):353-8.

6. Shibuya T, Tanaka H, Cabrera BD, Valesa FS, Instrella R, Go T, et al. Low density microfilaremia of Wuchereria bancrofti infections in pre- and post-treatment phases in the Philippines. Jpn J Exp Med. 1981;51(2):133-5.

7. Tanaka H, Nakai H, Omoto K, Shibuya T, Hirai M, Mercado AS, et al. The high prevalence of Wuchereria bancrofti infections in indigenous tribes in Northern Mindanao, Philippines. Jpn J Exp Med. 1980;50(2):85-9.

8. Grove DI, Valeza FS, Cabrera BD. Bancroftian filariasis in a Philippine village: clinical, parasitological, immunological, and social aspects. Bull World Health Organ. 1978;56(6):975-84.

9. Cross JH, Banzon T, Wheeling CH, Cometa H, Lien JC, Clarke R, et al. Biomedical survey in North Samar Province, Philippine Islands. Southeast Asian J Trop Med Public Health. 1977;8(4):464-75.

10. Wenceslao JM, Oban E, Cabrera BD. Eastern Samar, the fourth endemic focus for Malayan filariasis in the Republic of the Philippines. Southeast Asian Journal of Tropical Medicine and Public Health. 1972;3(4):552-61.

11. Cabrera BD, Valeza F. Filariasis in Sablayan Island, Juban, Sorsogon. Acta Medica Philippina. 1972;8(4):145-51.

12. Cabrera BD, Jueco NL. Filariasis survey among indigenous tribes of Palawan, Republic of the Philippines. Stheast Asian J. 1972;Trop. Med. Publ. Hlth. 3(1):31-9.

13. Cabrera BD, Cruz I. The second endemic locus for malayan filariasis in the repubUc of the Philippines. Acta Medphilipp. 1968;5(1):1-24.

14. Cabrera BD, Tamondong CT. Bancroftian and malayan filariasis in palawan. Extent and distribution. Acta Medica Philippina. 1966;3(1):20-36.

15. Rozeboom LE, Cabrera BD. FILARIASIS CAUSED BY WUCHERERIA-BANCROFTI IN PALAWAN, REPUBLIC-OF-THE-PHILIPPINES. Am J Epidemiol. 1965;81(2):216-21.

16. Rozeboom LE, Cabrera BD. FILARIASIS CAUSED BY BRUGIA-MALAYI IN THE REPUBLIC-OF-THE-PHILIPPINES. Am J Epidemiol. 1965;81(2):200-15.

17. Estrada JP, Basio DG. Filariasis in the Philippines. [not specified]. Journal of the Philippine Islands Medical Association. 1965;41(2):100-53.

18. Rozeboom LE, Cabrera BD. Filariasis in Mountain Province, Luzon, Republic of the Philippines. [not specified]. J Med Entomol. 1964;1(1):18-28.

19. Baisas FE. Notes on Philippine mosquitoes, XIX. The mosquito problem in the control of fllariasis in Sorsogon Province. [not specified]. Philippine Journal of Science. 1958;86(1):71-120.
