# Supplementary material for: Prevalence of soil-transmitted helminth infections, schistosomiasis, and lymphatic filariasis before and after preventive chemotherapy initiation in the Philippines: A systematic review and meta-analysis
Source: PLoS Negl Trop Dis. 2021 Dec 20;15(12):e0010026. doi: 10.1371/journal.pntd.0010026 (PMC8722724; doi:10.1371/journal.pntd.0010026)
Supplement: S8 Table — (DOCX) [file pntd.0010026.s010.docx]

**S8 Table. Categories of Lymphatic Filariasis-Endemic Provinces**

| **Ongoing MDA** | **Pre-TAS** | **Passed TAS 1** | **Passed TAS 2** | **Passed TAS 3** | |
| --- | --- | --- | --- | --- | --- |
| 1. Sultan Kudarat 2. Zamboanga del Norte | 1. Aklan | 1. Davao del Sur 2. Basilan 3. *Oriental Mindoro** 4. Antique 5. Lanao del Norte 6. Surigao del Norte | 1. Misamis Oriental 2. Sarangani 3. Quezon Province 4. Camarines Norte 5. Capiz 6. Sulu | 1. Agusan del Norte 2. Agusan del Sur 3. Albay 4. Biliran 5. Bukidnon 6. Camarines Sur 7. Catanduanes 8. Davao del Norte Compostela Valley 9. Davao Oriental 10. Dinagat Islands 11. Eastern Samar 12. Iloilo Province 13. Maguindanao 14. Marinduque 15. Masbate | 1. Misamis Occidental 2. Negros Oriental 3. North Cotabato 4. Northern Leyte 5. Northern Samar 6. Occidental Mindoro 7. Palawan 8. Romblon 9. Sorsogon 10. South Cotabato 11. Southern Leyte 12. Surigao del Sur 13. Western Samar 14. Zamboanga del Sur 15. Zamboanga Sibugay |

MDA - mass drug administration, TAS - transmission assessment survey
